# Supplementary material for: Gait smoothness during high-demand motor walking tasks in older adults with mild cognitive impairment
Source: PLoS One. 2024 Jan 19;19(1):e0296710. doi: 10.1371/journal.pone.0296710 (PMC10798528; doi:10.1371/journal.pone.0296710)
Supplement: S1 File — Mean, standard deviation, maximum, and minimum values of index of harmonicity of AP, ML, and VT directions for each individual under each complex motor walking task: Walking a narrow path, walking around an obstacle, horizontal head turns while walking, and vertical head turns while walking. (PDF) [file pone.0296710.s001.pdf]

ID	Group	Baseline				Walking a narrow path				Walking around an obstacle				Horizontal head turns while walking				Vertical head turns while walking			
		IH-AP	IH-ML	IH-VT	Gait speed (m/s)	IH-AP	IH-ML	IH-VT	Gait speed (m/s)	IH-AP	IH-ML	IH-VT	Gait speed (m/s)	IH-AP	IH-ML	IH-VT	Gait speed (m/s)	IH-AP	IH-ML	IH-VT	Gait speed (m/s)
Control01	1	0.740	0.835	0.880	1.215	0.770	0.785	0.045	0.990	0.145	0.355	0.120	0.255	0.085	0.835	0.870	0.945	0.545	0.715	0.745	0.970
Control02	1	0.590	0.675	0.835	1.180	0.725	0.790	0.060	1.155	0.040	0.240	0.130	0.330	0.155	0.815	0.835	1.020	0.760	0.860	0.815	0.965
Control03	1	0.745	0.755	0.900	1.040	0.805	0.820	0.130	0.965	0.135	0.530	0.340	0.250	0.340	0.690	0.645	0.815	0.295	0.450	0.435	0.815
Control04	1	0.550	0.665	0.845	0.940	0.665	0.595	0.235	0.905	0.290	0.485	0.290	0.270	0.195	0.680	0.790	0.875	0.660	0.790	0.795	0.910
Control05	1	0.785	0.840	0.865	0.980	0.860	0.895	0.320	0.860	0.180	0.690	0.770	0.260	0.870	0.870	0.660	0.640	0.835	0.805	0.845	0.675
Control06	1	0.650	0.690	0.655	1.400	0.630	0.845	0.355	1.160	0.430	0.365	0.310	0.340	0.575	0.810	0.810	1.100	0.730	0.755	0.940	1.110
Control07	1	0.755	0.785	0.880	0.880	0.815	0.835	0.335	0.790	0.195	0.725	0.535	0.240	0.675	0.710	0.705	0.820	0.610	0.840	0.905	0.810
Control08	1	0.730	0.630	0.825	1.220	0.710	0.770	0.440	1.375	0.230	0.500	0.460	0.390	0.385	0.905	0.910	1.065	0.945	0.875	0.865	1.000
Control09	1	0.760	0.900	0.770	0.975	0.845	0.810	0.160	0.560	0.350	0.635	0.560	0.210	0.640	0.630	0.405	0.705	0.370	0.560	0.800	0.785
Control10	1	0.675	0.810	0.905	0.950	0.790	0.755	0.130	0.770	0.075	0.580	0.275	0.195	0.270	0.575	0.440	0.725	0.325	0.525	0.530	0.710
Control11	1	0.615	0.700	0.885	1.240	0.680	0.590	0.005	1.165	0.015	0.330	0.015	0.330	0.010	0.520	0.435	1.115	0.325	0.510	0.600	1.130
Control12	1	0.660	0.670	0.855	1.200	0.705	0.720	0.410	1.135	0.340	0.650	0.370	0.275	0.495	0.645	0.685	0.890	0.625	0.530	0.640	0.845
Control13	1	0.805	0.870	0.865	1.035	0.870	0.845	0.400	0.660	0.290	0.620	0.615	0.195	0.490	0.925	0.765	0.780	0.725	0.885	0.805	0.740
Control14	1	0.645	0.680	0.860	0.970	0.815	0.600	0.280	1.045	0.160	0.590	0.345	0.270	0.205	0.735	0.660	0.895	0.715	0.630	0.705	1.000
Control15	1	0.690	0.800	0.895	1.165	0.710	0.710	0.115	0.920	0.065	0.450	0.430	0.260	0.225	0.575	0.430	0.925	0.490	0.715	0.530	0.900
Control16	1	0.695	0.695	0.890	1.060	0.710	0.730	0.115	1.000	0.115	0.575	0.150	0.235	0.145	0.815	0.760	0.895	0.660	0.865	0.860	0.950
Control17	1	0.525	0.725	0.840	1.230	0.695	0.745	0.135	0.880	0.165	0.685	0.415	0.240	0.435	0.810	0.675	0.705	0.460	0.445	0.475	0.755
Control18	1	0.680	0.760	0.885	1.300	0.800	0.815	0.140	1.190	0.070	0.460	0.460	0.270	0.375	0.720	0.850	0.855	0.840	0.795	0.785	0.860
**Means**		**0.683**	**0.749**	**0.852**	**1.110**	**0.756**	**0.759**	**0.212**	**0.974**	**0.183**	**0.526**	**0.366**	**0.268**	**0.365**	**0.737**	**0.685**	**0.876**	**0.606**	**0.697**	**0.726**	**0.885**
**SD**		**0.079**	**0.080**	**0.059**	**0.147**	**0.072**	**0.089**	**0.137**	**0.206**	**0.118**	**0.138**	**0.191**	**0.052**	**0.229**	**0.119**	**0.161**	**0.138**	**0.194**	**0.157**	**0.154**	**0.131**
**Max**		**0.805**	**0.900**	**0.905**	**1.400**	**0.870**	**0.895**	**0.440**	**1.375**	**0.430**	**0.725**	**0.770**	**0.390**	**0.870**	**0.925**	**0.910**	**1.115**	**0.945**	**0.885**	**0.940**	**1.130**
**Min**		**0.525**	**0.630**	**0.655**	**0.880**	**0.630**	**0.590**	**0.005**	**0.560**	**0.015**	**0.240**	**0.015**	**0.195**	**0.010**	**0.520**	**0.405**	**0.640**	**0.295**	**0.445**	**0.435**	**0.675**
MCI01	2	0.660	0.610	0.830	1.030	0.715	0.730	0.125	1.105	0.060	0.255	0.200	0.330	0.230	0.660	0.640	0.855	0.805	0.650	0.655	0.915
MCI02	2	0.720	0.675	0.840	1.155	0.615	0.800	0.155	1.060	0.180	0.630	0.375	0.260	0.540	0.765	0.785	1.020	0.565	0.710	0.765	0.780
MCI03	2	0.755	0.810	0.900	1.045	0.880	0.860	0.120	0.945	0.095	0.635	0.290	0.215	0.330	0.760	0.710	0.760	0.440	0.675	0.580	0.830
MCI04	2	0.495	0.515	0.760	1.165	0.640	0.565	0.150	1.115	0.145	0.460	0.375	0.275	0.360	0.610	0.630	1.095	0.595	0.730	0.750	1.060
MCI05	2	0.710	0.750	0.805	0.850	0.695	0.775	0.500	0.930	0.310	0.525	0.370	0.240	0.500	0.345	0.385	1.025	0.625	0.730	0.755	0.920
MCI06	2	0.760	0.800	0.885	0.655	0.835	0.790	0.240	0.615	0.075	0.320	0.300	0.170	0.240	0.540	0.560	0.610	0.340	0.670	0.460	0.580
MCI07	2	0.815	0.885	0.810	0.910	0.830	0.795	0.195	0.820	0.470	0.100	0.145	0.225	0.380	0.675	0.500	0.790	0.565	0.605	0.450	0.785
MCI08	2	0.770	0.710	0.825	0.900	0.725	0.595	0.300	0.955	0.355	0.465	0.355	0.330	0.110	0.830	0.735	0.770	0.740	0.900	0.805	0.675
MCI09	2	0.680	0.860	0.890	1.080	0.710	0.695	0.035	0.430	0.085	0.495	0.135	0.305	0.135	0.610	0.560	0.850	0.615	0.545	0.450	0.805
MCI10	2	0.805	0.905	0.875	0.720	0.915	0.890	0.170	0.495	0.085	0.485	0.665	0.195	0.400	0.540	0.345	0.505	0.600	0.480	0.450	0.550
MCI11	2	0.625	0.855	0.855	0.905	0.630	0.675	0.185	0.690	0.125	0.595	0.185	0.245	0.390	0.390	0.355	0.885	0.305	0.380	0.495	0.855
MCI12	2	0.735	0.835	0.880	1.080	0.855	0.830	0.195	0.750	0.065	0.470	0.520	0.260	0.345	0.835	0.720	0.755	0.755	0.770	0.780	0.830
MCI13	2	0.795	0.785	0.825	0.960	0.855	0.805	0.235	0.535	0.250	0.495	0.470	0.210	0.315	0.725	0.580	0.765	0.600	0.700	0.565	0.715
MCI14	2	0.610	0.735	0.750	1.105	0.600	0.605	0.465	0.880	0.405	0.645	0.500	0.285	0.500	0.815	0.785	0.965	0.865	0.830	0.815	1.015
MCI15	2	0.470	0.680	0.785	1.540	0.580	0.600	0.220	1.125	0.110	0.600	0.250	0.320	0.235	0.800	0.895	1.100	0.750	0.830	0.865	1.155
MCI16	2	0.650	0.675	0.860	1.140	0.790	0.730	0.045	1.170	0.020	0.220	0.085	0.290	0.080	0.560	0.595	0.860	0.245	0.550	0.445	0.865
MCI17	2	0.755	0.735	0.885	0.855	0.865	0.790	0.435	0.935	0.395	0.855	0.760	0.255	0.795	0.325	0.360	0.675	0.540	0.380	0.485	0.825
MCI18	2	0.515	0.650	0.805	1.195	0.605	0.560	0.420	1.095	0.385	0.635	0.515	0.335	0.585	0.670	0.770	0.940	0.825	0.705	0.675	1.005
**Means**		**0.685**	**0.748**	**0.837**	**1.016**	**0.741**	**0.727**	**0.233**	**0.869**	**0.201**	**0.494**	**0.361**	**0.264**	**0.359**	**0.636**	**0.606**	**0.846**	**0.599**	**0.658**	**0.625**	**0.843**
**SD**		**0.106**	**0.104**	**0.045**	**0.202**	**0.113**	**0.105**	**0.139**	**0.235**	**0.147**	**0.181**	**0.185**	**0.049**	**0.181**	**0.162**	**0.167**	**0.162**	**0.179**	**0.146**	**0.153**	**0.157**
**Max**		**0.815**	**0.905**	**0.900**	**1.540**	**0.915**	**0.890**	**0.500**	**1.170**	**0.470**	**0.855**	**0.760**	**0.335**	**0.795**	**0.835**	**0.895**	**1.100**	**0.865**	**0.900**	**0.865**	**1.155**
**Min**		**0.470**	**0.515**	**0.750**	**0.655**	**0.580**	**0.560**	**0.035**	**0.430**	**0.020**	**0.100**	**0.085**	**0.170**	**0.080**	**0.325**	**0.345**	**0.505**	**0.245**	**0.380**	**0.445**	**0.550**

Abbreviation

IH	Index of harmonicity
AP	Anteroposterior
ML	Mediolateral
VT	Vertical
