# Supplementary material for: Gait smoothness during high-demand motor walking tasks in older adults with mild cognitive impairment
Source: PLoS One. 2024 Jan 19;19(1):e0296710. doi: 10.1371/journal.pone.0296710 (PMC10798528; doi:10.1371/journal.pone.0296710)
Supplement: Attachment — Submitted filename: PONE-D-23-25030_reviewer_reviewer comments.pdf [file pone.0296710.s002.pdf]

# PLOS ONE

# Gait smoothness during high-demand motor walking tasks in older adults with mild cognitive impairment

--Manuscript Draft--

| | |
|---|---|
| **Manuscript Number:** | PONE-D-23-25030 |
| **Article Type:** | Research Article |
| **Full Title:** | Gait smoothness during high-demand motor walking tasks in older adults with mild cognitive impairment |
| **Short Title:** | Gait smoothness during high-demand motor walking tasks in MCI |
| **Corresponding Author:** | Sirinun Boripuntakul, Ph.D.<br>Chiang Mai University<br>Chiang Mai District, THAILAND |
| **Keywords:** | gait smoothness; mild cognitive impairment; motor load tasks; complex walking task; head turning while walking; index of harmonicity |
| **Abstract:** | Early signs of Mild Cognitive Impairment (MCI)-related gait deficits may be detected through the performance of complex walking tasks that require high postural control. Gait smoothness is a robust metric of overall body stability during walking. This study aimed to evaluate gait smoothness during complex walking tasks in older adults with and without MCI. Participants were 18 older adults with MCI (mean age = 67.89 ± 4.64 years) and 18 cognitively intact controls (mean age = 67.72 ± 4.63 years). Gait assessment under complex walking tasks comprises two types of motor tasks: constrained walking (i.e., walking a narrow path, walking around an obstacle) and head turning while walking (i.e., horizontal head turns, vertical head turns). The index of harmonicity (IH), representing gait smoothness associated with overall body stability, was measured in anteroposterior, mediolateral, and vertical directions. An independent sample t-test was conducted to compare the differences in IH between groups under constrained walking tasks and head-turning walking tasks. The MCI group demonstrated a reduction of IH in the mediolateral direction during the horizontal head turns than the control group (MCI group = 0.64 ± 0.16, Control group = 0.74 ± 0.12, p = 0.04). No significant differences between groups were found for the IH in other directions or walking conditions. These findings indicate that older adults with MCI have a decline in step regularity in the mediolateral direction during walking with horizontal head turns. Assessment of the smoothness of walking during head turns may be a promising approach to identifying subtle gait alterations in older adults with MCI, which may facilitate timely intervention and prevention of falls. |
| **Order of Authors:** | Thanpidcha Poosri, Bachelors |
| | Sirinun Boripuntakul, Ph.D. |
| | Somporn Sungkarat, Ph.D. |
| | Teerawat Kamnardsiri, Ph.D. |
| | Atiwat Soontornpun, M.D. |
| | Kanokporn Pinyopornpanish, M.D. |
| **Additional Information:** | |
| **Question** | **Response** |
| **Financial Disclosure**<br><br>Enter a financial disclosure statement that describes the sources of funding for the work included in this submission. Review the submission guidelines for detailed requirements. View published research | -Initials of the authors who received each award: Thanpidcha Poosri<br>-Grant numbers awarded to each author: AMS-2564<br>-The full name of each funder: Faculty of Associated Medical Sciences, Chiang Mai University, Thailand.<br>-URL of each funder website: N/A<br>-Did the sponsors or funders play any role in the study design, data collection and analysis, decision to publish, or preparation of the manuscript?: The funders had no |

| | |
|---|---|
| articles from *PLOS ONE* for specific examples.<br><br>This statement is required for submission and **will appear in the published article** if the submission is accepted. Please make sure it is accurate.<br><br>**Unfunded studies**<br>Enter: *The author(s) received no specific funding for this work.*<br><br>**Funded studies**<br>Enter a statement with the following details:<br>• Initials of the authors who received each award<br>• Grant numbers awarded to each author<br>• The full name of each funder<br>• URL of each funder website<br>• Did the sponsors or funders play any role in the study design, data collection and analysis, decision to publish, or preparation of the manuscript?<br>• **NO** - Include this sentence at the end of your statement: *The funders had no role in study design, data collection and analysis, decision to publish, or preparation of the manuscript.*<br>• **YES** - Specify the role(s) played.<br><br>* typeset | role in study design, data collection and analysis, decision to publish, or preparation of the manuscript. |
| **Competing Interests**<br><br>Use the instructions below to enter a competing interest statement for this submission. On behalf of all authors, disclose any competing interests that could be perceived to bias this work—acknowledging all financial support and any other relevant financial or non-financial competing interests.<br><br>This statement is required for submission and **will appear in the published article** if the submission is accepted. Please make sure it is accurate and that any funding sources listed in your Funding Information later in the submission form are also declared in your Financial Disclosure statement. | The authors have declared that no competing interests exist. |

| | |
|---|---|
| View published research articles from *PLOS ONE* for specific examples.<br><br>**NO authors have competing interests**<br><br>Enter: *The authors have declared that no competing interests exist.*<br><br>**Authors with competing interests**<br><br>Enter competing interest details beginning with this statement:<br><br>*I have read the journal's policy and the authors of this manuscript have the following competing interests: [insert competing interests here]*<br><br>* typeset | |
| **Ethics Statement**<br><br>Enter an ethics statement for this submission. This statement is required if the study involved:<br><br>• Human participants<br>• Human specimens or tissue<br>• Vertebrate animals or cephalopods<br>• Vertebrate embryos or tissues<br>• Field research<br><br>Write "N/A" if the submission does not require an ethics statement.<br><br>General guidance is provided below. Consult the submission guidelines for detailed instructions. **Make sure that all information entered here is included in the Methods section of the manuscript.** | Human Subject Research:<br>-Human Research Ethics Unit, Faculty of Associated Medical Sciences, Chiang Mai University, Thailand<br>-Approval number: AMSEC-65EX-025<br>-Form of consent obtained: written |

| | |
|---|---|
| **Format for specific study types**<br><br>**Human Subject Research (involving human participants and/or tissue)**<br>• Give the name of the institutional review board or ethics committee that approved the study<br>• Include the approval number and/or a statement indicating approval of this research<br>• Indicate the form of consent obtained (written/oral) or the reason that consent was not obtained (e.g. the data were analyzed anonymously)<br><br>**Animal Research (involving vertebrate animals, embryos or tissues)**<br>• Provide the name of the Institutional Animal Care and Use Committee (IACUC) or other relevant ethics board that reviewed the study protocol, and indicate whether they approved this research or granted a formal waiver of ethical approval<br>• Include an approval number if one was obtained<br>• If the study involved *non-human primates*, add *additional details* about animal welfare and steps taken to ameliorate suffering<br>• If anesthesia, euthanasia, or any kind of animal sacrifice is part of the study, include briefly which substances and/or methods were applied<br><br>**Field Research**<br><br>Include the following details if this study involves the collection of plant, animal, or other materials from a natural setting:<br>• Field permit number<br>• Name of the institution or relevant body that granted permission | |
| **Data Availability**<br><br>Authors are required to make all data underlying the findings described fully available, without restriction, and from the time of publication. PLOS allows rare exceptions to address legal and ethical concerns. See the PLOS Data Policy and FAQ for detailed information. | Yes - all data are fully available without restriction |

| | |
|---|---|
| A Data Availability Statement describing where the data can be found is required at submission. Your answers to this question constitute the Data Availability Statement and **will be published in the article**, if accepted.<br><br>**Important:** Stating 'data available on request from the author' is not sufficient. If your data are only available upon request, select 'No' for the first question and explain your exceptional situation in the text box.<br><br>Do the authors confirm that all data underlying the findings described in their manuscript are fully available without restriction? | |
| **Describe where the data may be found in full sentences. If you are copying our sample text, replace any instances of XXX with the appropriate details.**<br><br>• If the data are **held or will be held in a public repository**, include URLs, accession numbers or DOIs. If this information will only be available after acceptance, indicate this by ticking the box below. For example: *All XXX files are available from the XXX database (accession number(s) XXX, XXX.*).<br>• If the data are all contained **within the manuscript and/or Supporting Information files**, enter the following: *All relevant data are within the manuscript and its Supporting Information files.*<br>• If neither of these applies but you are able to provide **details of access elsewhere**, with or without limitations, please do so. For example:<br><br>*Data cannot be shared publicly because of [XXX]. Data are available from the XXX Institutional Data Access / Ethics Committee (contact via XXX) for researchers who meet the criteria for access to confidential data.*<br><br>*The data underlying the results presented in the study are available from (include the name of the third party* | All relevant data are within the manuscript and its Supporting Information files. |

| | |
|---|---|
| *and contact information or URL).*<br>• This text is appropriate if the data are owned by a third party and authors do not have permission to share the data.<br><br>* typeset | |
| Additional data availability information: | Tick here if the URLs/accession numbers/DOIs will be available only after acceptance of the manuscript for publication so that we can ensure their inclusion before publication. |

Powered by Editorial Manager® and ProduXion Manager® from Aries Systems Corporation

Manuscript Click here to access/download;Manuscript;Manuscript.docx

# Gait smoothness during high-demand motor walking tasks in older adults with mild cognitive impairment

Thanpidcha Poosri[1], Sirinun Boripuntakul[1*], Somporn Sungkarat[1], Teerawat Kamnardsiri[2], Atiwat Soontornpun[3], Kanokporn Pinyopornpanish[4]

[1] Department of Physical Therapy, Faculty of Associated Medical Sciences, Chiang Mai University, Thailand

[2] Department of Digital Game, College of Arts, Media and Technology, Chiang Mai University, Thailand

[3] Department of Internal Medicine, Faculty of Medicine, Chiang Mai University and Northern Neuroscience Center, Thailand

[4] Department of Family Medicine, Faculty of Medicine, Chiang Mai University, Thailand

* Corresponding author

E-mail: sirinun.b@cmu.ac.th (SB)

# Abstract

Early signs of Mild Cognitive Impairment (MCI)-related gait deficits may be detected through the performance of complex walking tasks that require high postural control. Gait smoothness is a robust metric of overall body stability during walking. This study aimed to evaluate gait smoothness during complex walking tasks in older adults with and without MCI. Participants were 18 older adults with MCI (mean age = 67.89 ± 4.64 years) and 18 cognitively intact controls (mean age = 67.72 ± 4.63 years). Gait assessment under complex walking tasks comprises two types of motor tasks: constrained walking (i.e., walking a narrow path, walking around an obstacle) and head turning while walking (i.e., horizontal head turns, vertical head turns). The index of harmonicity (IH), representing gait smoothness associated with overall body stability, was measured in anteroposterior, mediolateral, and vertical directions. An independent sample t-test was conducted to compare the differences in IH between groups under constrained walking tasks and head-turning walking tasks. The MCI group demonstrated a reduction of IH in the mediolateral direction during the horizontal head turns than the control group (MCI group = 0.64 ± 0.16, Control group = 0.74 ± 0.12, p = 0.04). No significant differences between groups were found for the IH in other directions or walking conditions. These findings indicate that older adults with MCI have a decline in step regularity in the mediolateral direction during walking with horizontal head turns. Assessment of the smoothness of walking during head turns may be a promising approach to identifying subtle gait alterations in older adults with MCI, which may facilitate timely intervention and prevention of falls.

# Introduction

Higher level cognitive function, specifically executive function, and attention, plays a vital role in integrating sensorimotor information and controlling movement execution, contributing to safe mobility in complex real-life environments [1, 2]. Previous studies have found that individuals with Mild Cognitive Impairment (MCI), a clinical entity involving cognitive deficit beyond normal aging [3, 4], displayed a decline in attention and executive function [5] which may in turn res[illegible]ng in gait abnormalities and increased risk of falling [6, 7]. Therefore, early identification of subtle MCI-related changes in gait may have important clinical benefits for fall prevention.

Existing studies have demonstrated that simple steady-state walking tasks allow the detection of gait changes related to falls in older adults with MCI compared to their cognitively intact peers. [4, 8, 9]. While the above findings were promising, some studies failed to find differences in gait features between persons with MCI and those cognitively intact controls [4, 9, 10]. Discrepancies across prior findings may be due to the variations in gait tasks and outcome measures. It is posited that complex walking tasks could exacerbate subtle MCI-related gait impairment. Currently, it is well documented that the gait impairment of individuals with MCI is significantly pronounced when adding a cognitive load while walking (dual-tasking) [9]. However, previous studies examining MCI-related changes in gait under motor load tasks relevant to a real-life walking situation are scarce. Limited studies have demonstrated a decline in gait regulation of older adults with MCI during transitional gait speeds, including gait initiation, termination, and slow speed transition [11-13]. These findings suggest that motor load tasks may be sensitive in detecting subtle gait changes in older adults with MCI.

Head turning is a fundamental component for successful responses to environmental demands via visual scanning, such as checking for traffic before crossing the road and navigating

the barriers in the travel path [14]. Basically, head turning while walking alters visual and vestibular information from the changes of visual field. Specifically, when individuals need to adjust their gait in real-time while head-turning in response to environmental constraints, the vestibular system is more burdened with tracing head movements, which may compromise gait stability [15]. Previous studies have elicited a reduction in postural stability in individuals with MCI partly due to a decrement in the vestibular system linked to a decline in hippocampus function [16]. Therefore, it is plausible that walking stability in people with MCI would be more noticeable when coping with head manipulation while walking. Moreover, constrained walking tasks concerning everyday environments that require motor demands in older adults, including walking a narrow path and around an obstacle [17, 18], may challenge gait control in older adults with MCI. Taken together, head-turning and constrained walking tasks that tap into motor demands may be used as a motor stress test to amplify a subtle change of gait in older adults with MCI.

The smoothness of walking, the rhythmic pattern of trunk acceleration, is a robust quality metric useful to quantify gait regularity and whole-body dynamic stability during walking, contributing to the risk of falls [19-21]. Prior works have demonstrated impaired gait smoothness across various populations, such as cognitively impaired individuals, older adults with restricted neuromuscular control, Parkinson's disease patients, and individuals with sensory impairment, compared to young adults and persons without the disease [21-27]. According to recent studies, trunk accelerations are more sensitive than traditional spatiotemporal metrics for capturing subtle alterations in gait [26, 28]. Thus, it could be considered a candidate measure of gait stability in older adults with early cognitive limitations.

To date, gait smoothness has been used to examine gait quality in limited groups and walking conditions. In addition, to the best of our knowledge, no study has investigated the smoothness of gait in people with MCI under high-demand motor tasks relevant to real-life environmental

conditions. The aim of this study, therefore, was to compare gait smoothness during two types of outdoor walking terrain, including constrained walking tasks (i.e., walking a narrow path and around an obstacle) and head turning while walking tasks (i.e., walking in concurrent with horizontal and vertical head turns) between older adults with and without MCI in a case-control study. It was hypothesized that the MCI group would have less gait smoothness under motor load tasks than the control group. The present findings may provide valuable clinical implications for detecting early signs of gait alterations and potential fall risk in older adults with MCI.

# Material and methods

## Participants

The sample size was calculated based on our pilot study, in which the smallest effect size was chosen. The smallest effect size of 0.22 was derived from the index of harmonicity (IH) in the anteroposterior direction in the condition of walking while the vertical head turns (with baseline gait speed as a covariate). With this, a general linear model with an effect size of 0.22, a power of 0.80, and an alpha level of 0.05 revealed a total sample size of at least 26 participants (13 participants per group).

Eighteen community-dwelling older adults with MCI and 18 healthy-matched controls aged 60 or older were enrolled in the present study. The older adults with MCI were recruited from Maharaj Nakorn Chiang Mai Hospital, a tertiary care hospital in Northern Thailand. The diagnosis of MCI was performed by an experienced neurologist based on Petersen’s criteria [29]: (i) subjective complaint of decline in memory on self-or informant report, (ii) isolated memory deficits (determined by the 10-word list learning test ≤ 5) [30], (iii) essentially preserved general cognitive function (determined by the Mental State Examination T10 (MSET10) > 22 or adjusted for

educational level) [31], (iv) largely intact activities of daily living using the clinical review with the patients and informant interview, and (v) clinical dementia (determined by the global Clinical Dementia Rating (CDR) scale < 1) [32]. Additionally, all participants with MCI were required to score on the Montreal Cognitive Assessment (MoCA) ≤ 24 to affirm the presence of subtle cognitive impairment [33].

The cognitively intact control group with similar age (plus or minus two years), gender, body mass index (BMI) (plus or minus two units), and education level (plus or minus two years) was recruited from Chiang Mai community via local community groups and social media advertisements (e.g., Facebook). To be eligible, all participants in the control group were required to have intact cognitive function, as identified by the normal scores of the MSET10 and MoCA.

The inclusion criteria for both groups were being able to walk independently and safely without an assistive device for at least 10 meters and being able to comprehend and follow the instructions. The exclusion criteria were: (i) had major health conditions that affect walking ability or safety during testing such as musculoskeletal, neurological, cardiorespiratory problems or any unstable medical conditions (e.g., Parkinson’s disease, stroke, peripheral vertigo, acute joint pain, uncorrected visual and/or hearing impairment), (ii) had severe dizziness when turning the head in horizontal and/or vertical directions, (iii) had depressive symptoms (determined by the Thai Geriatric Depression Scale-15 score > 6) [34], (iv) current use of sedative drugs, antipsychotic drugs, and narcotic drugs, and (v) had leg length discrepancy > 2.5 cm (assessed from the anterior superior iliac spine (ASIS) to the tip of medial malleolus in a standing position using a flexible tape measure) [35].

The study was approved by the institution’s ethical review committee for research in humans (approval number: AMSEC-65EX-025). The start and end of the recruitment period for this study were from the 26$^{th}$ of August 2022 to the 9$^{th}$ of April 2023. All participants provided written

informed consent prior to enrolling in the study. Before gait testing, participants in both groups were interviewed about personal demographic data, including age, weight, height, education level, fall incidence within the past year, medical conditions, and medication used. Additionally, participants underwent an assessment of attention and executive functions, which were previously determined to be closely related to gait control, using the Digit Span forward and backward tests and Trail Making Test Parts A and B, respectively [36, 37].

## Assessment of gait smoothness under complex motor walking tasks

A triaxial accelerometer device (DynaPort MoveMonitor, McRoberts, The Hague, The Netherlands) with a sampling frequency of 100 Hz was used to measure the IH, which is an indicator of gait smoothness along three orthogonal axes, including anteroposterior (AP), mediolateral (ML), and vertical (VT) directions [38]. The accelerometer was mounted to the participants' fifth lumbar spine level and fastened with the supplied elastic belt [39] (Fig 1). The trunk acceleration signal during walking was transmitted in real-time via Bluetooth to a software database on the personal computer, where the signal-based parameters including IH were subsequently analyzed by an algorithm developed by Micó-Amigo et al. [40].

**Fig 1. Placement of triaxial accelerometer on the lower back at the level of the fifth lumbar vertebra.**

After a triaxial accelerometer setting, participants were requested to walk along a 10-meter walkway at their comfortable speed for two trials to use as baseline data. As for the gait testing protocol, participants were evaluated for gait smoothness under two types of complex motor walking tasks on the outdoor walkway: Task I: constrained walking tasks, including walking a

narrow path and walking around an obstacle (Fig 2), and Task II: head-turning walking tasks, including walking in concurrent with horizontal head turns and walking in concurrent with vertical head turns (Fig 3). For all walking tasks, participants were asked to walk at a self-selected speed over a 10-meter distance for 2 trials in each condition (for a total of 8 trials). The two types of complex motor walking tasks were randomized. A 1-meter distance from the beginning and end of the walkway was excluded from the data analysis to discourage gait acceleration and deceleration events. Participants were given a demonstration before testing, and standard instructions were provided throughout the trials. A 1-minute resting period was allowed in each trial.

**Task I: Constrained walking tasks**

A. Walking a narrow path (WN): Participants were asked to walk within the lines marked with tape on the floor. The wide path was set individually at 50% of the distance between each participant's ASIS [41].

B. Walking around an obstacle (WO): Participants were instructed to alternately walk around the cones on both the right and left sides (zig-zag patterns). Each cone was placed at a distance of 61 cm in width and 75 cm in length [42].

**Fig 2. Gait assessment under constrained walking tasks: (A) Walking a narrow path, (B) Walking around an obstacle.**

**Task II: Head turning while walking tasks**

A. Walking in concurrent with horizontal head turns (W-HH): Participants were asked to continue walking straight while turning their heads once to the right and then once to the left, respectively [43]. The assessor instructed the participants to “look right” or “look left” in consecutive order, every 1 meter.

B. Walking in concurrent with vertical head turns (W-VH): Participants were requested to keep walking straight while alternately tipping their heads up and down. The assessor instructed the participants to “look up” or “look down” in consecutive order, every 1 meter.

**Fig 3. Gait assessment under head-turning walking tasks: (A) Walking while horizontal head turns, (B) Walking while vertical head turns.**

## Gait smoothness definition

Gait smoothness, expressed as the IH, is a metric of gait quality that determines the intersegmental coordination of trunk and pelvic movement within a stride by exploiting the periodicity of the signal [20]. The IH was calculated from the power of the first six harmonics estimated through a discrete Fourier transform (DFT). As calculated by a DFT, is the power spectral density of the fundamental frequency (first harmonic) and is the cumulative sum of the powers of the fundamental frequency and the first five superharmonics (multiplication of the fundamental frequency) (Equations 1-3). A power ratio of 1 denotes perfect gait smoothness [20]. The IH measures while performing complex motor walking tasks were quantified into three directions of acceleration: AP, ML, and VT.

$$IH_{AP} = \frac{P_{0\_AP}}{\sum_{i=0}^{5} P\_AP_i} \quad (1)$$

$$IH_{ML} = \frac{P_{0\_ML}}{\sum_{i=0}^{5} P\_ML_i} \quad (2)$$

$$IH_{VT} = \frac{P_{0\_VT}}{\sum_{i=0}^{5} P\_VT_i} \quad (3)$$

## Statistical analysis

SPSS software for macOS version 26 (IBM, Armonk, NY) was used to perform the statistical analyses. An independent sample t-test (continuous data) and Chi-Square test (categorial data) were used to compare the participant demographic profiles between the MCI and control groups. The Shapiro-Wilk test was used to determine data normality. An independent sample t-test was employed to address the differences in IH (i.e., AP, ML, and VT) between the two groups for each constrained walking task (i.e., WN, WO) and head-turning walking task (i.e., W-HH, W-VH). A significance level was set at $p < 0.05$, two-sided.

# Results

## Participant characteristics

The demographics, cognitive score tests, and baseline gait smoothness of the MCI and control groups are shown in Table 1. There were no significant differences between groups in demographic characteristics ($p > 0.05$). As for cognitive performance, the MCI group had lower scores on MoCA ($p = 0.001$) and Digit Span test than the control group ($p = 0.01$). Gait speed and IH in all directions (i.e., AP, ML, and VT) at baseline assessment were similar between the two groups ($p > 0.05$).

**Table 1. Participant characteristics in the control and MCI groups (N = 36).**

| Characteristics | Control (n = 18) | MCI (n = 18) | p-value |
|---|---|---|---|
| Age (yr) | 67.72 ± 4.63 | 67.89 ± 4.64 | 0.92 |
| Gender (Male: Female, n) | 6:12 | 6: 12 | 1.00 |
| Body mass index (kg/m$^2$) | 24.84 ± 3.12 | 24.17 ± 2.9224 | 0.51 |
| Education level (yr) | 15.89 ± 3.16 | 15.22 ± 3.44 | 0.55 |
| Number of medications | 1.17 ± 1.20 | 1.56 ± 1.15 | 0.33 |
| Number of falls in the past year | 0.22 ± 0.55 | 0.28 ± 0.75 | 0.80 |
| MSET10 (score, total score = 29) | 27.00 ± 1.24 | 26.67 ± 1.33 | 0.44 |
| MoCA (score, total score = 30) | 26.22 ± 1.52 | 22.00 ± 1.94 | 0.001[a] |
| DST (score, total score = 28) | 17.11 ± 3.45 | 14.39 ± 2.83 | 0.01[a] |
| TMT A (sec) | 45.74 ± 15.48 | 49.11 ± 18.76 | 0.56 |
| TMT B (sec) | 104.64 ± 38.14 | 121.14 ± 41.72 | 0.22 |
| TMT B-A (sec) | 58.90 ± 34.36 | 72.03 ± 40.72 | 0.30 |
| Baseline IH-AP | 0.68 ± 0.08 | 0.68 ± 0.10 | 0.96 |
| Baseline IH-ML | 0.75 ± 0.08 | 0.75 ± 0.10 | 0.98 |
| Baseline IH-VT | 0.85 ± 0.06 | 0.84 ± 0.05 | 0.46 |
| Baseline gait speed (m/s) | 1.11 ± 0.15 | 1.02 ± 0.20 | 0.12 |

All values are shown as mean ± standard deviation except for gender, number of medications, and falls in the past year.

Independent sample t-test for continuous data and Chi-square test for categorical data.

[a] Significant difference between two groups, $p < 0.05$

MSET10 = Mental State Examination T10; MoCA = Montreal Cognitive Assessment; DST = Digit Span Test; TMT A = Trail Making Test Part A; TMT B = Trail Making Test Part B; TMT B-A = subtracting Part B from Part A; IH-AP = Index of harmonicity in the anteroposterior direction; IH-ML = Index of harmonicity in the mediolateral direction; IH-VT = Index of harmonicity in the vertical direction

# Gait smoothness during walking under complex motor tasks

## Task I: Constrained walking tasks

The mean and standard deviation of the IH between groups for the WN and WO conditions are presented in Table 2. The independent sample t-test revealed no significant difference for IH in all directions between the MCI and control groups in the WN and WO conditions ($p > 0.05$). In addition, no significant differences were found between both groups in gait speed under the WN and WO conditions.

## Task II: Head-turning walking tasks

The IH values of the MCI and control groups walking in concurrent with head turn conditions (i.e., W-HH, W-VH) are shown in Table 3. The independent sample t-test revealed a significant difference of the IH-ML in the W-HH condition ($p = 0.04$) between the two groups, in which the MCI group had a lower IH-ML value when compared to the control group. There were no significant differences in all other IH directions and head-turning conditions between the two groups. Moreover, gait speed in both the W-HH and W-HV was similar between the two groups ($p > 0.05$).

**Table 2. The IH values of the MCI and control groups under the constrained walking tasks.**

| IH | Control group (n = 18) | MCI group (n = 18) | p-value |
|---|---|---|---|
| **Walking a narrow path** | | | |
| IH-AP | 0.76 ± 0.07 | 0.74 ± 0.11 | 0.65 |
| IH-ML | 0.76 ± 0.09 | 0.73 ± 0.11 | 0.34 |
| IH-VT | 0.21 ± 0.14 | 0.23 ± 0.14 | 0.65 |
| Gait speed (m/s) | 0.97 ± 0.21 | 0.87 ± 0.23 | 0.17 |
| **Walking around an obstacle** | | | |
| IH-AP | 0.18 ± 0.12 | 0.20 ± 0.15 | 0.85 |
| IH-ML | 0.53 ± 0.14 | 0.49 ± 0.18 | 0.55 |
| IH-VT | 0.37 ± 0.19 | 0.36 ± 0.19 | 0.93 |
| Gait speed (m/s) | 0.27 ± 0.05 | 0.26 ± 0.05 | 0.82 |

All values are shown as mean ± standard deviation.

IH = Index of harmonicity; IH-AP = Index of harmonicity in the anteroposterior direction; IH-ML = Index of harmonicity in the mediolateral direction; IH-VT = Index of harmonicity in the vertical direction

**Table 3. The IH values of the MCI and control groups under the head-turning walking tasks.**

| IH | Control group (n = 18) | MCI group (n = 18) | p-value |
|---|---|---|---|
| **Horizontal head turns while walking** | | | |
| IH-AP | 0.37 ± 0.23 | 0.36 ± 0.18 | 0.94 |
| IH-ML | 0.74 ± 0.12 | 0.64 ± 0.16 | 0.04[a] |
| IH-VT | 0.69 ± 0.16 | 0.61 ± 0.17 | 0.16 |
| Gait speed (m/s) | 0.88 ± 0.14 | 0.85 ± 0.16 | 0.55 |
| **Vertical head turns while walking** | | | |
| IH-AP | 0.61 ± 0.19 | 0.60 ± 0.18 | 0.90 |
| IH-ML | 0.70 ± 0.16 | 0.66 ± 0.15 | 0.48 |
| IH-VT | 0.73 ± 0.15 | 0.62 ± 0.15 | 0.06 |
| Gait speed (m/s) | 0.89 ± 0.13 | 0.84 ± 0.16 | 0.39 |

All values are shown as mean ± standard deviation.

[a] Significant difference between two groups, $p < 0.05$

IH = Index of harmonicity; IH-AP = Index of harmonicity in the anteroposterior direction; IH-ML = Index of harmonicity in the mediolateral direction; IH-VT = Index of harmonicity in the vertical direction

# Discussion

The purpose of this study was to explore the potential for motor stress tests in improving clinical outcomes by detecting subtle gait impairment in older adults with MCI. Our findings demonstrated that the MCI group showed a decrease in gait smoothness in the mediolateral direction while walking with horizontal head turns compared to the peer group. However, the smoothness of the gait between the two groups did not differ under the constrained walking tasks (i.e., walking a narrow path and around an obstacle). To our knowledge, this study is possibly the first to investigate gait smoothness in older adults with MCI during complex motor tasks necessitating significant postural stability.

Freely turning the head during walking to allow a visual scan is a common movement essential for carrying out daily activities such as checking for oncoming traffic when crossing a street or navigating around obstacles [14]. During walking, the gravity sensors of the vestibular system, which are calibrated by the visual input, are responsible for gaze stabilization when the head or surrounding environment moves [44]. Previous studies demonstrated that head turning during walking, specifically in a horizontal direction, disturbed the dynamic balance control in older adults, both with and without pathological problems [45, 46]. In addition, Singh et al. [47] reported that the horizontal head-turn walking task adversely affected gait performance in older persons with poor lateral balance compared to the head-forward walking task, suggesting that this walking condition requires a high demand on mediolateral balance control. Therefore, walking in concurrent with horizontal head-turn may pose older adults with compromised mediolateral balance control at risk of falls. Moreover, a decline in gait smoothness, particularly in the lateral direction, has been evidenced in older adults with a fear of falling. Our findings extend these findings by suggesting that horizontal head turning induces motor stress on the balance control

system, leading to gait instability in older adults with MCI which is reflected through a reduction in mediolateral step-to-step symmetry. It might be possible that older adults with MCI have a decline in hippocampus formation, which is critical for an individual's spatial orientation and navigation via the vestibular-hippocampal pathway. In particular, head direction cells, which are a type of hippocampal neuron, utilize vestibular input to encode heading direction during movement; thereby, an impaired hippocampal function may influence the accuracy of current spatial location and directional heading in space [48]. In line with this postulation, previous studies found degeneration of the hippocampus and entorhinal cortex in the early stage of Alzheimer's disease, including MCI [49, 50]. Therefore, reduced gait smoothness in mediolateral direction observed in older adults with MCI might indicate diminished postural balance control when faced with walking conditions that affect visual and vestibular information such as horizontal head turns during walking. However, a decrease in gait smoothness in the MCI group under the vertical head-turn condition only reached a marginally significance (p = 0.06). To date, no studies have contrasted the difference in control of the gait between horizontal and vertical head-turn walking in older adults with and without MCI. A previous study exhibited a strong correlation between gaze stability performance during vertical head movement and gait performance in patients with vestibular disorders [51, 52]. These findings suggested that gaze stability performance, which is mediated via the vestibulo-ocular reflex, is prominent for successful walking during vertical head movement. Further research is warranted to examine this issue in older adults with MCI.

Regarding the constrained walking tasks, including walking a narrow path and around an obstacle, our findings exhibited that gait smoothness between older adults with and without MCI was similar, contrary to our hypothesis. However, for the narrow walking condition, the observed IH value in the vertical direction of both groups appeared to be lower than that in other directions. This may be due to the peculiar nature of narrow-path walking, which has unique dynamic gait

control characteristics, particularly in the vertical direction. Studies examining gait smoothness via IH value among persons with cognitive limitations, including MCI, while encountering physically constrained walking tasks are limited, making it difficult to compare the findings. To the best of our knowledge, only one previously published study by Pau et al. [26] reported that individuals with early and advanced cognitive decline had reduced gait symmetry expressed as the harmonic ratio, one of the metrics of gait smoothness, in the vertical and anteroposterior directions compared to healthy controls when walking at a normal pace on an unconstrained walking path. Regarding walking around an obstacle, the results showed that the gait speed was markedly slower than all other walking tasks. A prior study found that gait speed influenced the smoothness of gait, with slow gait speeds affecting smoothness more than comfortable and fast speeds [19], as also evidenced in our findings. We postulated that a decreased gait speed while navigating obstacles might be due to older adults adopting a more cautious gait pattern to reduce the magnitudes of the trunk accelerations to ensure walking stability; however, although older people attempt to attenuate gait speed, this does not maintain gait stability (reflecting via a lower IH in all directions), which is in line with previous studies [28, 53]. The continuous walking around the obstacles would be one of the motor stress tests that compromise gait stability in older adults with and without MCI.

There are certain limitations in this study that need to be addressed. Participants in the present study were healthy and well-functioning older adults. Thus, our findings may not be generalized to older adults with underlying physical conditions relevant to mobility problems. Moreover, we restricted the use of gait smoothness as an outcome measure due to the fact that it indicates a quality of rhythmic gait regulation; thereby, further studies that include other gait variables such as spatiotemporal, variability, and kinematics measures may provide insight into the whole picture of gait control in older adults with MCI during complex motor walking tasks.

# Conclusions

The present study provides evidence that older adults with MCI have compromised mediolateral gait stability during horizontal head-turning while walking. Measurement of gait smoothness under this walking condition may allow early detection of subtle changes in gait control and fall risk in older adults with MCI.

# Acknowledgements

The authors thank Poosri N., Kumfu S., and Lonlue N. for their assistance throughout all data collection. We would also like to thank physical therapists and staff in the subdistrict administrative organization and the elderly school for their contributions to participant recruitment. Finally, we would like to thank all the participants for their time and willingness to participate.

# References

1. Yogev- Seligmann G, Hausdorff JM, Giladi N. The role of executive function and attention in gait. Mov Disord. 2008;23(3):329-42. doi: 10.1002/mds.21720
2. van Iersel MB, Kessels RP, Bloem BR, Verbeek AL, Olde Rikkert MG. Executive functions are associated with gait and balance in community-living elderly people. J Gerontol A Biol Sci Med Sci. 2008;63(12):1344-9. doi: 10.1093/gerona/63.12.1344
3. Isordia Martínez J, Góngora Rivera F, Leal Bailey H, Ortiz Jiménez X. Mild cognitive impairment. Medicina. 2014;16(62):28-36.
4. Maquet D, Lekeu F, Warzee E, Gillain S, Wojtasik V, Salmon E, et al. Gait analysis in elderly adult patients with mild cognitive impairment and patients with mild Alzheimer's

disease: simple versus dual task: a preliminary report. Clin Physiol Funct Imaging. 2010;30(1):51-6. doi: 10.1111/j.1475-097X.2009.00903.x

5. Saunders NL, Summers MJ. Longitudinal deficits to attention, executive, and working memory in subtypes of mild cognitive impairment. Neuropsychology. 2011;25(2):237-48. doi: 10.1037/a0021134

6. Black SA, Rush RD. Cognitive and functional decline in adults aged 75 and older. J Am Geriatr Soc. 2002;50(12):1978-86. doi: 10.1046/j.1532-5415.2002.50609.x

7. Herman T, Mirelman A, Giladi N, Schweiger A, Hausdorff JM. Executive control deficits as a prodrome to falls in healthy older adults: a prospective study linking thinking, walking, and falling. J Gerontol A Biol Sci Med Sci. 2010;65(10):1086-92. doi: 10.1093/gerona/glq077

8. Verghese J, Robbins M, Holtzer R, Zimmerman M, Wang C, Xue X, et al. Gait dysfunction in mild cognitive impairment syndromes. J Am Geriatr Soc. 2008;56(7):1244-51. doi: 10.1111/j.1532-5415.2008.01758.x

9. Bahureksa L, Najafi B, Saleh A, Sabbagh M, Coon D, Mohler MJ, et al. The impact of mild cognitive impairment on gait and balance: a systematic review and meta-analysis of studies using instrumented assessment. Gerontology. 2017;63(1):67-83. doi: 10.1159/000445831

10. Beauchet O, Allali G, Launay C, Herrmann F, Annweiler C. Gait variability at fast-pace walking speed: a biomarker of mild cognitive impairment? J Nutr Health Aging. 2013;17(3):235-9. doi: 10.1007/s12603-012-0394-4

11. Boripuntakul S, Lord SR, Brodie M, Smith ST, Methapatara P, Wongpakaran N, et al. Spatial variability during gait initiation while dual tasking is increased in individuals with mild cognitive impairment. J Nutr Health Aging. 2014;18(3):307-12. doi: 10.1007/s12603-013-0390-3

12. Boripuntakul S, Lord SR, Methapatara P, Sungkarat S. Unplanned stopping strategy in individuals with mild cognitive impairment. Walailak J Sci Technol. 2017;14(1):35-42.

13. Boripuntakul S, Kamnardsiri T, Lord SR, Maiarin S, Worakul P, Sungkarat S. Gait variability during abrupt slow and fast speed transitions in older adults with mild cognitive impairment. PLoS One. 2022;17(10):e0276658. doi: 10.1371/journal.pone.0276658

14. Cinelli M, Patla A, Stuart B. Involvement of the head and trunk during gaze reorientation during standing and treadmill walking. Exp Brain Res. 2007;181(1):183-91. doi: 10.1007/s00221-007-0914-3

15. Cinelli M, Patla A, Stuart B. Age-related differences during a gaze reorientation task while standing or walking on a treadmill. Exp Brain Res. 2008;185(1):157-64. doi: 10.1007/s00221-007-1266-8

16. Leandri M, Cammisuli S, Cammarata S, Baratto L, Campbell J, Simonini M, et al. Balance features in Alzheimer's disease and amnestic mild cognitive impairment. J Alzheimer's Dis. 2009;16(1):113-20. doi: 10.3233/JAD-2009-0928

17. Brach JS, Perera S, VanSwearingen JM, Hile ES, Wert DM, Studenski SA. Challenging gait conditions predict 1-year decline in gait speed in older adults with apparently normal gait. Phys Ther. 2011;91(12):1857-64. doi: 10.2522/ptj.20100387

18. Shumway-Cook A, Patla AE, Stewart A, Ferrucci L, Ciol MA, Guralnik JM. Environmental demands associated with community mobility in older adults with and without mobility disabilities. Phys Ther. 2002;82(7):670-81.

19. Menz HB, Lord SR, Fitzpatrick RC. Acceleration patterns of the head and pelvis when walking on level and irregular surfaces. Gait Posture. 2003;18(1):35-46. doi: 10.1016/s0966-6362(02)00159-5

20. Lamoth C, Beek PJ, Meijer OG. Pelvis-thorax coordination in the transverse plane during gait. Gait Posture. 2002;16(2):101-14. doi: 10.1016/s0966-6362(01)00146-1

21. Lowry KA, Smiley- Oyen AL, Carrel AJ, Kerr JP. Walking stability using harmonic ratios in Parkinson's disease. Mov Disord. 2009;24(2):261-7. doi: 10.1002/mds.22352

22. Moe-Nilssen R. A new method for evaluating motor control in gait under real-life environmental conditions. part 2: gait analysis. Clin Biomech (Bristol, Avon). 1998;13(4-5):328-35. doi: 10.1016/s0268-0033(98)00090-4

23. Yack HJ, Berger RC. Dynamic stability in the elderly: identifying a possible measure. J Gerontol. 1993;48(5):M225-30. doi: 10.1093/geronj/48.5.m225

24. Menz HB, Lord SR, St George R, Fitzpatrick RC. Walking stability and sensorimotor function in older people with diabetic peripheral neuropathy. Arch Phys Med Rehabil. 2004;85(2):245-52. doi: 10.1016/j.apmr.2003.06.015

25. Kikkert LHJ, Vuillerme N, van Campen JP, Appels BA, Hortobágyi T, Lamoth CJC. Gait characteristics and their discriminative power in geriatric patients with and without cognitive impairment. J Neuroeng Rehabil. 2017;14(1):84. doi: 10.1186/s12984-017-0297-z

26. Pau M, Mulas I, Putzu V, Asoni G, Viale D, Mameli I, et al. Smoothness of gait in healthy and cognitively impaired individuals: a study on Italian elderly using wearable inertial sensor. Sensors (Basel). 2020;20(12):3577. doi: 10.3390/s20123577

27. Lamoth CJ, van Deudekom FJ, van Campen JP, Appels BA, de Vries OJ, Pijnappels M. Gait stability and variability measures show effects of impaired cognition and dual tasking in frail people. J Neuroeng Rehabil. 2011;8(1):1-9. doi: 10.1186/1743-0003-8-2

28. IJmker T, Lamoth CJ. Gait and cognition: the relationship between gait stability and variability with executive function in persons with and without dementia. Gait Posture. 2012;35(1):126-30. doi: 10.1016/j.gaitpost.2011.08.022

29. Marcos G, Santabárbara J, Lopez- Anton R, De- la- Cámara C, Gracia- García P, Lobo E, et al. Conversion to dementia in mild cognitive impairment diagnosed with DSM- 5 criteria and with Petersen's criteria. Acta Psychiatr Scand. 2016;133(5):378-85. doi: 10.1111/acps.12543

30. Kittipongpisal S. The accuracy study of the Thai word list learning test. J Ment Health Thai. 2015;23(2):91-101.

31. Boongird P. Mental state examination T10. Dementia Association of Thailand Newsletter. 2018(10).

32. Hughes CP, Berg L, Danziger WL, Coben LA, Martin RL. A new clinical scale for the staging of dementia. Br J Psychiatry. 1982;140:566-72. doi: 10.1192/bjp.140.6.566

33. Hemrungrojn S, Tangwongchai S, Charoenboon T, Panasawat M, Supasitthumrong T, Chaipresertsud P, et al. Use of the montreal cognitive assessment Thai version to discriminate amnestic mild cognitive impairment from Alzheimer's disease and healthy controls: machine learning results. Dement Geriatr Cogn Disord. 2021;50(2):183-94. doi: 10.1159/000517822

34. Wongpakaran N, Wongpakaran T, Van Reekum R. The use of GDS-15 in detecting MDD: a comparison between residents in a Thai long-term care home and geriatric outpatients. J Clin Med Res. 2013;5(2):101. doi: 10.4021/jocmr1239w

35. Walsh M, Connolly P, Jenkinson A, O'Brien T. Leg length discrepancy-an experimental study of compensatory changes in three dimensions using gait analysis. Gait Posture. 2000;12(2):156-61. doi: 10.1016/s0966-6362(00)00067-9

36. Azucena L. Digit span: effect of education and culture. Int J Psychol. 2006;41:333-41. doi: 10.1080/00207590500345724

37. Kortte KB, Horner MD, Windham WK. The Trail Making Test, part B: cognitive flexibility or ability to maintain set? Appl Neuropsychol. 2002;9(2):106-9. doi: 10.1207/S15324826AN0902_5

38. Huijben B, Van Schooten K, Van Dieën J, Pijnappels M. The effect of walking speed on quality of gait in older adults. Gait Posture. 2018;65:112-6. doi: 10.1016/j.gaitpost.2018.07.004

39. van Schooten KS, Pijnappels M, Rispens SM, Elders PJ, Lips P, Daffertshofer A, et al. Daily-life gait quality as predictor of falls in older people: a 1-year prospective cohort study. PLoS One. 2016;11(7):e0158623. doi: 10.1371/journal.pone.0158623

40. Micó-Amigo ME, Kingma I, Ainsworth E, Walgaard S, Niessen M, van Lummel RC, et al. A novel accelerometry-based algorithm for the detection of step durations over short episodes of gait in healthy elderly. J Neuroeng Rehabil. 2016;13(1):1-12. doi: 10.1186/s12984-016-0145-6

41. Gimmon Y, Barash A, Debi R, Snir Y, David YB, Grinshpon J, et al. Application of the clinical version of the narrow path walking test to identify elderly fallers. Arch Gerontol Geriatr. 2016;63:108-13. doi: 10.1016/j.archger.2015.10.010

42. Means KM. The obstacle course: a tool for the assessment of functional balance and mobility in the elderly. J Rehabil Res Dev. 1996;33:413-28.

43. Nakamura T, Kikumoto T, Hoshi F. Proactive postural control of the turning while walking in healthy adults. Phys Ther. 2015;101:e1072-3. doi: 10.1016/j.physio.2015.03.1959

44. Schubert MC, Minor LB. Vestibulo-ocular physiology underlying vestibular hypofunction. Phys Ther. 2004;84:373-5.

45. Marchetti GF, Whitney SL, Blatt PJ, Morris LO, Vance JM. Temporal and spatial characteristics of gait during performance of the Dynamic Gait Index in people with and people without balance or vestibular disorders. Phys Ther. 2008;88(5):640-51. doi: 10.2522/ptj.20070130

46. D'Silva L, Chalise P, Rippee M, Devos H. Challenging the vestibular system affects gait speed and cognitive workload in chronic mild traumatic brain injury and healthy adults. Front Neurol. 2022;13:819169. doi: 10.3389/fneur.2022.819169

47. Singh H, Sanders O, McCombe Waller S, Bair WN, Beamer B, Creath RA, et al. Relationship between head-turn gait speed and lateral balance function in community-dwelling older adults. Arch Phys Med Rehabil. 2017;98(10):1955-61. doi: 10.1016/j.apmr.2017.05.006

48. Stackman RW, Clark AS, Taube JS. Hippocampal spatial representations require vestibular input. Hippocampus. 2002;12(3):291-303. doi: 10.1002/hipo.1112

49. Anderson ND. State of the science on mild cognitive impairment (MCI). CNS Spectr. 2019; 24:78-87. doi: 10.1017/S1092852918001347

50. Du AT, Schuff N, Amend D, Laakso MP, Hsu YY, Jagust WJ, et al. Magnetic resonance imaging of the entorhinal cortex and hippocampus in mild cognitive impairment and Alzheimer's disease. J Neurol Neurosurg Psychiatry. 2001;71(4):441-7. doi: 10.1136/jnnp.71.4.441

51. Whitney SL, Marchetti GF, Pritcher M, Furman JM. Gaze stabilization and gait performance in vestibular dysfunction. Gait Posture. 2009;29(2):194-8. doi: 10.1016/j.gaitpost.2008.08.002

52. Whitney SL, Marchetti GF, Schade A, Wrisley DM. The sensitivity and specificity of the Timed “Up & Go" and the Dynamic Gait Index for self-reported falls in persons with vestibular disorders. J Vestib Res. 2004;14(5):397-409.

53. Menz HB, Lord SR, Fitzpatrick RC. Acceleration patterns of the head and pelvis when walking are associated with risk of falling in community-dwelling older people. J Gerontol A Biol Sci Med Sci. 2003;58(5):M446-M52. doi: 10.1093/gerona/58.5.m446

# Supporting information

**S1 File. Gait smoothness while walking over two types of complex motor walking tasks in the MCI and control groups.** Mean, standard deviation, maximum, and minimum values of index of harmonicity of AP, ML, and VT directions for each individual under complex motor walking tasks: I constrained walking tasks (i.e., walking a narrow path, walking around an obstacle) and II head turning while walking tasks (i.e., horizontal head turns, vertical head turns). (PDF)

Figure1

Click here to access/download;Figure;Fig1.tif

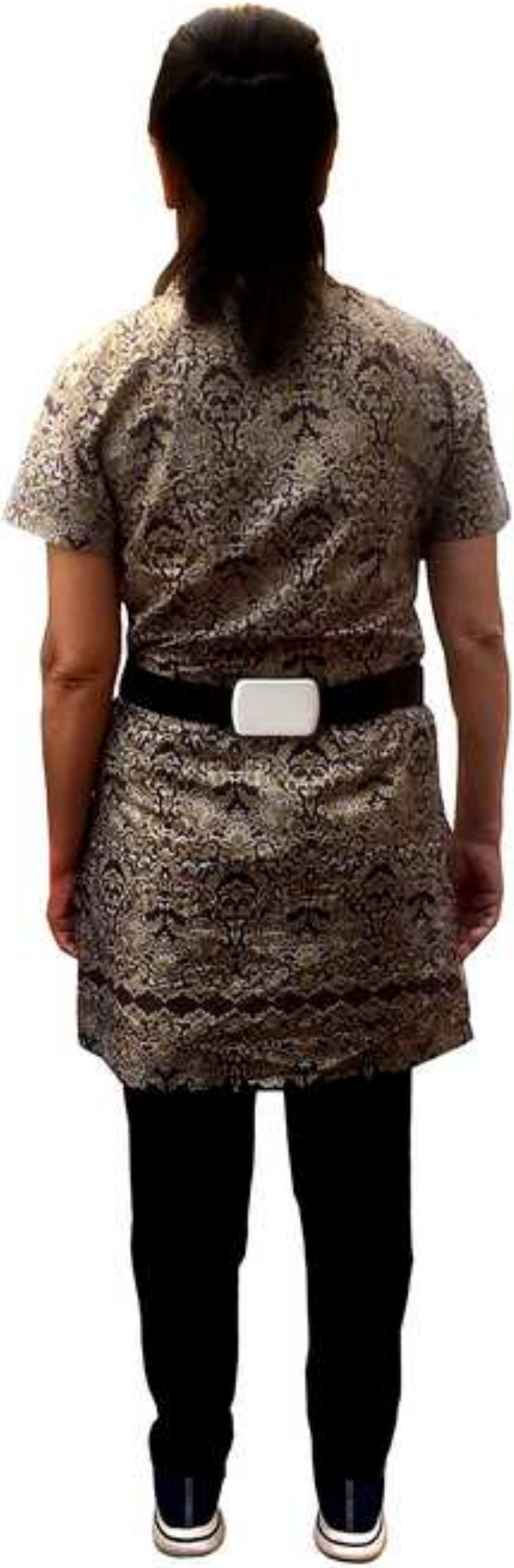

Figure2 Click here to access/download;Figure;Fig2.tif

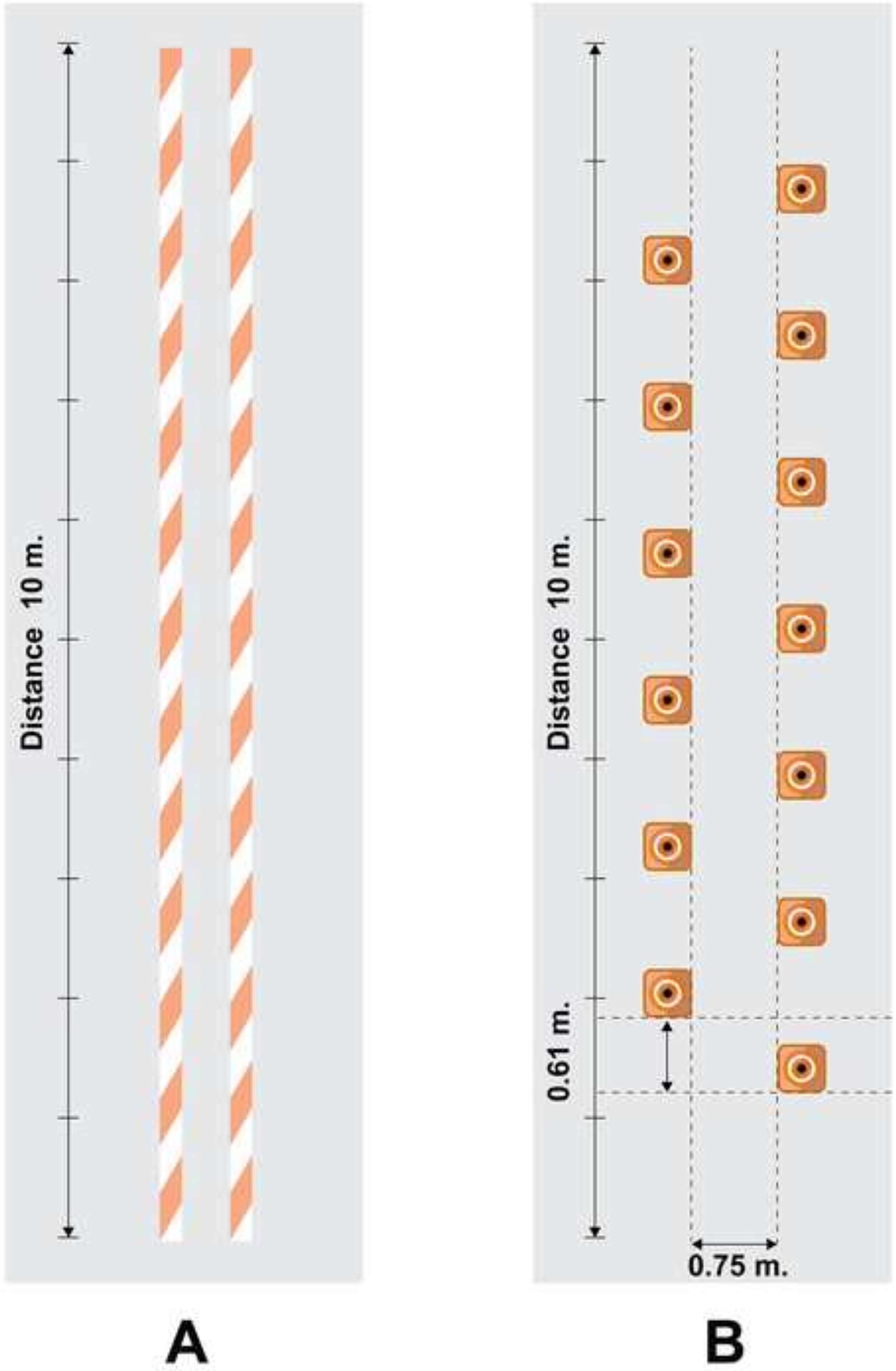

Figure3

Click here to access/download;Figure;Fig3.tif

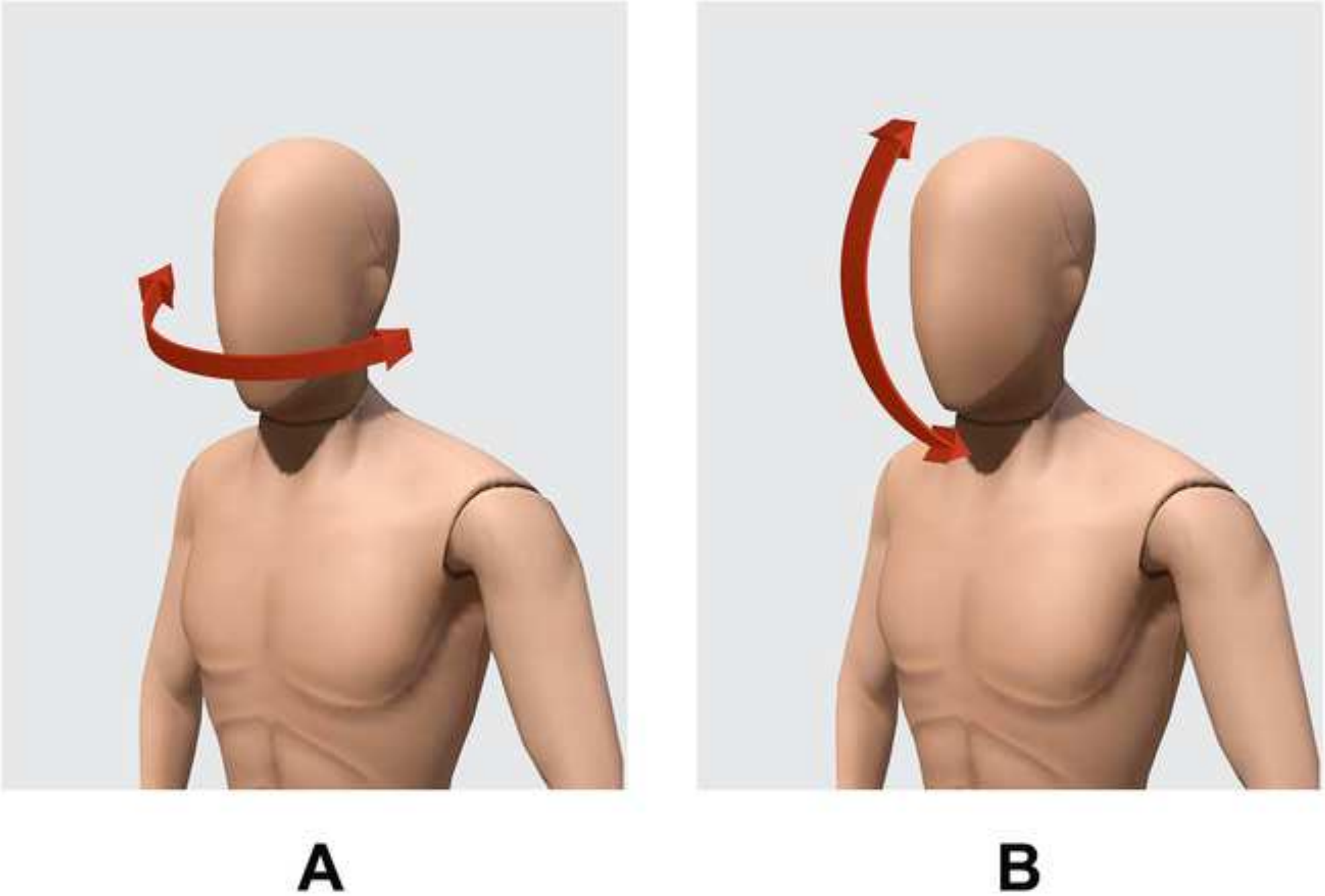

Click here to access/download
**Supporting Information**
S1_File.pdf
