# Supplementary material for: Gait smoothness during high-demand motor walking tasks in older adults with mild cognitive impairment
Source: PLoS One. 2024 Jan 19;19(1):e0296710. doi: 10.1371/journal.pone.0296710 (PMC10798528; doi:10.1371/journal.pone.0296710)
Supplement: Attachment — Submitted filename: PONE-D-23-25030_R1_reviewer_reviewer comments.pdf [file pone.0296710.s004.pdf]

PLOS ONE

Gait smoothness during high-demand motor walking tasks in older adults with mild cognitive impairment

--Manuscript Draft--

Manuscript Number:	PONE-D-23-25030R1
Article Type:	Research Article
Full Title:	Gait smoothness during high-demand motor walking tasks in older adults with mild cognitive impairment
Short Title:	Gait smoothness during high-demand motor walking tasks in MCI
Corresponding Author:	Sirinun Boripuntakul, Ph.D.<br>Chiang Mai University<br>Chiang Mai District, THAILAND
Keywords:	gait smoothness; mild cognitive impairment; motor load tasks; complex walking task; head turning while walking; index of harmonicity
Abstract:	Early signs of Mild Cognitive Impairment (MCI)-related gait deficits may be detected through the performance of complex walking tasks that require high gait control. Gait smoothness is a robust metric of overall body stability during walking. This study aimed to explore gait smoothness during complex walking tasks in older adults with and without MCI. Participants were 18 older adults with MCI (mean age = 67.89 ± 4.64 years) and 18 cognitively intact controls (mean age = 67.72 ± 4.63 years). Gait assessment was conducted under four complex walking tasks: walking a narrow path, walking around an obstacle, horizontal head turns while walking, and vertical head turns while walking. The index of harmonicity (IH), representing gait smoothness associated with overall body stability, was measured in anteroposterior, mediolateral, and vertical directions. A multivariate analysis was employed to compare the differences in IH between groups for each complex walking task. The MCI group demonstrated a reduction of IH in the mediolateral direction during the horizontal head turns than the control group (MCI group = 0.64 ± 0.16, Control group = 0.74 ± 0.12, $p = 0.04$). No significant differences between groups were found for the IH in other directions or walking conditions. These preliminary findings indicate that older adults with MCI have a decline in step regularity in the mediolateral direction during walking with horizontal head turns. Assessment of the smoothness of walking during head turns may be a useful approach to identifying subtle gait alterations in older adults with MCI, which may facilitate timely gait intervention.
Order of Authors:	Thanpidcha Poosri, Bachelors
	Sirinun Boripuntakul, Ph.D.
	Somporn Sungkarat, Ph.D.
	Teerawat Kamnardsiri, Ph.D.
	Atiwat Soontornpun, M.D.
	Kanokporn Pinyopornpanish, M.D.
Response to Reviewers:	Response to Reviewers' Comments<br><br>Journal Requirements:<br>When submitting your revision, we need you to address these additional requirements.<br><br>Comment # 1. Please ensure that your manuscript meets PLOS ONE's style requirements, including those for file naming. The PLOS ONE style templates can be found at<br>https://journals.plos.org/plosone/s/file?id=wjVg/PLOSOne_formatting_sample_main_body.pdf and<br>https://journals.plos.org/plosone/s/file?id=ba62/PLOSOne_formatting_sample_title_authors_affiliations.pdf<br>Response # 1. We have formatted our manuscript according to PLOS ONE's style

Powered by Editorial Manager® and ProduXion Manager® from Aries Systems Corporation

requirements.

Comment # 2. Please describe in your methods section how capacity to provide consent was determined for the participants in this study. Please also state whether your ethics committee or IRB approved this consent procedure. If you did not assess capacity to consent please briefly outline why this was not necessary in this case.
Response # 2. We have included this information in the methods section as follows (page 7, line 150-155):

"In this study, we assessed participants' decision-making capacity by evaluating their comprehension of the research study, including its purpose, procedures, risks, and benefits. Additionally, we appraised their capability to make a rational decision regarding participation in the study. All participants provided written informed consent prior to enrolling in the study. The institution's ethical review committee for research in humans approved this consent procedure (approval number: AMSEC-65EX-025)."

Reviewers' comments:
Reviewer's Responses to Questions

Reviewer # 1:
This quasi-experimental study was well-conceived, and the manuscript is well-organized and written. I made a few comments on word choice and have several questions/need for additional information (see comments made directly on the .pdf).
Response: Thank you very much.
We have revised the words and sentences throughout the manuscript according to the reviewer's suggestion as follows:
Page 3, line 50: We have revised "resulting" to "result".
Page 6, line 118: We have revised "walking while the vertical head turns" to "walking while making vertical head turns".
Page 7, line 142: We have revised "safety" to "safely".
Page 9, line 192 and 196: We have revised "walking in concurrent with horizontal/vertical head turns" to "walking straight with horizontal/vertical head turns".
Page 15, line 295: We have revised "in mediolateral direction" to "in the mediolateral direction".
Page 16, line 311: We have revised "high" to "higher".
Page 16, line 315: We have revised "a marginal" to "marginal".
Page 17, line 335: We have revised "the narrow walking" to "the narrow path walking".

Comment # 1. Please provide references attesting to the accuracy (validity) and precision (reliability) of this device.
Response # 1. We have included the references about the validity and reliability of the triaxial accelerometer device (DynaPort MoveMonitor, McRoberts) in the methods section (page 8, line 168).

Comment # 2. Line 166: please spell this abbreviation out and put IH in parentheses.
Response # 2. We spelled out the full name and abbreviation of IH on page 4, line 88. After that, we use the "IH" throughout the manuscript to keep the format consistent.

Comment # 3. Please describe any calibration procedure that might be used for this device prior to data collection.
Response # 3. The triaxial accelerometer device (DynaPort MoveMonitor, McRoberts) used in the present study has been calibrated prior to being dispatched (1). We followed the standardized procedures from previous studies to ensure the accuracy of gait measurement (1-2).
We have included the information in the methods section as follows (page 8, line 168-174):

"Following the standard protocol for accelerometer placement, the accelerometer was mounted at the middle of participants' fifth lumbar spine level and fastened with the supplied elastic belt [40] (Fig 1). In addition, The McRoberts logo on the device was placed outside; with this, the axes x, y, and z were automatically aligned with the direction of gravity. This placement allowed a dependable fixation of the accelerometer and supported the computation of trunk signal detection algorithm [41-42]"

Powered by Editorial Manager® and ProduXion Manager® from Aries Systems Corporation

References:
1.McRoberts [Internet]. Netherlands: McRoberts BV; 2023 [updated 2023; cited 2023 Dec 1]. Available from: https://www. https://www.mcroberts.nl/
2.van Hees V, Slootmaker S, Groot G, Mechelen W, Van Lummel R. Reproducibility of a triaxial seismic accelerometer (DynaPort). Med Sci Sports Exerc. 2009;41:810-7. doi: 10.1249/MSS.0b013e31818ff636
3.Mazzà C, Alcock L, Aminian K, Becker C, Bertuletti S, Bonci T, et al. Technical validation of real-world monitoring of gait: a multicentric observational study. BMJ Open. 2021;11(12):e050785. doi: 10.1136/bmjopen-2021-050785.

Comment # 4. Line 217: should be "IH is..."
Response # 4. We have rechecked and it should be “Po is….”. We aimed to define the formula for IH calculation.

Comment # 5. Line 219: I assume that this measure is unitless. If so, please declare this or provide the units of the measure.
Response # 5. Yes, it is unitless. We have mentioned this in the text (page 10, line 219-220).

Comment # 6. Line 222: suggest: "antero-posterior (AP), medio-lateral (ML), and vertical (VT)
Response # 6. We spelled out the full name and abbreviation of anteroposterior, mediolateral, and vertical on page 8, line 167-168. After that, we use the “AP”, “ML”, and “VT” throughout the manuscript to keep the format consistent.

Comment # 7. Please define P and Po in the part of gait smoothness definition.
Response # 7. We have defined P and Po (page 10, line 217-219).

Comment # 8. In the statistical analysis, should a correction be made with multiple t-tests, e.g., Bonferonni?
Response # 8. As this is an exploratory analysis, no adjustment was made for multiple comparisons of outcomes. However, as the dependent variables (i.e., IH in the AP, ML, and VT directions) of each walking task are related, the statistician suggested that a multivariate analysis should be conducted instead of an independent t-test. The results are still the same, wherein the MCI group had a significantly lower IH in the ML direction compared to the control group under horizontal head turns while walking ($p = 0.04$).
We have revised the statistical analysis, and acknowledged in the study limitation that findings are preliminary.

Comment # 9. In the discussion part, prior to restating the study purpose please briefly state the rationale/need for the study.
Response # 9. We have added a brief explanation of the rationale in the discussion part as follows (page 15, line 285-290):

“Complex walking tasks that require a high demand of gait control may be used to identify early gait impairments in individuals with MCI. The IH, as a measure of gait smoothness, serves as a robust metric for the rhythmic intersegmental coordination of trunk and pelvis movements within a stride, contributing to gait stability. Currently, there are limited studies that investigate gait smoothness in people with MCI during challenging walking tasks relevant to real-life walking situations.”

Comment # 10. Line 317-319: be careful - correlation is not cause/effect. You should report the precise correlation value and let the reader decide magnitude because I have seen r values of 0.3 which were statistically significant and might be called strong, when surely this number may not be clinically relevant, i.e., a $r = 0.3$ is an $r2 = 0.09$ which is relatively meaningless.
Response # 10. Thank you for pointing this out. We have revised this sentence as the reviewer’s suggestion (page 16, line 319-321).

Reviewer # 2:
Summary: The authors investigated gait smoothness in adults with and without mild cognitive impairment (MCI) during complex tasks that are common in daily life. The

authors quantified gait smoothness during walking as the Index of Harmonicity (IH) of a lumbar-mounted accelerometer. The IH of adults with and without MCI were compared across a narrow path task, an obstacle avoidance task, and a head turning task. The authors found moderate differences only in the mediolateral IH during the heading turning task, concluding that the IH during a head turning task may provide a valuable outcome to be used as early fall risk detection in people with MCI.

The results in this study have not been published elsewhere, to my knowledge. The manuscript is intelligible and with only minor typos, though focusing the writing on a specific target impact would improve the manuscript's clarity.

The authors provide strong mechanistic and empirical support for their premise: that experimental paradigms challenging motor and vestibular function would reveal subtle gait deficits in adults with (vs. without) MCI. However, the limited support for using the IH as a measure of fall risk and the limitations of the experimental methodology do not inspire confidence in the robustness of the primary outcomes or the knowledge gained from the study. Combined with limitations in the statistical analysis, these limitations provide poor support for the manuscript's conclusions. I do not think that the study's conclusions can be supported without significant additional data collection and revised analyses. Therefore, I do not recommend the manuscript for publication. I have provided specific comments below.

Comment # 1. The focus of the manuscript should be made more precise. The focus and implications seem to alternate between early fall risk detection, improving clinical outcomes, and characterizing stability deficits in adults with MCI.
Response # 1. We thank the reviewer for the thorough assessment and valuable comments on the manuscript. We apologize for the confusion caused by the writing that appears to alternate the study's focus back and forth. The focus of this study was to identify gait stability deficits in older adults with MCI. Specifically, we aimed to explore subtle MCI-related changes in gait under a high-demand motor walking task by using gait smoothness as a measurement indicator. Findings in this study might offer early detection of gait deficits and facilitate timely gait intervention for older adults with MCI. We have revised the introduction, objective, and conclusion based on this comment and the comment #2 (page 3, line 51-52; page 5, line 98-102; page 18, line 355-358).

Comment # 2. I am concerned by conclusions drawn from a single significant result amid many comparisons. Because multiple comparisons would support the same hypothesis-that gait smoothness differentially changes in adults with versus without MCI- the authors should correct for multiple comparisons. Given the single significant result ($p = 0.04$), it is likely that no comparisons would be significant after correction.
Response # 2. We appreciate the important points that the reviewer raises. This study aimed to explore which complex walking task(s) could differentiate gait changes between individuals with MCI and cognitively intact controls. As this is an exploratory analysis, no adjustment was made for multiple comparisons of outcomes. Each complex walking task (i.e., walking a narrow path, walking around an obstacle, horizontal head turns while walking, and vertical head turns while walking) was established to be a high-demand motor task that requires a unique and independent underlying mechanism of gait control. Further, as the dependent variables (i.e., IH in the AP, ML, and VT directions) of each walking task are related, the statistician suggested that a multivariate analysis should be conducted instead of an independent t-test. The results are still the same, wherein the MCI group had a significantly lower IH in the ML direction compared to the control group under horizontal head turns while walking ($p = 0.04$).

We have revised the introduction, statistical analysis, acknowledged in the study limitation that findings are preliminary and should be interpreted with caution, and moderated the conclusion accordingly.

Comment # 3. The authors should justify the use of a lumbar-worn accelerometer and use of linear accelerations to compute the Index of Harmonicity (IH). I am concerned that a lumbar sensor is a poor estimator of trunk accelerations. It is certainly less sensitive than (e.g.,) a sensor mounted at the thoracic spine. A lumbar sensor will not detect bending mid-torso.

Powered by Editorial Manager® and ProduXion Manager® from Aries Systems Corporation

Further, I question the author’s interpretation of a lumbar sensor as measuring trunk accelerations. A lumbar sensor is likely a better estimate of pelvis acceleration, but a poor measure of both pelvis and lumbar accelerations. This could readily be amended by revising the manuscript’s language.
As an aside, the oft-cited paper credited with first using the index of harmonicity (IH) used the pelvis and thoracic angles [1], though there does not appear to be strong support for one sensor placement versus another. Subsequent papers using a single lumbar sensor do not seem to evaluate the sensitivity of sensor configuration on the index of harmonicity.
[1] Lamoth CJ, Beek PJ, Meijer OG. Pelvis-thorax coordination in the transverse plane during gait. Gait & posture. 2002 Oct 1;16(2):101-14.
Response # 3. Previous studies have indicated that accelerometers can be placed either in the lower or upper trunk to measure trunk acceleration signals (1-4). In this study, we chose to mount an accelerometer at the lumbar because a systematic review has shown that trunk acceleration measured by a single triaxial accelerometer over the lower back level is a valid and reliable method for gait metrics analysis (4). Moreover, the single lumbar accelerometer used in the present study (DynaPort Hybrid, McRoberts, Netherlands) has also been valid and reliable for gait assessment across various populations (5-9). One explanation is that the lower back level approximates the body’s center of mass, which is the internal representation of the whole-body movement during walking (10). Thus, the selection of a single reference point on the lower trunk is based on the assumption that the task of maintaining stability when walking primarily requires controlling the motion of the center of mass. However, we agree with the reviewer that adding another sensor, such as at the upper trunk level, may provide more insight into the role and pattern of trunk movement control contributing to gait stability. We have included the above information in the study limitation as follows (Page 18, line 347-349):

“In addition to the triaxial lumbar accelerometer employed in the present study, adding another sensor at the thoracic level would provide a further understanding of how upper and lower intersegmental coordination movements contribute to gait stability.”
As the reviewer mentioned, a previous study by Lamoth et al. (11) did not strongly support one sensor placement over another. In their study, thoracic and pelvis axial rotations during walking were measured using standard method as light-emitting diodes (LEDs) and the Selspot™ Camera System (Selcom). Afterward, Lamoth et al. established the spectral analysis method for assessing the coordination of thoracic and pelvis movement within a stride using the IH, in which a power ratio of 1 indicates that the intersegmental movement is perfectly harmonic. These findings indicate that the IH can reflect rhythmic axial intersegmental coordination during walking, potentially contributing to overall gait stability.

Regarding the evidence of the sensitivity of sensor configuration on the IH involves using a single lumbar sensor. Unfortunately, there is currently no study assessing the sensitivity of the single body-fixed sensor for IH, as this gait metric is gaining attention and is still an ongoing area of research. Nevertheless, a previous study revealed that the single lumbar accelerometer was a valid, reliable, and sensitive instrument for measuring spatiotemporal gait parameters in older adults (12). In addition, a systematic review has reported that single-point lower back accelerometers were acceptable for assessing dynamic gait outcomes, including the IH (4).

References:
1.Doi T, Hirata S, Ono R, Tsutsumimoto K, Misu S, Ando H. The harmonic ratio of trunk acceleration predicts falling among older people: results of a 1-year prospective study. J Neuroeng Rehabil. 2013;28:10:7.
2.Terrier P, Reynard F. Effect of age on the variability and stability of gait: a cross-sectional treadmill study in healthy individuals between 20 and 69 years of age. Gait Posture. 2015;41:170-4.
3.Menz HB, Lord SR, Fitzpatrick RC. Acceleration patterns of the head and pelvis when walking are associated with risk of falling in community-dwelling older people. J Gerontol A Biol Sci Med Sci. 2003;58(5):M446-52.
4.Mobbs RJ, Perring J, Raj SM, Maharaj M, Yoong NKM, Sy LW, et al. Gait metrics analysis utilizing single-point inertial measurement units: a systematic review. Mhealth. 2022;8:9.

5.Houdijk H, Appelman FM, Van Velzen JM, van der Woude LH, van Bennekom CA. Validity of DynaPort GaitMonitor for assessment of spatiotemporal parameters in amputee gait. J Rehabil Res Dev. 2008;45:1335-42.
6.Bautmans I, Jansen B, Van Keymolen B, Mets T. Reliability and clinical correlates of 3D-accelerometry based gait analysis outcomes according to age and fall-risk. Gait Posture. 2011;33:366-72.
7.de Bruin ED, Hubli M, Hofer P, Wolf P, Murer K, Zijlstra W. Validity and reliability of accelerometer-based gait assessment in patients with diabetes on challenging surfaces. J Aging Res. 2012;2012:954378.
8.Hartmann A, Luzi S, Murer K, de Bie RA, de Bruin ED. Concurrent validity of a trunk tri-axial accelerometer system for gait analysis in older adults. Gait Posture. 2009;29:444-8.
9.Hartmann A, Murer K, de Bie RA, de Bruin ED. Reproducibility of spatio-temporal gait parameters under different conditions in older adults using a trunk tri-axial accelerometer system. Gait Posture. 2009;30:351-5.
10.Yang F, Pai YC. Can sacral marker approximate center of mass during gait and slip-fall recovery among community-dwelling older adults? J Biomech. 2014;47(16):3807-12.
11.Lamoth CJ, Beek PJ, Meijer OG. Pelvis-thorax coordination in the transverse plane during gait. Gait Posture. 2002;16(2):101-14.
12.Werner C, Heldmann P, Hummel S, Bauknecht L, Bauer JM, Hauer K. Concurrent validity, test-retest reliability, and sensitivity to change of a single body-fixed sensor for gait analysis during rollator-assisted walking in acute geriatric patients. Sensors (Basel). 2020;20(17):4866.

Comment # 4. The language should be more specific the manuscript. For example, gait stability is invoked as rationale for evaluating IH, but not strongly connected to IH of the pelvis/lumbar spine and is not discussed in the context of the results. Additional justification for why pelvis/lumbar smoothness represents gait stability would also improve the manuscript.

Further, the rationale for using the IH in the context of fall prevention is poor: the IH is not a reliable predictor of falls [1]. How, then, can the authors claim that the IH during a head turning task can be used for early fall detection without relating these results to fall risk?
[1] Riva F, Toebes MJ, Pijnappels MA, Stagni R, Van Dieën JH. Estimating fall risk with inertial sensors using gait stability measures that do not require step detection. Gait & posture. 2013 Jun 1;38(2):170-4.
Response # 4. Thank you for the suggestion. We have incorporated the connection between lumbar/pelvis movement, the IH, and gait stability into the rationale, as suggested by the reviewer. Furthermore, we have discussed these aspects in the context of the results within the discussion section (page 4-5, line 87-94; page 16, line 305-307).
We agree with the reviewer that the use of IH in the context of fall detection is poor. Therefore, we removed sentences about IH and early fall risk detection throughout the manuscript.

Comment # 5. The short walking trials used in this study are likely insufficient to extract stable measures of the IH [1]. Over one hundred strides are needed for the IH to approach a stable value in non-disabled adults. How can the authors trust their outcomes with only 20 m worth of strides of data per subject-trial? At a minimum, a bootstrapping procedure is needed to evaluate how cycle-to-cycle variations in gait and random sampling impact their results.
[1] Riva F, Bisi MC, Stagni R. Gait variability and stability measures: Minimum number of strides and within-session reliability. Computers in biology and medicine. 2014 Jul 1;50:9-13.
Response # 5. Several works have found that trunk acceleration-based measures are reliable for quantifying spatiotemporal gait parameters among healthy older adults and individuals with pathologic conditions (1-2); however, apart from Riva et al. (3), very limited studies investigate the reliability of trunk accelerometers on gait smoothness measures, including the IH. Moreover, no study has assessed cycle-to-cycle variations in the IH among older adults, both with and without cognitive limitations, especially under complex walking tasks.

Powered by Editorial Manager® and ProduXion Manager® from Aries Systems Corporation

As the reviewer's concern regarding the stable measures of the IH, we further explored the consistency of IH between the first and second trials for each walking condition in both the MCI and control groups, using test-retest reliability. The results showed good to excellent test-retest reliability of the IH for all walking conditions (as shown in Table 1).

Table 1 The intra-class correlation coefficient of the IH

Walking condition	Control group				MCI group			
	IH (Mean ± SD)		ICC 3,1 (95% CI)	p-value	IH (Mean ± SD)		ICC 3,1 (95% CI)	p-value
	1st trial	2nd trial			1st trial	2nd trial		
Walking forward (baseline)								
IH-AP	0.70 ± 0.08	0.67 ± 0.08	0.921	0.0001*	0.70 ± 0.12	0.67 ± 0.11	0.902	0.0001*
IH-ML	0.75 ± 0.09	0.75 ± 0.08	0.800	0.001*	0.75 ± 0.12	0.73 ± 0.12	0.908	0.0001*
IH-VT	0.86 ± 0.05	0.84 ± 0.07	0.913	0.0001*	0.85 ± 0.05	0.83 ± 0.05	0.898	0.0001*
Walking a narrow path								
IH-AP	0.76 ± 0.07	0.75 ± 0.08	0.855	0.0001*	0.74 ± 0.12	0.74 ± 0.11	0.928	0.0001*
IH-ML	0.77 ± 0.09	0.75 ± 0.10	0.820	0.0001*	0.74 ± 0.10	0.71 ± 0.12	0.879	0.0001*
IH-VT	0.22 ± 0.14	0.20 ± 0.14	0.930	0.0001*	0.24 ± 0.14	0.23 ± 0.14	0.960	0.0001*
Walking around an obstacle								
IH-AP	0.17 ± 0.10	0.20 ± 0.15	0.858	0.0001*	0.20 ± 0.15	0.20 ± 0.15	0.956	0.0001*
IH-ML	0.53 ± 0.15	0.52 ± 0.15	0.835	0.0001*	0.52 ± 0.17	0.49 ± 0.19	0.959	0.0001*
IH-VT	0.39 ± 0.20	0.34 ± 0.21	0.837	0.0001*	0.37 ± 0.19	0.35 ± 0.19	0.963	0.0001*
Horizontal head turns while walking								
IH-AP	0.38 ± 0.23	0.35 ± 0.24	0.961	0.0001*	0.38 ± 0.21	0.34 ± 0.19	0.761	0.003*
IH-ML	0.73 ± 0.12	0.74 ± 0.12	0.958	0.0001*	0.63 ± 0.16	0.64 ± 0.17	0.974	0.0001*
IH-VT	0.67 ± 0.18	0.70 ± 0.16	0.899	0.0001*	0.59 ± 0.19	0.62 ± 0.16	0.898	0.0001*
Vertical head turns while walking								
IH-AP	0.60 ± 0.19	0.62 ± 0.20	0.968	0.0001*	0.60 ± 0.19	0.60 ± 0.18	0.926	0.0001*
IH-ML	0.69 ± 0.16	0.71 ± 0.16	0.947	0.0001*	0.66 ± 0.15	0.66 ± 0.15	0.953	0.0001*
IH-VT	0.72 ± 0.18	0.73 ± 0.14	0.919	0.0001*	0.63 ± 0.16	0.62 ± 0.16	0.915	0.0001*

*p-value < 0.05
ICC = intra-class correlation coefficient
IH = Index of harmonicity; IH-AP = Index of harmonicity in the anteroposterior direction; IH-ML = Index of harmonicity in the mediolateral direction; IH-VT = Index of harmonicity in the vertical direction

The results from Table 1 indicate an acceptable reliability of the IH measures using trunk accelerometers obtained from short walking trials. Currently, several studies determine gait smoothness, including the IH and Harmonic Ratio (HR), in older adults and individuals with pathologic conditions within a short distance range from 8 to 30 meters (4-7). However, we agree with the reviewer that adding the number of strides by increasing the walking distance would allow for stable gait outcomes. We acknowledged that it is a limitation of the study and stated in the manuscript (page 18, line 349-350) as follows:

"Moreover, a long walking distance that covers more strides would allow for a steady gait outcome analysis."

References:

1.Kobsar D, Charlton JM, Tse CTF, Esculier JF, Graffos A, Krowchuk NM, et al. Validity and reliability of wearable inertial sensors in healthy adult walking: a systematic review and meta-analysis. J. Neuroeng Rehabil. 2020;17:62.
2.Werner C, Heldmann P, Hummel S, Bauknecht L, Bauer JM, Hauer K. Concurrent validity, test-retest reliability, and sensitivity to change of a single body-fixed sensor for gait analysis during rollator-assisted walking in acute geriatric patients. Sensors (Basel). 2020;20(17):4866.
3.Riva F, Bisi MC, Stagni R. Gait variability and stability measures: minimum number of strides and within-session reliability. Comput Biol Med. 2014;50:9-13.
4.Simonetti E, Pillet H, Vannozzi G, Loiret I, Villa C, Bascou J, et al. Investigating symmetry in amputee gait through the improved harmonic ratio: influence of the stride segmentation method. Comput Methods Biomech Biomed Engin. 2019;22(sup1):S221-S3.
5.Micó-Amigo ME, Kingma I, Heinzel S, Nussbaum S, Heger T, van Lummel RC, et al.

Powered by Editorial Manager® and ProduXion Manager® from Aries Systems Corporation

Dual vs. single tasking during circular walking: what better reflects progression in Parkinson's disease? Front Neurol. 2019;10:372.
6.Pau M, Mulas I, Putzu V, Asoni G, Viale D, Mameli I, et al. Smoothness of gait in healthy and cognitively impaired individuals: a study on Italian elderly using wearable inertial sensor. Sensors (Basel). 2020;20(12):3577.
7.Javadpour S, Sinaei E, Salehi R, Zahednejad S, Motealleh A. Comparing the effects of single-task versus dual-task balance training on gait smoothness and functional balance in community-dwelling older adults: a randomized controlled trial. J Aging Phys Act. 2022;30(2):308-15.

Comment # 6. The authors should specify if the trial order was randomized.
Response # 6. Thank you. The trial order was randomized. We have added this statement in the method section (page 10, line 206-207).

Comment # 7. The authors should consider how instructions for the head turning task may impact IH. Per the instructions, the rate of head turning would differ based on each participant's walking speed ("head turn every 1 meter" [sic]). As gaze stabilization performance is associated with walking performance in adults with vestibular dysfunction [1] and head turning alters gait stability [2], it is plausible that varying the rate of head rotation would differentially alter gait stability and/or smoothness in people with different walking speeds.
[1] Whitney SL, Marchetti GF, Pritcher M, Furman JM. Gaze stabilization and gait performance in vestibular dysfunction. Gait Posture. 2009 Feb;29(2):194-8. doi: 10.1016/j.gaitpost.2008.08.002. Epub 2008 Sep 23. PMID: 18815040; PMCID: PMC4879829.
[2] Fitzgerald C, Thomson D, Zebib A. et al. A comparison of gait stability between younger and older adults while head turning. Exp Brain Res. 238, 1871-1883 (2020). https://doi.org/10.1007/s00221-020-05846-3
Response # 7. Thank you for your point of view. We agree with the reviewer that the rate of head turning while walking relates to walking speed, which may result in gait stability. In the present study, we consistently gave a standard instruction for participants to turn their heads as fast and as far as possible in response to the command every 1 meter. With this, there was no discernible difference in the head turning rate between individuals or walking trials. As we found, all participants could follow the instructions and complete the walking tasks without obviously changing gait speed from baseline. Moreover, the results showed that gait speed in head turning conditions was similar between the MCI and control groups, suggesting that the difference in the IH between the two groups was not influenced by gait speed (Table 2 in the manuscript).

Comment # 8. I am surprised to see such small differences in Trails B-A scores between groups. In these tasks, I might expect set-shifting ability to correlate with changes in IH. The authors may see larger effects of head turning or obstacle negotiation if groups differed in clinical scores associated with set-shifting or motor-cognitive integration.
Response # 8. We agree with the reviewer that larger effects of head turning or obstacle negotiation might be observed if Trail B-A scores were differences between the two groups. The head turning or obstacle negotiation while walking serves as a motor dual-tasking as participants have to allocate their attention between walking and a head turning or obstacle negotiation task. Non-significant differences in TMT B-A scores in the present study are likely due to a large variation of the data.

Comment # 9. In general, the Discussion is not strongly focused on interpreting and contextualizing the study's findings. For example, paragraph 2 could be written almost entirely without conducting the study. Condensing the Discussion and improving focus on contextualizing results would improve the manuscript.
Response # 9. We appreciate your constructive feedback. We have re-written the discussion to focus on interpretating and contextualizing the study findings. Thank you once again for your valuable suggestion.

Reviewer # 3:
The paper provides valuable insights into the assessment of gait changes in older adults with Mild Cognitive Impairment (MCI) under complex motor tasks. Here is a review of the introduction and methodology sections:

	Comment # 1.<br>Introduction Review:<br>The introduction sets the stage effectively by discussing the importance of executive function, attention, and their connection to safe mobility. The rationale for the study, focusing on the potential for gait abnormalities in individuals with MCI, is well-explained. This establishes the significance of the research. The introduction highlights the existing research and points out discrepancies, which helps justify the need for the study. The introduction clearly defines terms like MCI, making it reader-friendly for those not well-versed in the subject matter. The study's objectives and hypotheses are clearly stated, providing a solid foundation for the research.<br>Response # 1. Thank you very much.<br><br>Comment # 2.<br>Methodology Review:<br>The methodology section adequately describes the participant selection process and sample size calculation, contributing to the paper's transparency. Inclusion and exclusion criteria are well-defined, ensuring that the study focuses on the intended population. Ethical considerations and approval from the institution's review committee are appropriately mentioned, underlining the ethical aspects of the research. The assessment of gait smoothness under complex motor tasks is well-explained, including the use of a triaxial accelerometer device, making it clear to the reader how data was collected. The statistical analysis section is detailed and provides a clear understanding of how the data was analyzed. The results are presented in a clear and organized manner, with tables that effectively summarize the findings.<br>Response # 2. Thank you very much.<br><br>Comment # 3. General Review:<br>The paper is generally well-structured and follows a logical flow, with each section building upon the previous one. The paper maintains a strong focus on the research question throughout the introduction and methodology sections. The language is technical but clear, making the content accessible to a broader audience. The limitations are acknowledged, and the need for further research is suggested, showing the author's awareness of the study's boundaries.<br><br>In summary, the introduction effectively introduces the research problem and its significance, while the methodology provides a clear framework for the study, including participant selection and data collection methods. The paper appears to be well-structured and technically sound, making it a valuable contribution to the field of research on gait abnormalities in older adults with MCI.<br>Response # 3. Thank you very much.
**Additional Information:**	
**Question**	**Response**
**Financial Disclosure**<br><br>Enter a financial disclosure statement that describes the sources of funding for the work included in this submission. Review the submission guidelines for detailed requirements. View published research articles from *PLOS ONE* for specific examples.<br><br>This statement is required for submission and **will appear in the published article** if the submission is accepted. Please make sure it is accurate.	-Initials of the authors who received each award: Thanpidcha Poosri<br>-Grant numbers awarded to each author: AMS-2564<br>-The full name of each funder: Faculty of Associated Medical Sciences, Chiang Mai University, Thailand.<br>-URL of each funder website: N/A<br>-Did the sponsors or funders play any role in the study design, data collection and analysis, decision to publish, or preparation of the manuscript?: The funders had no role in study design, data collection and analysis, decision to publish, or preparation of the manuscript.

**Unfunded studies**<br>Enter: *The author(s) received no specific funding for this work.*<br><br>**Funded studies**<br>Enter a statement with the following details:<br>• Initials of the authors who received each award<br>• Grant numbers awarded to each author<br>• The full name of each funder<br>• URL of each funder website<br>• Did the sponsors or funders play any role in the study design, data collection and analysis, decision to publish, or preparation of the manuscript?<br>• **NO** - Include this sentence at the end of your statement: *The funders had no role in study design, data collection and analysis, decision to publish, or preparation of the manuscript.*<br>• **YES** - Specify the role(s) played.<br><br>* typeset	
**Competing Interests**<br><br>Use the instructions below to enter a competing interest statement for this submission. On behalf of all authors, disclose any competing interests that could be perceived to bias this work—acknowledging all financial support and any other relevant financial or non-financial competing interests.<br><br>This statement is required for submission and **will appear in the published article** if the submission is accepted. Please make sure it is accurate and that any funding sources listed in your Funding Information later in the submission form are also declared in your Financial Disclosure statement.<br><br>View published research articles from *PLOS ONE* for specific examples.	The authors have declared that no competing interests exist.

Powered by Editorial Manager® and ProduXion Manager® from Aries Systems Corporation

**NO authors have competing interests**<br><br>Enter: *The authors have declared that no competing interests exist.*<br><br>**Authors with competing interests**<br><br>Enter competing interest details beginning with this statement:<br><br>*I have read the journal's policy and the authors of this manuscript have the following competing interests: [insert competing interests here]*<br><br>* typeset	
**Ethics Statement**<br><br>Enter an ethics statement for this submission. This statement is required if the study involved:<br><br>• Human participants<br>• Human specimens or tissue<br>• Vertebrate animals or cephalopods<br>• Vertebrate embryos or tissues<br>• Field research<br><br>Write "N/A" if the submission does not require an ethics statement.<br><br>General guidance is provided below. Consult the submission guidelines for detailed instructions. **Make sure that all information entered here is included in the Methods section of the manuscript.**	Human Subject Research:<br>-Human Research Ethics Unit, Faculty of Associated Medical Sciences, Chiang Mai University, Thailand<br>-Approval number: AMSEC-65EX-025<br>-Form of consent obtained: written

Powered by Editorial Manager® and ProduXion Manager® from Aries Systems Corporation

**Format for specific study types**<br><br>**Human Subject Research (involving human participants and/or tissue)**<br>• Give the name of the institutional review board or ethics committee that approved the study<br>• Include the approval number and/or a statement indicating approval of this research<br>• Indicate the form of consent obtained (written/oral) or the reason that consent was not obtained (e.g. the data were analyzed anonymously)<br><br>**Animal Research (involving vertebrate animals, embryos or tissues)**<br>• Provide the name of the Institutional Animal Care and Use Committee (IACUC) or other relevant ethics board that reviewed the study protocol, and indicate whether they approved this research or granted a formal waiver of ethical approval<br>• Include an approval number if one was obtained<br>• If the study involved *non-human primates*, add *additional details* about animal welfare and steps taken to ameliorate suffering<br>• If anesthesia, euthanasia, or any kind of animal sacrifice is part of the study, include briefly which substances and/or methods were applied<br><br>**Field Research**<br><br>Include the following details if this study involves the collection of plant, animal, or other materials from a natural setting:<br>• Field permit number<br>• Name of the institution or relevant body that granted permission	
**Data Availability**<br><br>Authors are required to make all data underlying the findings described fully available, without restriction, and from the time of publication. PLOS allows rare exceptions to address legal and ethical concerns. See the PLOS Data Policy and FAQ for detailed information.	Yes - all data are fully available without restriction

A Data Availability Statement describing where the data can be found is required at submission. Your answers to this question constitute the Data Availability Statement and **will be published in the article**, if accepted.<br><br>**Important:** Stating 'data available on request from the author' is not sufficient. If your data are only available upon request, select 'No' for the first question and explain your exceptional situation in the text box.<br><br>Do the authors confirm that all data underlying the findings described in their manuscript are fully available without restriction?	
**Describe where the data may be found in full sentences. If you are copying our sample text, replace any instances of XXX with the appropriate details.**<br><br>• If the data are **held or will be held in a public repository**, include URLs, accession numbers or DOIs. If this information will only be available after acceptance, indicate this by ticking the box below. For example: *All XXX files are available from the XXX database (accession number(s) XXX, XXX.*).<br>• If the data are all contained **within the manuscript and/or Supporting Information files**, enter the following: *All relevant data are within the manuscript and its Supporting Information files.*<br>• If neither of these applies but you are able to provide **details of access elsewhere**, with or without limitations, please do so. For example:<br><br>*Data cannot be shared publicly because of [XXX]. Data are available from the XXX Institutional Data Access / Ethics Committee (contact via XXX) for researchers who meet the criteria for access to confidential data.*<br><br>*The data underlying the results presented in the study are available from (include the name of the third party*	All relevant data are within the manuscript and its Supporting Information files.

and contact information or URL).<br>• This text is appropriate if the data are owned by a third party and authors do not have permission to share the data.<br><br>* typeset	
Additional data availability information:	Tick here if the URLs/accession numbers/DOIs will be available only after acceptance of the manuscript for publication so that we can ensure their inclusion before publication.

Powered by Editorial Manager® and ProduXion Manager® from Aries Systems Corporation

Revised Manuscript Clean Copy
Click here to access/download;Manuscript;Revised Manuscript.docx

Gait smoothness during high-demand motor walking tasks in older adults with mild cognitive impairment

Thanpidcha Poosri[1], Sirinun Boripuntakul[1*], Somporn Sungkarat[1], Teerawat Kamnardsiri[2], Atiwat Soontornpun[3], Kanokporn Pinyopornpanish[4]

[1] Department of Physical Therapy, Faculty of Associated Medical Sciences, Chiang Mai University, Thailand

[2] Department of Digital Game, College of Arts, Media and Technology, Chiang Mai University, Thailand

[3] Department of Internal Medicine, Faculty of Medicine, Chiang Mai University and Northern Neuroscience Center, Thailand

[4] Department of Family Medicine, Faculty of Medicine, Chiang Mai University, Thailand

* Corresponding author

E-mail: sirinun.b@cmu.ac.th (SB)

Abstract

Early signs of Mild Cognitive Impairment (MCI)-related gait deficits may be detected through the performance of complex walking tasks that require high gait control. Gait smoothness is a robust metric of overall body stability during walking. This study aimed to explore gait smoothness during complex walking tasks in older adults with and without MCI. Participants were 18 older adults with MCI (mean age = 67.89 ± 4.64 years) and 18 cognitively intact controls (mean age = 67.72 ± 4.63 years). Gait assessment was conducted under four complex walking tasks: walking a narrow path, walking around an obstacle, horizontal head turns while walking, and vertical head turns while walking. The index of harmonicity (IH), representing gait smoothness associated with overall body stability, was measured in anteroposterior, mediolateral, and vertical directions. A multivariate analysis was employed to compare the differences in IH between groups for each complex walking task. The MCI group demonstrated a reduction of IH in the mediolateral direction during the horizontal head turns than the control group (MCI group = 0.64 ± 0.16, Control group = 0.74 ± 0.12, $p = 0.04$). No significant differences between groups were found for the IH in other directions or walking conditions. These preliminary findings indicate that older adults with MCI have a decline in step regularity in the mediolateral direction during walking with horizontal head turns. Assessment of the smoothness of walking during head turns may be a useful approach to identifying subtle gait alterations in older adults with MCI, which may facilitate timely gait intervention.

Introduction

Higher level cognitive function, specifically executive function, and attention, plays a vital role in integrating sensorimotor information and controlling movement execution, contributing to safe mobility in complex real-life environments [1, 2]. Previous studies have found that individuals with Mild Cognitive Impairment (MCI), a clinical entity involving cognitive deficit beyond normal aging [3, 4], displayed a decline in attention and executive function [5] which may in turn result in gait abnormalities and increased risk of falling [6, 7]. Therefore, the early identification of subtle MCI-related changes in gait may have important clinical benefits, ensuring a timely assessment and intervention in gait-related concerns.

Existing studies have demonstrated that simple steady-state walking tasks allow the detection of gait changes related to falls in older adults with MCI compared to their cognitively intact peers [4, 8, 9]. While the above findings were promising, some studies failed to find differences in gait features between persons with MCI and those cognitively intact controls [4, 9, 10]. Discrepancies across prior findings may be due to the variations in gait tasks and outcome measures. It is posited that complex walking tasks could exacerbate subtle MCI-related gait impairment. Currently, it is well documented that gait impairment of individuals with MCI is significantly pronounced when adding a cognitive load while walking (dual-tasking) [9]. However, previous studies examining MCI-related changes in gait under motor load tasks relevant to a real-life walking situation are scarce. Limited studies have demonstrated a decline in gait regulation of older adults with MCI during transitional gait speeds, including gait initiation, termination, and slow speed transition [11-13]. These findings suggest that motor load tasks may be sensitive in detecting subtle gait changes in older adults with MCI.

Head turning is a fundamental component for successful responses to environmental demands via visual scanning, such as checking for traffic before crossing the road and navigating the barriers in the travel path [14]. Basically, head turning while walking alters visual and vestibular information from the changes of visual field. Specifically, when individuals need to adjust their gait in real-time while head-turning in response to environmental constraints, the vestibular system is more burdened with tracing head movements, which may compromise gait stability [15]. Previous studies have elicited a reduction in postural stability in individuals with MCI partly due to a decrement in the vestibular system linked to a decline in hippocampus function [16]. Therefore, it is plausible that head manipulation while walking could be considered a candidate measure of gait stability in older adults with MCI. Moreover, complex walking tasks concerning everyday environments that require high motor demands in older adults, including walking a narrow path and around an obstacle [17, 18], may challenge gait control in older adults with MCI. Taken together, high-demand motor walking tasks, including head turns while walking, walking a narrow path, and walking around an obstacle, may be used as a motor stress test to amplify a subtle change of gait in older adults with MCI.

The smoothness of walking, the rhythmic pattern of trunk acceleration, is a dynamic gait metric useful to quantify gait regularity during walking [19-21]. Prior works have demonstrated impaired gait smoothness across various populations, such as cognitively impaired individuals, older adults with restricted neuromuscular control, Parkinson’s disease patients, and individuals with sensory impairment, compared to young adults and persons without the disease [21-27]. According to recent studies, trunk accelerations are more sensitive than traditional spatiotemporal metrics for capturing subtle alterations in gait [26, 28]. Among gait smoothness measures, the index of harmonicity (IH) provides information on the harmonicity of trunk and pelvis movement in conjunction with gait rhythm through spectral analysis of trunk acceleration

signals [20]. Specifically, the IH indicates the smoothness of rhythmic intersegmental coordination between the trunk and pelvis throughout the gait cycle, providing insight into balance control during walking [21]. It is well established that dynamic balance and movement coordination during walking are prerequisites for gait stability. With this, the IH has been proposed as a marker indicating the smoothness of gait in relation to walking stability.

To date, gait smoothness has been used to examine gait quality in limited groups and walking conditions. In addition, to the best of our knowledge, no study has investigated the smoothness of gait in people with MCI under high-demand motor tasks relevant to real-life environmental conditions. The aim of this preliminary study was to explore subtle MCI-related changes in gait under a high-demand motor walking task by using gait smoothness as a measurement indicator. It was hypothesized that the MCI group would have less gait smoothness under motor load tasks than the control group. Findings in this study might offer early detection of gait deficits and facilitate timely gait intervention for older adults with MCI.

Material and methods

Participants

The sample size was calculated based on our pilot study, in which the smallest effect size was chosen. The smallest effect size of 0.22 was derived from the IH in the anteroposterior direction in the condition of walking while making vertical head turns (with baseline gait speed as a covariate). With this, a general linear model with an effect size of 0.22, a power of 0.80, and an alpha level of 0.05 revealed a total sample size of at least 26 participants (13 participants per group).

Eighteen community-dwelling older adults with MCI and 18 healthy-matched controls aged 60 or older were enrolled in the present study. The older adults with MCI were recruited from Maharaj Nakorn Chiang Mai Hospital, a tertiary care hospital in Northern Thailand. The diagnosis of MCI was performed by an experienced neurologist based on Petersen's criteria [29]: (i) subjective complaint of decline in memory on self-or informant report, (ii) isolated memory deficits (determined by the 10-word list learning test ≤ 5) [30], (iii) essentially preserved general cognitive function (determined by the Mental State Examination T10 (MSET10) > 22 or adjusted for educational level) [31], (iv) largely intact activities of daily living using the clinical review with the patients and informant interview, and (v) clinical dementia (determined by the global Clinical Dementia Rating (CDR) scale < 1) [32]. Additionally, all participants with MCI were required to score on the Montreal Cognitive Assessment (MoCA) ≤ 24 to affirm the presence of subtle cognitive impairment [33]. The cognitively intact control group with similar age (plus or minus two years), gender, body mass index (plus or minus two units), and education level (plus or minus two years) was recruited from Chiang Mai community via local community groups and social media advertisements

(e.g., Facebook). To be eligible, all participants in the control group were required to have intact cognitive function, as identified by the normal scores of the MSET10 and MoCA.

The inclusion criteria for both groups were being able to walk independently and safely without an assistive device for at least 10 meters and being able to comprehend and follow the instructions. The exclusion criteria were: (i) had major health conditions that affect walking ability or safely during testing such as musculoskeletal, neurological, cardiorespiratory problems or any unstable medical conditions (e.g., Parkinson's disease, stroke, peripheral vertigo, acute joint pain, uncorrected visual and/or hearing impairment), (ii) had severe dizziness when turning the head in horizontal and/or vertical directions, (iii) had depressive symptoms (determined by the Thai Geriatric Depression Scale-15 score > 6) [34], (iv) current use of sedative drugs, antipsychotic drugs, and narcotic drugs, and (v) had leg length discrepancy > 2.5 cm (assessed from the anterior superior iliac spine (ASIS) to the tip of medial malleolus in a standing position using a flexible tape measure) [35].

In this study, we assessed participants' decision-making capacity by evaluating their comprehension of the research study, including its purpose, procedures, risks, and benefits. Additionally, we appraised their capability to make a rational decision regarding participation in the study. All participants provided written informed consent prior to enrolling in the study. The institution's ethical review committee for research in humans approved this consent procedure (approval number: AMSEC-65EX-025). The start and end of the study were from the 26th of August 2022 to the 9th of April 2023. Before gait testing, participants in both groups were interviewed about personal demographic data, including age, weight, height, education level, fall incidence within the past year, medical conditions, and medication used. Additionally, participants underwent an assessment of attention and executive functions, which

were previously determined to be closely related to gait control, using the Digit Span Forward and Backward Tests and Trail Making Test Parts A and B, respectively [36, 37].

Assessment of gait smoothness under complex motor walking tasks

A triaxial accelerometer device (DynaPort MoveTest, McRoberts, The Hague, The Netherlands) with a sampling frequency of 100 Hz was used to measure the IH, which is an indicator of gait smoothness along three orthogonal axes, including anteroposterior (AP), mediolateral (ML), and vertical (VT) directions [38, 39]. Following the standard protocol for accelerometer placement, the accelerometer was mounted at the middle of participants' fifth lumbar spine level and fastened with the supplied elastic belt [40] (Fig 1). In addition, The McRoberts logo on the device was placed outside; with this, the axes x, y, and z were automatically aligned with the direction of gravity. This placement allowed a dependable fixation of the accelerometer and supported the computation of trunk signal detection algorithm [41-42]. The trunk acceleration signal during walking was transmitted in real-time via Bluetooth to a software database on the personal computer, where the signal-based parameters including IH were subsequently analyzed by an algorithm developed by Micó-Amigo et al. [43].

Fig 1. Placement of triaxial accelerometer on the lower back at the level of the fifth lumbar vertebra.

After a triaxial accelerometer setting, participants were requested to walk along a 10-meter walkway at their comfortable speed for two trials to use as baseline data. As for the gait testing protocol, participants were evaluated for gait smoothness under four complex motor walking tasks on the outdoor walking terrain as follows:

A. Walking a narrow path (WN): Participants were asked to walk within the lines marked with tape on the floor. The wide path was set individually at 50% of the distance between each participant's ASIS [44].

B. Walking around an obstacle (WO): Participants were instructed to alternately walk around the cones on both the right and left sides (zig-zag patterns). Each cone was placed at a distance of 61 cm in width and 75 cm in length [45].

C. Walking straight with horizontal head turns (W-HH): Participants were asked to continue walking straight while turning their heads once to the right and then once to the left, respectively [46]. The assessor instructed the participants to “look right” or “look left” in consecutive order, every 1 meter.

D. Walking straight with vertical head turns (W-VH): Participants were requested to keep walking straight while alternately tipping their heads up and down. The assessor instructed the participants to “look up” or “look down” in consecutive order, every 1 meter.

Fig 2. Gait assessment under complex motor walking tasks: (A) Walking a narrow path, (B) Walking around an obstacle, (C) Walking while horizontal head turns, and (D) Walking while vertical head turns.

For all walking tasks, participants were asked to walk at a self-selected speed over a 10-meter distance for 2 trials in each condition (for a total of 8 trials). All complex motor walking tasks were randomized. A 1-meter distance from the beginning and end of the walkway was excluded from the data analysis to discourage gait acceleration and deceleration events. Participants were given a demonstration before testing, and standard instructions were provided throughout the trials. A 1-minute resting period was allowed in each trial.

Gait smoothness definition

Gait smoothness, expressed as the IH, is a metric of gait quality that determines the rhythmic intersegmental coordination of trunk and pelvis movement within a stride by exploiting the periodicity of the signal [20]. The IH was calculated from the power of the first six harmonics estimated through a discrete Fourier transform (DFT). As calculated by a DFT, P_0 is the power spectral density of the fundamental frequency (first harmonic) and $\sum_{i=0}^{5} P$ is the cumulative sum of the powers of the fundamental frequency and the first five superharmonics (multiplication of the fundamental frequency) (Equations 1-3). The value of IH showed numeric and unitless, in which a power ratio of 1 denotes perfect gait smoothness [20]. The IH measures while performing complex motor walking tasks were quantified into three directions of acceleration: AP, ML, and VT.

$$IH_{AP} = \frac{P_{0\_AP}}{\sum_{i=0}^{5} P\_AP_i} \quad (1)$$

$$IH_{ML} = \frac{P_{0\_ML}}{\sum_{i=0}^{5} P\_ML_i} \quad (2)$$

$$IH_{VT} = \frac{P_{0\_VT}}{\sum_{i=0}^{5} P\_VT_i} \quad (3)$$

Statistical analysis

SPSS software for macOS version 26 (IBM, Armonk, NY) was used to perform the statistical analyses. An independent sample t-test (continuous data) and Chi-Square test (categorial data) were used to compare the participant demographic profiles between the MCI and control groups. The Shapiro-Wilk test was used to determine data normality. A multivariate analysis was employed to address the differences in IH (i.e., AP, ML, and VT) between the two groups for each complex motor walking task (i.e., WN, WO, W-HH, W-VH). A significance level was set at $p < 0.05$.

Results

Participant characteristics

The demographics, cognitive score tests, and baseline gait smoothness of the MCI and control groups are shown in Table 1. There were no significant differences between groups in demographic characteristics ($p > 0.05$). As for cognitive performance, the MCI group had lower scores on MoCA ($p = 0.001$) and Digit Span Test than the control group ($p = 0.01$). Gait speed and IH in all directions (i.e., AP, ML, and VT) at baseline assessment were similar between the two groups ($p > 0.05$).

Table 1. Participant characteristics in the control and MCI groups (N = 36).

Characteristics	Control (n = 18)	MCI (n = 18)	p-value[#]
Age (yr)	67.72 ± 4.63	67.89 ± 4.64	0.92
Gender (Male: Female, n)	6:12	6: 12	1.00
Body mass index (kg/m^2)	24.84 ± 3.12	24.17 ± 2.92	0.51
Education level (yr)	15.89 ± 3.16	15.22 ± 3.44	0.55
Number of medications	1.17 ± 1.20	1.56 ± 1.15	0.33
Number of falls in the past year	0.22 ± 0.55	0.28 ± 0.75	0.80
MSET10 (score, total score = 29)	27.00 ± 1.24	26.67 ± 1.33	0.44
MoCA (score, total score = 30)	26.22 ± 1.52	22.00 ± 1.94	0.001[a]
DST (score, total score = 28)	17.11 ± 3.45	14.39 ± 2.83	0.01[a]
TMT A (sec)	45.74 ± 15.48	49.11 ± 18.76	0.56
TMT B (sec)	104.64 ± 38.14	121.14 ± 41.72	0.22
TMT B-A (sec)	58.90 ± 34.36	72.03 ± 40.72	0.30
Baseline IH-AP	0.68 ± 0.08	0.68 ± 0.10	0.96
Baseline IH-ML	0.75 ± 0.08	0.75 ± 0.10	0.98
Baseline IH-VT	0.85 ± 0.06	0.84 ± 0.05	0.46
Baseline gait speed (m/s)	1.11 ± 0.15	1.02 ± 0.20	0.12

All values are shown as mean ± standard deviation except for gender, number of medications, and falls in the past year.

[#] Independent sample t-test for continuous data and Chi-square test for categorical data.

[a] Significant difference between two groups, $p < 0.05$.

MSET10 = Mental State Examination T10; MoCA = Montreal Cognitive Assessment; DST = Digit Span Test; TMT A = Trail Making Test Part A; TMT B = Trail Making Test Part B; TMT

B-A = subtracting Part B from Part A; IH-AP = Index of harmonicity in the anteroposterior direction; IH-ML = Index of harmonicity in the mediolateral direction; IH-VT = Index of harmonicity in the vertical direction.

Gait smoothness during walking under complex motor tasks

The mean and standard deviation of the IH between groups for the WN, WO, W-HH and W-VH conditions are presented in Table 2. The multivariate analysis revealed a significant difference of the IH-ML in the W-HH condition ($p = 0.04$) between the two groups, in which the MCI group had a lower IH-ML value when compared to the control group. There were no significant differences in all other IH directions and other conditions between the two groups. Moreover, gait speed for each complex motor walking task was similar between the MCI and control groups.

Table 2. The IH values of the MCI and control groups under the complex motor walking tasks.

IH	Control group (n = 18)	MCI group (n = 18)	p-value[#]
**Walking a narrow path (WN)**			
IH-AP	0.76 ± 0.07	0.74 ± 0.11	0.65
IH-ML	0.76 ± 0.09	0.73 ± 0.11	0.34
IH-VT	0.21 ± 0.14	0.23 ± 0.14	0.65
Gait speed (m/s)	0.97 ± 0.21	0.87 ± 0.23	0.17
**Walking around an obstacle (WO)**			
IH-AP	0.18 ± 0.12	0.20 ± 0.15	0.69
IH-ML	0.53 ± 0.14	0.49 ± 0.18	0.55
IH-VT	0.37 ± 0.19	0.36 ± 0.19	0.93
Gait speed (m/s)	0.27 ± 0.05	0.26 ± 0.05	0.82
**Walking straight with horizontal head turns (W-HH)**			
IH-AP	0.37 ± 0.23	0.36 ± 0.18	0.94
IH-ML	0.74 ± 0.12	0.64 ± 0.16	0.04 [a]
IH-VT	0.69 ± 0.16	0.61 ± 0.17	0.16
Gait speed (m/s)	0.88 ± 0.14	0.85 ± 0.16	0.55
**Walking straight with vertical head turns (W-VH)**			
IH-AP	0.61 ± 0.19	0.60 ± 0.18	0.90
IH-ML	0.70 ± 0.16	0.66 ± 0.15	0.44
IH-VT	0.73 ± 0.15	0.62 ± 0.15	0.06
Gait speed (m/s)	0.89 ± 0.13	0.84 ± 0.16	0.39

All values are shown as mean ± standard deviation.

[#] Multivariate analysis.

[a] Significant difference between two groups, $p < 0.05$.

IH = Index of harmonicity; IH-AP = Index of harmonicity in the anteroposterior direction; IH-ML = Index of harmonicity in the mediolateral direction; IH-VT = Index of harmonicity in the vertical direction.

Discussion

Complex walking tasks that require a high demand of gait control may be used to identify early gait impairments in individuals with MCI. The IH, as a measure of gait smoothness, serves as a robust metric for the rhythmic intersegmental coordination of trunk and pelvis movements within a stride, contributing to gait stability. Currently, there are limited studies that investigate gait smoothness in people with MCI during challenging walking tasks relevant to real-life walking situations. The purpose of this preliminary study was to explore the potential for motor stress tests in detecting subtle gait impairment in older adults with MCI. To our knowledge, this study is possibly the first to investigate gait smoothness in older adults with MCI during complex motor tasks necessitating significant walking stability.

Findings from this study demonstrated that the MCI group showed a decrease in gait smoothness in the mediolateral direction while walking with horizontal head turns compared to the peer group. During walking, the gravity sensors of the vestibular system, which are calibrated by the visual input, are responsible for gaze stabilization when the head moves via the vestibulo-ocular reflex [47]. In addition, head direction cells, which are a type of hippocampal neuron, utilize vestibular input to encode heading direction during movement

through the vestibular-hippocampal pathway [48]. Previous studies found degeneration of the hippocampus and entorhinal cortex in the early stage of Alzheimer's disease, including MCI [49, 50]. It is potentially possible that older adults with MCI experience a decline in the hippocampal formation, which is critical for spatial orientation and navigation; thereby potentially influencing the accuracy of current spatial location and directional heading in space. Therefore, the reduced smoothness of rhythmic trunk and pelvis movement in the mediolateral direction observed in older adults with MCI might indicate diminished postural balance control when encountering walking conditions that affect visual and vestibular information, such as horizontal head turns during walking. Singh et al. [51] reported that the horizontal head-turn walking task adversely affected gait performance in older persons with poor lateral balance compared to the head-forward walking task, suggesting that this walking condition requires a higher demand on mediolateral balance control. Our findings align with a previous study, suggesting that the horizontal head turning load on the postural control system leads to gait instability in older adults with MCI, as reflected in a reduction in mediolateral regulation. In the vertical head-turn condition, the results indicated a decrease in gait smoothness in the MCI group compared to the control group, reaching marginal significance ($p = 0.06$). To date, no studies have contrasted the difference in control of the gait between horizontal and vertical head-turn walking in older adults with and without MCI. A previous study exhibited a significant correlation between gaze stability during vertical head movement and gait performance in patients with vestibular disorders ($r = 0.88$) [52]. This finding suggested that gaze stability performance, which is mediated via the vestibulo-ocular reflex, is prominent for successful walking during vertical head movement. Further research is warranted to examine this issue in older adults with MCI.

The present study also revealed that the smoothness of gait did not differ between the MCI and control groups when navigating around an obstacle and walking a narrow path, contrary to our hypothesis. Regarding walking around an obstacle, gait speed of the MCI and control groups was obviously slower than other walking conditions. A prior study found that gait speed influenced the smoothness of gait, with slow gait speeds affecting smoothness more than comfortable and fast speeds [19], as also evidenced in our findings. We postulated that a decreased gait speed while navigating obstacles might be due to older adults adopting a more cautious gait pattern to reduce the magnitudes of the trunk accelerations to ensure walking stability; however, although older people attempt to attenuate gait speed, this does not maintain gait stability (reflecting via a lower IH in all directions), which is in line with previous studies [28, 53]. Therefore, continuous walking around the obstacles would be one of the motor stress tests that compromise gait stability in older adults with and without MCI. Furthermore, the current findings demonstrated that the gait smoothness during the narrow path walking condition was similar for both groups. It is conceivable that walking a narrow path did not pose a sufficient challenge to unveil subtle gait changes in individuals with MCI. Studies examining gait smoothness during narrow path walking among individuals with cognitive limitations are limited, making it challenging to compare the findings.

There are certain limitations in this study that need to be addressed. Participants in the present study were healthy and well-functioning older adults. Thus, our findings may not be generalized to older adults with underlying physical conditions relevant to mobility problems. Moreover, we restricted the use of gait smoothness as an outcome measure due to the fact that it indicates a quality of rhythmic gait regulation; thereby, further studies that include other gait variables such as spatiotemporal, variability, and kinematics measures may provide insight into the whole picture of gait control in older adults with MCI during complex motor walking tasks.

In addition to the triaxial lumbar accelerometer employed in the present study, adding another sensor at the thoracic level would provide a further understanding of how upper and lower intersegmental coordination movements contribute to gait stability. Moreover, a long walking distance that covers more strides would allow for a steady gait outcome analysis. Finally, the findings in this study are preliminary and should be interpreted with caution; thus, future studies are warranted to confirm these findings.

Conclusions

The present exploratory study provides preliminary evidence that older adults with MCI have compromised mediolateral gait stability during horizontal head-turning while walking. Measurement of gait smoothness under this walking condition may allow early detection of gait changes in older adults with MCI.

Acknowledgements

The authors thank Poosri N., Kumfu S., and Lonlue N. for their assistance throughout all data collection. We would also like to thank physical therapists and staff in the subdistrict administrative organization and the elderly school for their contributions to participant recruitment. Finally, we would like to thank all the participants for their time and willingness to participate.

References

1. Yogev- Seligmann G, Hausdorff JM, Giladi N. The role of executive function and attention in gait. Mov Disord. 2008;23(3):329-42. doi: 10.1002/mds.21720

2. van Iersel MB, Kessels RP, Bloem BR, Verbeek AL, Olde Rikkert MG. Executive functions are associated with gait and balance in community-living elderly people. J Gerontol A Biol Sci Med Sci. 2008;63(12):1344-9. doi: 10.1093/gerona/63.12.1344
3. Isordia Martínez J, Góngora Rivera F, Leal Bailey H, Ortiz Jiménez X. Mild cognitive impairment. Medicina. 2014;16(62):28-36.
4. Maquet D, Lekeu F, Warzee E, Gillain S, Wojtasik V, Salmon E, et al. Gait analysis in elderly adult patients with mild cognitive impairment and patients with mild Alzheimer's disease: simple versus dual task: a preliminary report. Clin Physiol Funct Imaging. 2010;30(1):51-6. doi: 10.1111/j.1475-097X.2009.00903.x
5. Saunders NL, Summers MJ. Longitudinal deficits to attention, executive, and working memory in subtypes of mild cognitive impairment. Neuropsychology. 2011;25(2):237-48. doi: 10.1037/a0021134
6. Black SA, Rush RD. Cognitive and functional decline in adults aged 75 and older. J Am Geriatr Soc. 2002;50(12):1978-86. doi: 10.1046/j.1532-5415.2002.50609.x
7. Herman T, Mirelman A, Giladi N, Schweiger A, Hausdorff JM. Executive control deficits as a prodrome to falls in healthy older adults: a prospective study linking thinking, walking, and falling. J Gerontol A Biol Sci Med Sci. 2010;65(10):1086-92. doi: 10.1093/gerona/glq077
8. Verghese J, Robbins M, Holtzer R, Zimmerman M, Wang C, Xue X, et al. Gait dysfunction in mild cognitive impairment syndromes. J Am Geriatr Soc. 2008;56(7):1244-51. doi: 10.1111/j.1532-5415.2008.01758.x
9. Bahureksa L, Najafi B, Saleh A, Sabbagh M, Coon D, Mohler MJ, et al. The impact of mild cognitive impairment on gait and balance: a systematic review and meta-analysis

of studies using instrumented assessment. Gerontology. 2017;63(1):67-83. doi: 10.1159/000445831

10. Beauchet O, Allali G, Launay C, Herrmann F, Annweiler C. Gait variability at fast-pace walking speed: a biomarker of mild cognitive impairment? J Nutr Health Aging. 2013;17(3):235-9. doi: 10.1007/s12603-012-0394-4

11. Boripuntakul S, Lord SR, Brodie M, Smith ST, Methapatara P, Wongpakaran N, et al. Spatial variability during gait initiation while dual tasking is increased in individuals with mild cognitive impairment. J Nutr Health Aging. 2014;18(3):307-12. doi: 10.1007/s12603-013-0390-3

12. Boripuntakul S, Lord SR, Methapatara P, Sungkarat S. Unplanned stopping strategy in individuals with mild cognitive impairment. Walailak J Sci Technol. 2017;14(1):35-42.

13. Boripuntakul S, Kamnardsiri T, Lord SR, Maiarin S, Worakul P, Sungkarat S. Gait variability during abrupt slow and fast speed transitions in older adults with mild cognitive impairment. PLoS One. 2022;17(10):e0276658. doi: 10.1371/journal.pone.0276658

14. Cinelli M, Patla A, Stuart B. Involvement of the head and trunk during gaze reorientation during standing and treadmill walking. Exp Brain Res. 2007;181(1):183-91. doi: 10.1007/s00221-007-0914-3

15. Cinelli M, Patla A, Stuart B. Age-related differences during a gaze reorientation task while standing or walking on a treadmill. Exp Brain Res. 2008;185(1):157-64. doi: 10.1007/s00221-007-1266-8

16. Leandri M, Cammisuli S, Cammarata S, Baratto L, Campbell J, Simonini M, et al. Balance features in Alzheimer's disease and amnestic mild cognitive impairment. J Alzheimer's Dis. 2009;16(1):113-20. doi: 10.3233/JAD-2009-0928

17. Brach JS, Perera S, VanSwearingen JM, Hile ES, Wert DM, Studenski SA. Challenging gait conditions predict 1-year decline in gait speed in older adults with apparently normal gait. Phys Ther. 2011;91(12):1857-64. doi: 10.2522/ptj.20100387
18. Shumway-Cook A, Patla AE, Stewart A, Ferrucci L, Ciol MA, Guralnik JM. Environmental demands associated with community mobility in older adults with and without mobility disabilities. Phys Ther. 2002;82(7):670-81. doi: 10.1093/ptj/82.7.670
19. Menz HB, Lord SR, Fitzpatrick RC. Acceleration patterns of the head and pelvis when walking on level and irregular surfaces. Gait Posture. 2003;18(1):35-46. doi: 10.1016/s0966-6362(02)00159-5
20. Lamoth C, Beek PJ, Meijer OG. Pelvis-thorax coordination in the transverse plane during gait. Gait Posture. 2002;16(2):101-14. doi: 10.1016/s0966-6362(01)00146-1
21. Lowry KA, Smiley- Oyen AL, Carrel AJ, Kerr JP. Walking stability using harmonic ratios in Parkinson's disease. Mov Disord. 2009;24(2):261-7. doi: 10.1002/mds.22352
22. Moe-Nilssen R. A new method for evaluating motor control in gait under real-life environmental conditions. part 2: gait analysis. Clin Biomech (Bristol, Avon). 1998;13(4-5):328-35. doi: 10.1016/s0268-0033(98)00090-4
23. Yack HJ, Berger RC. Dynamic stability in the elderly: identifying a possible measure. J Gerontol. 1993;48(5):M225-30. doi: 10.1093/geronj/48.5.m225
24. Menz HB, Lord SR, St George R, Fitzpatrick RC. Walking stability and sensorimotor function in older people with diabetic peripheral neuropathy. Arch Phys Med Rehabil. 2004;85(2):245-52. doi: 10.1016/j.apmr.2003.06.015
25. Kikkert LHJ, Vuillerme N, van Campen JP, Appels BA, Hortobágyi T, Lamoth CJC. Gait characteristics and their discriminative power in geriatric patients with and without

cognitive impairment. J Neuroeng Rehabil. 2017;14(1):84. doi: 10.1186/s12984-017-0297-z

26. Pau M, Mulas I, Putzu V, Asoni G, Viale D, Mameli I, et al. Smoothness of gait in healthy and cognitively impaired individuals: a study on Italian elderly using wearable inertial sensor. Sensors (Basel). 2020;20(12):3577. doi: 10.3390/s20123577

27. Lamoth CJ, van Deudekom FJ, van Campen JP, Appels BA, de Vries OJ, Pijnappels M. Gait stability and variability measures show effects of impaired cognition and dual tasking in frail people. J Neuroeng Rehabil. 2011;8(1):1-9. doi: 10.1186/1743-0003-8-2

28. IJmker T, Lamoth CJ. Gait and cognition: the relationship between gait stability and variability with executive function in persons with and without dementia. Gait Posture. 2012;35(1):126-30. doi: 10.1016/j.gaitpost.2011.08.022

29. Marcos G, Santabárbara J, Lopez- Anton R, De- la- Cámara C, Gracia- García P, Lobo E, et al. Conversion to dementia in mild cognitive impairment diagnosed with DSM- 5 criteria and with Petersen's criteria. Acta Psychiatr Scand. 2016;133(5):378-85. doi: 10.1111/acps.12543

30. Kittipongpisal S. The accuracy study of the Thai word list learning test. J Ment Health Thai. 2015;23(2):91-101.

31. Boongird P. Mental state examination T10. Dementia Association of Thailand Newsletter. 2018(10).

32. Hughes CP, Berg L, Danziger WL, Coben LA, Martin RL. A new clinical scale for the staging of dementia. Br J Psychiatry. 1982;140:566-72. doi: 10.1192/bjp.140.6.566

33. Hemrungrojn S, Tangwongchai S, Charoenboon T, Panasawat M, Supasitthumrong T, Chaipresertsud P, et al. Use of the montreal cognitive assessment Thai version to discriminate amnestic mild cognitive impairment from Alzheimer's disease and healthy

controls: machine learning results. Dement Geriatr Cogn Disord. 2021;50(2):183-94. doi: 10.1159/000517822

34. Wongpakaran N, Wongpakaran T, Van Reekum R. The use of GDS-15 in detecting MDD: a comparison between residents in a Thai long-term care home and geriatric outpatients. J Clin Med Res. 2013;5(2):101. doi: 10.4021/jocmr1239w

35. Walsh M, Connolly P, Jenkinson A, O'Brien T. Leg length discrepancy-an experimental study of compensatory changes in three dimensions using gait analysis. Gait Posture. 2000;12(2):156-61. doi: 10.1016/s0966-6362(00)00067-9

36. Azucena L. Digit span: effect of education and culture. Int J Psychol. 2006;41:333-41. doi: 10.1080/00207590500345724

37. Kortte KB, Horner MD, Windham WK. The trail making test, part B: cognitive flexibility or ability to maintain set? Appl Neuropsychol. 2002;9(2):106-9. doi: 10.1207/S15324826AN0902_5

38. Walgaard S, Faber GS, van Lummel RC, van Dieën JH, Kingma I. The validity of assessing temporal events, sub-phases and trunk kinematics of the sit-to-walk movement in older adults using a single inertial sensor. J Biomech. 2016;49(9):1933-7. doi: 10.1016/j.jbiomech.2016.03.010.

39. Van Lummel RC, Walgaard S, Hobert MA, Maetzler W, Van Dieën JH, Galindo-Garre F, et al. Intra-rater, inter-rater and test-retest reliability of an instrumented timed up and go (iTUG) test in patients with Parkinson’s disease. PLoS One. 2016;11(3):e0151881. doi: 10.1371/journal.pone.0151881.

40. van Schooten KS, Pijnappels M, Rispens SM, Elders PJ, Lips P, Daffertshofer A, et al. Daily-life gait quality as predictor of falls in older people: a 1-year prospective cohort study. PLoS One. 2016;11(7):e0158623. doi: 10.1371/journal.pone.0158623

41. van Hees V, Slootmaker S, Groot G, Mechelen W, Van Lummel R. Reproducibility of a triaxial seismic accelerometer (DynaPort). Med Sci Sports Exerc. 2009;41:810-7. doi: 10.1249/MSS.0b013e31818ff636
42. Mazzà C, Alcock L, Aminian K, Becker C, Bertuletti S, Bonci T, et al. Technical validation of real-world monitoring of gait: a multicentric observational study. BMJ Open. 2021;11(12):e050785. doi: 10.1136/bmjopen-2021-050785.
43. Micó-Amigo ME, Kingma I, Ainsworth E, Walgaard S, Niessen M, van Lummel RC, et al. A novel accelerometry-based algorithm for the detection of step durations over short episodes of gait in healthy elderly. J Neuroeng Rehabil. 2016;13(1):1-12. doi: 10.1186/s12984-016-0145-6
44. Gimmon Y, Barash A, Debi R, Snir Y, David YB, Grinshpon J, et al. Application of the clinical version of the narrow path walking test to identify elderly fallers. Arch Gerontol Geriatr. 2016;63:108-13. doi: 10.1016/j.archger.2015.10.010
45. Means KM. The obstacle course: a tool for the assessment of functional balance and mobility in the elderly. J Rehabil Res Dev. 1996;33:413-28.
46. Nakamura T, Kikumoto T, Hoshi F. Proactive postural control of the turning while walking in healthy adults. Phys Ther. 2015;101:e1072-e3. doi: 10.1016/j.physio.2015.03.1959
47. Schubert MC, Minor LB. Vestibulo-ocular physiology underlying vestibular hypofunction. Phys Ther. 2004;84:373-5.
48. Stackman RW, Clark AS, Taube JS. Hippocampal spatial representations require vestibular input. Hippocampus. 2002;12(3):291-303. doi: 10.1002/hipo.1112
49. Anderson ND. State of the science on mild cognitive impairment (MCI). CNS Spectr. 2019; 24:78-87. doi: 10.1017/S1092852918001347

50. Du AT, Schuff N, Amend D, Laakso MP, Hsu YY, Jagust WJ, et al. Magnetic resonance imaging of the entorhinal cortex and hippocampus in mild cognitive impairment and Alzheimer's disease. J Neurol Neurosurg Psychiatry. 2001;71(4):441-7. doi: 10.1136/jnnp.71.4.441
51. Singh H, Sanders O, McCombe Waller S, Bair WN, Beamer B, Creath RA, et al. Relationship between head-turn gait speed and lateral balance function in community-dwelling older adults. Arch Phys Med Rehabil. 2017;98(10):1955-61. doi: 10.1016/j.apmr.2017.05.006.
52. Whitney SL, Marchetti GF, Pritcher M, Furman JM. Gaze stabilization and gait performance in vestibular dysfunction. Gait Posture. 2009;29(2):194-8. doi: 10.1016/j.gaitpost.2008.08.002
53. Menz HB, Lord SR, Fitzpatrick RC. Acceleration patterns of the head and pelvis when walking are associated with risk of falling in community-dwelling older people. J Gerontol A Biol Sci Med Sci. 2003;58(5):M446-52. doi: 10.1093/gerona/58.5.m446

Supporting information

S1 File. Gait smoothness while walking under complex motor walking tasks in the MCI and control groups. Mean, standard deviation, maximum, and minimum values of index of harmonicity of AP, ML, and VT directions for each individual under each complex motor walking tasks: walking a narrow path, walking around an obstacle, horizontal head turns while walking, and vertical head turns while walking. (PDF)

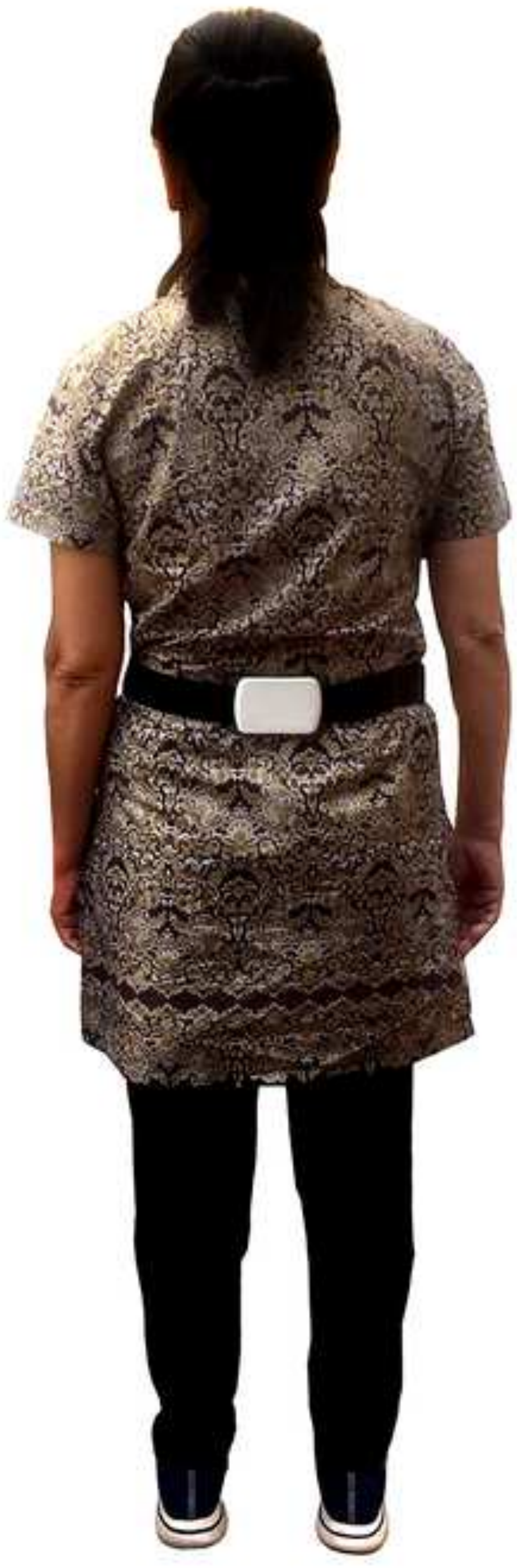

 Click here to access/download;Figure;Fig2.tif

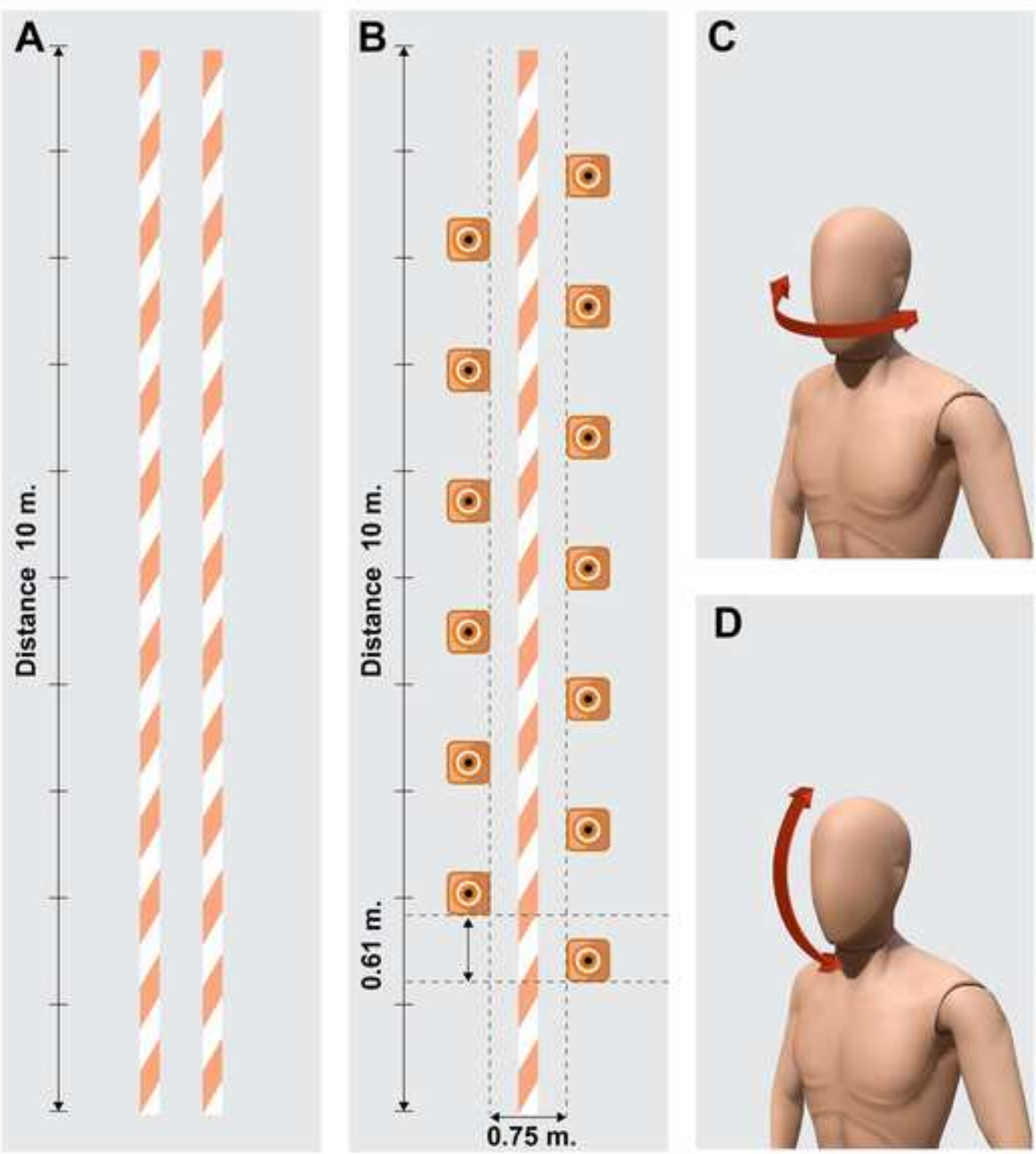

Click here to access/download
Supporting Information
S1_File.pdf

Gait smoothness during high-demand motor walking tasks in older adults with mild cognitive impairment

Thanpidcha Poosri[1], Sirinun Boripuntakul[1*], Somporn Sungkarat[1], Teerawat Kamnardsiri[2], Atiwat Soontornpun[3], Kanokporn Pinyopornpanish[4]

[1] Department of Physical Therapy, Faculty of Associated Medical Sciences, Chiang Mai University, Thailand

[2] Department of Digital Game, College of Arts, Media and Technology, Chiang Mai University, Thailand

[3] Department of Internal Medicine, Faculty of Medicine, Chiang Mai University and Northern Neuroscience Center, Thailand

[4] Department of Family Medicine, Faculty of Medicine, Chiang Mai University, Thailand

* Corresponding author

E-mail: sirinun.b@cmu.ac.th (SB)

Abstract

Early signs of Mild Cognitive Impairment (MCI)-related gait deficits may be detected through the performance of complex walking tasks that require high ~~postural~~ gait control. Gait smoothness is a robust metric of overall body stability during walking. This study aimed to explore gait smoothness during complex walking tasks in older adults with and without MCI. ~~This study aimed to evaluate gait smoothness during complex walking tasks in older adults with and without MCI.~~ Participants were 18 older adults with MCI (mean age = 67.89 ± 4.64 years) and 18 cognitively intact controls (mean age = 67.72 ± 4.63 years). Gait assessment was conducted under four complex walking tasks: walking a narrow path, walking around an obstacle, horizontal head turns while walking, and vertical head turns while walking. ~~Gait assessment under complex walking tasks comprises two types of motor tasks: constrained walking (i.e., walking a narrow path, walking around an obstacle) and head turning while walking (i.e., horizontal head turns, vertical head turns).~~ The index of harmonicity (IH), representing gait smoothness associated with overall body stability, was measured in anteroposterior, mediolateral, and vertical directions. A multivariate analysis was employed to compare the differences in IH between groups for each complex walking task. ~~An independent sample t-test was conducted to compare the differences in IH between groups under constrained walking tasks and head-turning walking tasks.~~ The MCI group demonstrated a reduction of IH in the mediolateral direction during the horizontal head turns than the control group (MCI group = 0.64 ± 0.16, Control group = 0.74 ± 0.12, $p = 0.04$). No significant differences between groups were found for the IH in other directions or walking conditions. These preliminary findings indicate that older adults with MCI have a decline in step regularity in the mediolateral direction during walking with horizontal head turns. Assessment of the smoothness of walking during head turns may be a useful approach to identifying subtle gait

alterations in older adults with MCI, which may facilitate timely gait intervention. These findings indicate that older adults with MCI have a decline in step regularity in the mediolateral direction during walking with horizontal head turns. Assessment of the smoothness of walking during head turns may be a promising approach to identifying subtle gait alterations in older adults with MCI, which may facilitate timely intervention and prevention of falls.

Introduction

Higher level cognitive function, specifically executive function, and attention, plays a vital role in integrating sensorimotor information and controlling movement execution, contributing to safe mobility in complex real-life environments [1, 2]. Previous studies have found that individuals with Mild Cognitive Impairment (MCI), a clinical entity involving cognitive deficit beyond normal aging [3, 4], displayed a decline in attention and executive function [5] which may in turn resultresulting in gait abnormalities and increased risk of falling [6, 7]. Therefore, the early identification of subtle MCI-related changes in gait may have important clinical benefits, ensuring a timely assessment and intervention in gait-related concerns.Therefore, early identification of subtle MCI-related changes in gait may have important clinical benefits for fall prevention.

Existing studies have demonstrated that simple steady-state walking tasks allow the detection of gait changes related to falls in older adults with MCI compared to their cognitively intact peers. [4, 8, 9]. While the above findings were promising, some studies failed to find differences in gait features between persons with MCI and those cognitively intact controls [4, 9, 10]. Discrepancies across prior findings may be due to the variations in gait tasks and outcome measures. It is posited that complex walking tasks could exacerbate subtle MCI-related gait impairment. Currently, it is well documented that the gait impairment of individuals with MCI is significantly pronounced when adding a cognitive load while walking (dual-tasking) [9]. However, previous studies examining MCI-related changes in gait under motor load tasks relevant to a real-life walking situation are scarce. Limited studies have demonstrated a decline in gait regulation of older adults with MCI during transitional gait speeds, including gait initiation, termination, and slow speed transition [11-13]. These findings suggest that motor load tasks may be sensitive in detecting subtle gait changes in older adults with MCI.

Head turning is a fundamental component for successful responses to environmental demands via visual scanning, such as checking for traffic before crossing the road and navigating the barriers in the travel path [14]. Basically, head turning while walking alters visual and vestibular information from the changes of visual field. Specifically, when individuals need to adjust their gait in real-time while head-turning in response to environmental constraints, the vestibular system is more burdened with tracing head movements, which may compromise gait stability [15]. Previous studies have elicited a reduction in postural stability in individuals with MCI partly due to a decrement in the vestibular system linked to a decline in hippocampus function [16]. Therefore, it is plausible that head manipulation while walking could be considered a candidate measure of gait stability in older adults with MCI. Moreover, complex walking tasks concerning everyday environments that require high motor demands in older adults, including walking a narrow path and around an obstacle [17, 18], may challenge gait control in older adults with MCI. Taken together, high-demand motor walking tasks, including head turns while walking, walking a narrow path, and walking around an obstacle, may be used as a motor stress test to amplify a subtle change of gait in older adults with MCI.

~~Therefore, it is plausible that walking stability in people with MCI would be more noticeable when coping with head manipulation while walking. Moreover, constrained walking tasks concerning everyday environments that require motor demands in older adults, including walking a narrow path and around an obstacle [17, 18], may challenge gait control in older adults with MCI. Taken together, head-turning and constrained walking tasks that tap into motor demands may be used as a motor stress test to amplify a subtle change of gait in older adults with MCI.~~

The smoothness of walking, the rhythmic pattern of trunk acceleration, is a dynamic gait metric useful to quantify gait regularity during walking [19-21]. ~~The smoothness of walking, the rhythmic pattern of trunk acceleration, is a robust quality metric useful to quantify gait regularity~~

~~and whole-body dynamic stability during walking, contributing to the risk of falls [19-21].~~ Prior works have demonstrated impaired gait smoothness across various populations, such as cognitively impaired individuals, older adults with restricted neuromuscular control, Parkinson's disease patients, and individuals with sensory impairment, compared to young adults and persons without the disease [21-27]. According to recent studies, trunk accelerations are more sensitive than traditional spatiotemporal metrics for capturing subtle alterations in gait [26, 28]. ~~Thus, it could be considered a candidate measure of gait stability in older adults with early cognitive limitations.~~ Among gait smoothness measures, the index of harmonicity (IH) provides information on the harmonicity of trunk and pelvis movement in conjunction with gait rhythm through spectral analysis of trunk acceleration signals [20]. Specifically, the IH indicates the smoothness of rhythmic intersegmental coordination between the trunk and pelvis throughout the gait cycle, providing insight into balance control during walking [21]. It is well established that dynamic balance and movement coordination during walking are prerequisites for gait stability. With this, the IH has been proposed as a marker indicating the smoothness of gait in relation to walking stability.

To date, gait smoothness has been used to examine gait quality in limited groups and walking conditions. In addition, to the best of our knowledge, no study has investigated the smoothness of gait in people with MCI under high-demand motor tasks relevant to real-life environmental conditions. The aim of this preliminary study was to explore subtle MCI-related changes in gait under a high-demand motor walking task by using gait smoothness as a measurement indicator. It was hypothesized that the MCI group would have less gait smoothness under motor load tasks than the control group. Findings in this study might offer early detection of gait deficits and facilitate timely gait intervention for older adults with MCI.

~~The aim of this study, therefore, was to compare gait smoothness during two types of outdoor walking terrain, including constrained walking tasks (i.e., walking a narrow path and around an obstacle) and head turning while walking tasks (i.e., walking in concurrent with horizontal and vertical head turns) between older adults with and without MCI in a case-control study. It was hypothesized that the MCI group would have less gait smoothness under motor load tasks than the control group. The present findings may provide valuable clinical implications for detecting early signs of gait alterations and potential fall risk in older adults with MCI.~~

Material and methods

Participants

The sample size was calculated based on our pilot study, in which the smallest effect size was chosen. The smallest effect size of 0.22 was derived from the index of harmonicity (IH) in the anteroposterior direction in the condition of walking while making vertical head turns~~walking while the vertical head turns~~ (with baseline gait speed as a covariate). With this, a general linear model with an effect size of 0.22, a power of 0.80, and an alpha level of 0.05 revealed a total sample size of at least 26 participants (13 participants per group).

Eighteen community-dwelling older adults with MCI and 18 healthy-matched controls aged 60 or older were enrolled in the present study. The older adults with MCI were recruited from Maharaj Nakorn Chiang Mai Hospital, a tertiary care hospital in Northern Thailand. The diagnosis of MCI was performed by an experienced neurologist based on Petersen's criteria [29]: (i) subjective complaint of decline in memory on self-or informant report, (ii) isolated memory deficits (determined by the 10-word list learning test ≤ 5) [30], (iii) essentially preserved general cognitive function (determined by the Mental State Examination T10 (MSET10) > 22 or adjusted for

educational level) [31], (iv) largely intact activities of daily living using the clinical review with the patients and informant interview, and (v) clinical dementia (determined by the global Clinical Dementia Rating (CDR) scale < 1) [32]. Additionally, all participants with MCI were required to score on the Montreal Cognitive Assessment (MoCA) ≤ 24 to affirm the presence of subtle cognitive impairment [33]. The cognitively intact control group with similar age (plus or minus two years), gender, body mass index (BMI) (plus or minus two units), and education level (plus or minus two years) was recruited from Chiang Mai community via local community groups and social media advertisements (e.g., Facebook). To be eligible, all participants in the control group were required to have intact cognitive function, as identified by the normal scores of the MSET10 and MoCA.

The inclusion criteria for both groups were being able to walk independently and safely without an assistive device for at least 10 meters and being able to comprehend and follow the instructions. The exclusion criteria were: (i) had major health conditions that affect walking ability or safely~~safety~~ during testing such as musculoskeletal, neurological, cardiorespiratory problems or any unstable medical conditions (e.g., Parkinson's disease, stroke, peripheral vertigo, acute joint pain, uncorrected visual and/or hearing impairment), (ii) had severe dizziness when turning the head in horizontal and/or vertical directions, (iii) had depressive symptoms (determined by the Thai Geriatric Depression Scale-15 score > 6) [34], (iv) current use of sedative drugs, antipsychotic drugs, and narcotic drugs, and (v) had leg length discrepancy > 2.5 cm (assessed from the anterior superior iliac spine (ASIS) to the tip of medial malleolus in a standing position using a flexible tape measure) [35].

In this study, we assessed participants' decision-making capacity by evaluating their comprehension of the research study, including its purpose, procedures, risks, and benefits. Additionally, we appraised their capability to make a rational decision regarding participation in

the study. All participants provided written informed consent prior to enrolling in the study. The institution's ethical review committee for research in humans approved this consent procedure (approval number: AMSEC-65EX-025). ~~The study was approved by the institution's ethical review committee for research in humans (approval number: AMSEC-65EX-025).~~ The start and end of the recruitment period for this study were from the 26th of August 2022 to the 9th of April 2023. All participants provided written informed consent prior to enrolling in the study. Before gait testing, participants in both groups were interviewed about personal demographic data, including age, weight, height, education level, fall incidence within the past year, medical conditions, and medication used. Additionally, participants underwent an assessment of attention and executive functions, which were previously determined to be closely related to gait control, using the Digit Span forward and backward tests and Trail Making Test Parts A and B, respectively [36, 37].

Assessment of gait smoothness under complex motor walking tasks

A triaxial accelerometer device (DynaPort MoveMonitor, McRoberts, The Hague, The Netherlands) with a sampling frequency of 100 Hz was used to measure the IH, which is an indicator of gait smoothness along three orthogonal axes, including anteroposterior (AP), mediolateral (ML), and vertical (VT) directions [38, 39]. Following the standard protocol for accelerometer placement, the accelerometer was mounted at the middle of participants' fifth lumbar spine level and fastened with the supplied elastic belt [40] (Fig 1). In addition, The McRoberts logo on the device was placed outside; with this, the axes x, y, and z were automatically aligned with the direction of gravity. This placement allowed a dependable fixation of the accelerometer and supported the computation of trunk signal detection algorithm [41-42].~~The accelerometer was mounted to the participants' fifth lumbar spine level and fastened with the~~

~~supplied elastic belt [39] (Fig 1).~~ The trunk acceleration signal during walking was transmitted in real-time via Bluetooth to a software database on the personal computer, where the signal-based parameters including IH were subsequently analyzed by an algorithm developed by Micó-Amigo et al. [40].

Fig 1. Placement of triaxial accelerometer on the lower back at the level of the fifth lumbar vertebra.

After a triaxial accelerometer setting, participants were requested to walk along a 10-meter walkway at their comfortable speed for two trials to use as baseline data. As for the gait testing protocol, participants were evaluated for gait smoothness under four complex motor walking tasks on the outdoor walking terrain as follows:

A. Walking a narrow path (WN): Participants were asked to walk within the lines marked with tape on the floor. The wide path was set individually at 50% of the distance between each participant's ASIS [44].

B. Walking around an obstacle (WO): Participants were instructed to alternately walk around the cones on both the right and left sides (zig-zag patterns). Each cone was placed at a distance of 61 cm in width and 75 cm in length [45].

C. Walking straight with horizontal head turns (W-HH): Participants were asked to continue walking straight while turning their heads once to the right and then once to the left, respectively [46]. The assessor instructed the participants to “look right” or “look left” in consecutive order, every 1 meter.

D. Walking straight with vertical head turns (W-VH): Participants were requested to keep walking straight while alternately tipping their heads up and down. The assessor

instructed the participants to “look up” or “look down” in consecutive order, every 1 meter.

As for the gait testing protocol, participants were evaluated for gait smoothness under two types of complex motor walking tasks on the outdoor walkway: Task I: constrained walking tasks, including walking a narrow path and walking around an obstacle (Fig 2), and Task II: head-turning walking tasks, including walking in concurrent with horizontal head turns and walking in concurrent with vertical head turns (Fig 3). For all walking tasks, participants were asked to walk at a self-selected speed over a 10-meter distance for 2 trials in each condition (for a total of 8 trials). The two types of complex motor walking tasks were randomized. A 1-meter distance from the beginning and end of the walkway was excluded from the data analysis to discourage gait acceleration and deceleration events. Participants were given a demonstration before testing, and standard instructions were provided throughout the trials. A 1-minute resting period was allowed in each trial.

Task I: Constrained walking tasks

Walking a narrow path (WN): Participants were asked to walk within the lines marked with tape on the floor. The wide path was set individually at 50% of the distance between each participant's ASIS [41].

Walking around an obstacle (WO): Participants were instructed to alternately walk around the cones on both the right and left sides (zig-zag patterns). Each cone was placed at a distance of 61 cm in width and 75 cm in length [42].

Fig 2. Gait assessment under complex motor walking tasks: (A) Walking a narrow path, (B) Walking around an obstacle, (C) Walking while horizontal head turns, and (D) Walking while vertical head turns.

Fig 2. Gait assessment under constrained walking tasks: (A) Walking a narrow path, (B) Walking around an obstacle.

Task II: Head turning while walking tasks

A. Walking in concurrent with horizontal head turns (W-HH): Participants were asked to continue walking straight while turning their heads once to the right and then once to the left, respectively [43]. The assessor instructed the participants to “look right” or “look left” in consecutive order, every 1 meter.

B. Walking in concurrent with vertical head turns (W-VH): Participants were requested to keep walking straight while alternately tipping their heads up and down. The assessor instructed the participants to “look up” or “look down” in consecutive order, every 1 meter.

Fig 3. Gait assessment under head-turning walking tasks: (A) Walking while horizontal head turns, (B) Walking while vertical head turns.

For all walking tasks, participants were asked to walk at a self-selected speed over a 10-meter distance for 2 trials in each condition (for a total of 8 trials). All complex motor walking tasks were randomized. A 1-meter distance from the beginning and end of the walkway was excluded from the data analysis to discourage gait acceleration and deceleration events. Participants were given a demonstration before testing, and standard instructions were provided throughout the trials. A 1-minute resting period was allowed in each trial.

Gait smoothness definition

Gait smoothness, expressed as the IH, is a metric of gait quality that determines the intersegmental coordination of trunk and pelvis movement within a stride by exploiting the periodicity of the signal [20]. The IH was calculated from the power of the first six harmonics estimated through a discrete Fourier transform (DFT). As calculated by a DFT, P_0 is the power spectral density of the fundamental frequency (first harmonic) and $\sum_{i=0}^{5} P$ is the cumulative sum of the powers of the fundamental frequency and the first five superharmonics (multiplication of the fundamental frequency) (Equations 1-3). The value of IH showed numeric and unitless, in which a power ratio of 1 denotes perfect gait smoothness [20].~~As calculated by a DFT, is the power spectral density of the fundamental frequency (first harmonic) and is the cumulative sum of the powers of the fundamental frequency and the first five superharmonics (multiplication of the fundamental frequency) (Equations 1-3). A power ratio of 1 denotes perfect gait smoothness [20].~~ The IH measures while performing complex motor walking tasks were quantified into three directions of acceleration: AP, ML, and VT.

$$IH_{AP} = \frac{P_{0\_AP}}{\sum_{i=0}^{5} P\_AP_i} \quad (1)$$

$$IH_{ML} = \frac{P_{0\_ML}}{\sum_{i=0}^{5} P\_ML_i} \quad (2)$$

$$IH_{VT} = \frac{P_{0\_VT}}{\sum_{i=0}^{5} P\_VT_i} \quad (3)$$

Statistical analysis

SPSS software for macOS version 26 (IBM, Armonk, NY) was used to perform the statistical analyses. An independent sample t-test (continuous data) and Chi-Square test (categorial data) were used to compare the participant demographic profiles between the MCI and control groups. The

Shapiro-Wilk test was used to determine data normality. A multivariate analysis was employed to address the differences in IH (i.e., AP, ML, and VT) between the two groups for each complex motor walking task (i.e., WN, WO, W-HH, W-VH). A significance level was set at $p < 0.05$.An independent sample t-test was employed to address the differences in IH (i.e., AP, ML, and VT) between the two groups for each constrained walking task (i.e., WN, WO) and head turning walking task (i.e., W-HH, W-VH). A significance level was set at $p < 0.05$, two-sided.

Results

Participant characteristics

The demographics, cognitive score tests, and baseline gait smoothness of the MCI and control groups are shown in Table 1. There were no significant differences between groups in demographic characteristics ($p > 0.05$). As for cognitive performance, the MCI group had lower scores on MoCA ($p = 0.001$) and Digit Span test than the control group ($p = 0.01$). Gait speed and IH in all directions (i.e., AP, ML, and VT) at baseline assessment were similar between the two groups ($p > 0.05$).

Table 1. Participant characteristics in the control and MCI groups (N = 36).

Characteristics	Control (n = 18)	MCI (n = 18)	p-value [#]
Age (yr)	67.72 ± 4.63	67.89 ± 4.64	0.92
Gender (Male: Female, n)	6:12	6: 12	1.00
Body mass index (kg/m^2)	24.84 ± 3.12	24.17 ± 2.92	0.51
Education level (yr)	15.89 ± 3.16	15.22 ± 3.44	0.55
Number of medications	1.17 ± 1.20	1.56 ± 1.15	0.33
Number of falls in the past year	0.22 ± 0.55	0.28 ± 0.75	0.80
MSET10 (score, total score = 29)	27.00 ± 1.24	26.67 ± 1.33	0.44
MoCA (score, total score = 30)	26.22 ± 1.52	22.00 ± 1.94	0.001[a]
DST (score, total score = 28)	17.11 ± 3.45	14.39 ± 2.83	0.01[a]
TMT A (sec)	45.74 ± 15.48	49.11 ± 18.76	0.56
TMT B (sec)	104.64 ± 38.14	121.14 ± 41.72	0.22
TMT B-A (sec)	58.90 ± 34.36	72.03 ± 40.72	0.30
Baseline IH-AP	0.68 ± 0.08	0.68 ± 0.10	0.96
Baseline IH-ML	0.75 ± 0.08	0.75 ± 0.10	0.98
Baseline IH-VT	0.85 ± 0.06	0.84 ± 0.05	0.46
Baseline gait speed (m/s)	1.11 ± 0.15	1.02 ± 0.20	0.12

All values are shown as mean ± standard deviation except for gender, number of medications, and falls in the past year.

[#] Independent sample t-test for continuous data and Chi-square test for categorical data.

[a] Significant difference between two groups, $p < 0.05$.

MSET10 = Mental State Examination T10; MoCA = Montreal Cognitive Assessment; DST = Digit Span Test; TMT A = Trail Making Test Part A; TMT B = Trail Making Test Part B; TMT B-A = subtracting Part B from Part A; IH-AP = Index of harmonicity in the anteroposterior direction; IH-ML = Index of harmonicity in the mediolateral direction; IH-VT = Index of harmonicity in the vertical direction.

Gait smoothness during walking under complex motor tasks

~~Task I: Constrained walking tasks~~

The mean and standard deviation of the IH between groups for the WN and WO conditions are presented in Table 2. The multivariate analysis revealed a significant difference of the IH-ML in the W-HH condition ($p = 0.04$) between the two groups, in which the MCI group had a lower IH-ML value when compared to the control group. There were no significant differences in all other IH directions and other conditions between the two groups. Moreover, gait speed for each complex motor walking task was similar between the MCI and control groups.

~~The independent sample t-test revealed no significant difference for IH in all directions between the MCI and control groups in the WN and WO conditions ($p > 0.05$). In addition, no significant differences were found between both groups in gait speed under the WN and WO conditions.~~

~~Task II: Head-turning walking tasks~~

~~The IH values of the MCI and control groups walking in concurrent with head turn conditions (i.e., W-HH, W-VH) are shown in Table 3. The independent sample t-test revealed a significant difference of the IH-ML in the W-HH condition ($p = 0.04$) between the two groups, in which the MCI group had a lower IH-ML value when compared to the control group. There were no~~

significant differences in all other IH directions and head turning conditions between the two groups. Moreover, gait speed in both the W-HH and W-HV was similar between the two groups ($p > 0.05$).

Table 2. The IH values of the MCI and control groups under the complex motor walking tasks.

IH	Control group (n = 18)	MCI group (n = 18)	p-value[#]
**Walking a narrow path (WN)**			
IH-AP	0.76 ± 0.07	0.74 ± 0.11	0.65
IH-ML	0.76 ± 0.09	0.73 ± 0.11	0.34
IH-VT	0.21 ± 0.14	0.23 ± 0.14	0.65
Gait speed (m/s)	0.97 ± 0.21	0.87 ± 0.23	0.17
**Walking around an obstacle (WO)**			
IH-AP	0.18 ± 0.12	0.20 ± 0.15	0.69
IH-ML	0.53 ± 0.14	0.49 ± 0.18	0.55
IH-VT	0.37 ± 0.19	0.36 ± 0.19	0.93
Gait speed (m/s)	0.27 ± 0.05	0.26 ± 0.05	0.82
**Walking straight with horizontal head turns (W-HH)**			
IH-AP	0.37 ± 0.23	0.36 ± 0.18	0.94
IH-ML	0.74 ± 0.12	0.64 ± 0.16	0.04 [a]
IH-VT	0.69 ± 0.16	0.61 ± 0.17	0.16
Gait speed (m/s)	0.88 ± 0.14	0.85 ± 0.16	0.55
**Walking straight with vertical head turns (W-VH)**			
IH-AP	0.61 ± 0.19	0.60 ± 0.18	0.90

IH-ML	0.70 ± 0.16	0.66 ± 0.15	0.44
IH-VT	0.73 ± 0.15	0.62 ± 0.15	0.06
Gait speed (m/s)	0.89 ± 0.13	0.84 ± 0.16	0.39

All values are shown as mean ± standard deviation.

[#] Multivariate analysis.

[a] Significant difference between two groups, $p < 0.05$.

IH = Index of harmonicity; IH-AP = Index of harmonicity in the anteroposterior direction; IH-ML = Index of harmonicity in the mediolateral direction; IH-VT = Index of harmonicity in the vertical direction.

Table 2. The IH values of the MCI and control groups under the constrained walking tasks.

IH	Control group (n = 18)	MCI group (n = 18)	p-value
**Walking a narrow path**			
IH-AP	0.76 ± 0.07	0.74 ± 0.11	0.65
IH-ML	0.76 ± 0.09	0.73 ± 0.11	0.34
IH-VT	0.21 ± 0.14	0.23 ± 0.14	0.65
Gait speed (m/s)	0.97 ± 0.21	0.87 ± 0.23	0.17
**Walking around an obstacle**			
IH-AP	0.18 ± 0.12	0.20 ± 0.15	0.85
IH-ML	0.53 ± 0.14	0.49 ± 0.18	0.55
IH-VT	0.37 ± 0.19	0.36 ± 0.19	0.93
Gait speed (m/s)	0.27 ± 0.05	0.26 ± 0.05	0.82

All values are shown as mean ± standard deviation.

~~IH = Index of harmonicity; IH-AP = Index of harmonicity in the anteroposterior direction; IH-ML = Index of harmonicity in the mediolateral direction; IH-VT = Index of harmonicity in the vertical direction~~

Discussion

Complex walking tasks that require a high demand of gait control may be used to identify early gait impairments in individuals with MCI. The IH, as a measure of gait smoothness, serves as a robust metric for the rhythmic intersegmental coordination of trunk and pelvis movements within a stride, contributing to gait stability. Currently, there are limited studies that investigate gait smoothness in people with MCI during challenging walking tasks relevant to real-life walking situations. The purpose of this preliminary study was to explore the potential for motor stress tests in detecting subtle gait impairment in older adults with MCI. ~~The purpose of this study was to explore the potential for motor stress tests in improving clinical outcomes by detecting subtle gait impairment in older adults with MCI. Our findings demonstrated that the MCI group showed a decrease in gait smoothness in the mediolateral direction while walking with horizontal head turns compared to the peer group. However, the smoothness of the gait between the two groups did not differ under the constrained walking tasks (i.e., walking a narrow path and around an obstacle).~~ To our knowledge, this study is possibly the first to investigate gait smoothness in older adults with MCI during complex motor tasks necessitating significant walking stability.

Findings from this study demonstrated that the MCI group showed a decrease in gait smoothness in the mediolateral direction while walking with horizontal head turns compared to the peer group. During walking, the gravity sensors of the vestibular system, which are calibrated by the visual input, are responsible for gaze stabilization when the head moves via the vestibulo-ocular reflex [47]. In addition, head direction cells, which are a type of hippocampal neuron, utilize vestibular input to encode heading direction during movement through the vestibular-hippocampal pathway [48]. Previous studies found degeneration of the hippocampus and entorhinal cortex in the early stage of Alzheimer's disease, including MCI [49, 50]. It is potentially possible that older

adults with MCI experience a decline in the hippocampal formation, which is critical for spatial orientation and navigation; thereby potentially influencing the accuracy of current spatial location and directional heading in space. Therefore, the reduced smoothness of rhythmic trunk and pelvis movement in the mediolateral direction observed in older adults with MCI might indicate diminished postural balance control when encountering walking conditions that affect visual and vestibular information, such as horizontal head turns during walking. Singh et al. [51] reported that the horizontal head-turn walking task adversely affected gait performance in older persons with poor lateral balance compared to the head-forward walking task, suggesting that this walking condition requires a higher demand on mediolateral balance control. Our findings align with a previous study, suggesting that the horizontal head turning load on the postural control system leads to gait instability in older adults with MCI, as reflected in a reduction in mediolateral regulation. In the vertical head-turn condition, the results indicated a decrease in gait smoothness in the MCI group compared to the control group, reaching marginal significance ($p = 0.06$). To date, no studies have contrasted the difference in control of the gait between horizontal and vertical head-turn walking in older adults with and without MCI. A previous study exhibited a significant correlation between gaze stability during vertical head movement and gait performance in patients with vestibular disorders ($r = 0.88$) [52]. This finding suggested that gaze stability performance, which is mediated via the vestibulo-ocular reflex, is prominent for successful walking during vertical head movement. Further research is warranted to examine this issue in older adults with MCI.

The present study also revealed that the smoothness of gait did not differ between the MCI and control groups when navigating around an obstacle and walking a narrow path, contrary to our hypothesis. Regarding walking around an obstacle, gait speed of the MCI and control groups was obviously slower than other walking conditions. A prior study found that gait speed influenced the

smoothness of gait, with slow gait speeds affecting smoothness more than comfortable and fast speeds [19], as also evidenced in our findings. We postulated that a decreased gait speed while navigating obstacles might be due to older adults adopting a more cautious gait pattern to reduce the magnitudes of the trunk accelerations to ensure walking stability; however, although older people attempt to attenuate gait speed, this does not maintain gait stability (reflecting via a lower IH in all directions), which is in line with previous studies [28, 53]. Therefore, continuous walking around the obstacles would be one of the motor stress tests that compromise gait stability in older adults with and without MCI. Furthermore, the current findings demonstrated that the gait smoothness during the narrow path walking condition was similar for both groups. It is conceivable that walking a narrow path did not pose a sufficient challenge to unveil subtle gait changes in individuals with MCI. Studies examining gait smoothness during narrow path walking among individuals with cognitive limitations are limited, making it challenging to compare the findings.

Freely turning the head during walking to allow a visual scan is a common movement essential for carrying out daily activities such as checking for oncoming traffic when crossing a street or navigating around obstacles [14]. During walking, the gravity sensors of the vestibular system, which are calibrated by the visual input, are responsible for gaze stabilization when the head or surrounding environment moves [44]. Previous studies demonstrated that head turning during walking, specifically in a horizontal direction, disturbed the dynamic balance control in older adults, both with and without pathological problems [45, 46]. In addition, Singh et al. [47] reported that the horizontal head-turn walking task adversely affected gait performance in older persons with poor lateral balance compared to the head-forward walking task, suggesting that this walking condition requires a high demand on mediolateral balance control. Therefore, walking in concurrent with horizontal head turn may pose older adults with compromised mediolateral balance control at risk of falls. Moreover, a decline in gait smoothness, particularly in the lateral

direction, has been evidenced in older adults with a fear of falling. Our findings extend these findings by suggesting that horizontal head turning induces motor stress on the balance control system, leading to gait instability in older adults with MCI which is reflected through a reduction in mediolateral step-to-step symmetry. It might be possible that older adults with MCI have a decline in hippocampus formation, which is critical for an individual's spatial orientation and navigation via the vestibular-hippocampal pathway. In particular, head direction cells, which are a type of hippocampal neuron, utilize vestibular input to encode heading direction during movement; thereby, an impaired hippocampal function may influence the accuracy of current spatial location and directional heading in space [48]. In line with this postulation, previous studies found degeneration of the hippocampus and entorhinal cortex in the early stage of Alzheimer's disease, including MCI [49, 50]. Therefore, reduced gait smoothness in mediolateral direction observed in older adults with MCI might indicate diminished postural balance control when faced with walking conditions that affect visual and vestibular information such as horizontal head turns during walking. However, a decrease in gait smoothness in the MCI group under the vertical head-turn condition only reached a marginally significance ($p = 0.06$). To date, no studies have contrasted the difference in control of the gait between horizontal and vertical head-turn walking in older adults with and without MCI. A previous study exhibited a strong correlation between gaze stability performance during vertical head movement and gait performance in patients with vestibular disorders [51, 52]. These findings suggested that gaze stability performance, which is mediated via the vestibulo-ocular reflex, is prominent for successful walking during vertical head movement. Further research is warranted to examine this issue in older adults with MCI.

Regarding the constrained walking tasks, including walking a narrow path and around an obstacle, our findings exhibited that gait smoothness between older adults with and without MCI was similar, contrary to our hypothesis. However, for the narrow walking condition, the observed

~~IH value in the vertical direction of both groups appeared to be lower than that in other directions. This may be due to the peculiar nature of narrow path walking, which has unique dynamic gait control characteristics, particularly in the vertical direction. Studies examining gait smoothness via IH value among persons with cognitive limitations, including MCI, while encountering physically constrained walking tasks are limited, making it difficult to compare the findings. To the best of our knowledge, only one previously published study by Pau et al. [26] reported that individuals with early and advanced cognitive decline had reduced gait symmetry expressed as the harmonic ratio, one of the metrics of gait smoothness, in the vertical and anteroposterior directions compared to healthy controls when walking at a normal pace on an unconstrained walking path. Regarding walking around an obstacle, the results showed that the gait speed was markedly slower than all other walking tasks. A prior study found that gait speed influenced the smoothness of gait, with slow gait speeds affecting smoothness more than comfortable and fast speeds [19], as also evidenced in our findings. We postulated that a decreased gait speed while navigating obstacles might be due to older adults adopting a more cautious gait pattern to reduce the magnitudes of the trunk accelerations to ensure walking stability; however, although older people attempt to attenuate gait speed, this does not maintain gait stability (reflecting via a lower IH in all directions), which is in line with previous studies [28, 53]. The continuous walking around the obstacles would be one of the motor stress tests that compromise gait stability in older adults with and without MCI.~~

There are certain limitations in this study that need to be addressed. Participants in the present study were healthy and well-functioning older adults. Thus, our findings may not be generalized to older adults with underlying physical conditions relevant to mobility problems. Moreover, we restricted the use of gait smoothness as an outcome measure due to the fact that it indicates a quality of rhythmic gait regulation; thereby, further studies that include other gait variables such as spatiotemporal, variability, and kinematics measures may provide insight into the whole picture of

gait control in older adults with MCI during complex motor walking tasks. In addition to the triaxial lumbar accelerometer employed in the present study, adding another sensor at the thoracic level would provide a further understanding of how upper and lower intersegmental coordination movements contribute to gait stability. Moreover, a long walking distance that covers more strides would allow for a steady gait outcome analysis. Finally, the findings in this study are preliminary and should be interpreted with caution; thus, future studies are warranted to confirm these findings.

Conclusions

The present exploratory study provides preliminary evidence that older adults with MCI have compromised mediolateral gait stability during horizontal head-turning while walking. Measurement of gait smoothness under this walking condition may allow early detection of gait changes in older adults with MCI.

~~The present study provides evidence that older adults with MCI have compromised mediolateral gait stability during horizontal head-turning while walking. Measurement of gait smoothness under this walking condition may allow early detection of subtle changes in gait control and fall risk in older adults with MCI~~.

Acknowledgements

The authors thank Poosri N., Kumfu S., and Lonlue N. for their assistance throughout all data collection. We would also like to thank physical therapists and staff in the subdistrict administrative organization and the elderly school for their contributions to participant recruitment. Finally, we would like to thank all the participants for their time and willingness to participate.

References

1. Yogev- Seligmann G, Hausdorff JM, Giladi N. The role of executive function and attention in gait. Mov Disord. 2008;23(3):329-42. doi: 10.1002/mds.21720
2. van Iersel MB, Kessels RP, Bloem BR, Verbeek AL, Olde Rikkert MG. Executive functions are associated with gait and balance in community-living elderly people. J Gerontol A Biol Sci Med Sci. 2008;63(12):1344-9. doi: 10.1093/gerona/63.12.1344
3. Isordia Martínez J, Góngora Rivera F, Leal Bailey H, Ortiz Jiménez X. Mild cognitive impairment. Medicina. 2014;16(62):28-36.
4. Maquet D, Lekeu F, Warzee E, Gillain S, Wojtasik V, Salmon E, et al. Gait analysis in elderly adult patients with mild cognitive impairment and patients with mild Alzheimer's disease: simple versus dual task: a preliminary report. Clin Physiol Funct Imaging. 2010;30(1):51-6. doi: 10.1111/j.1475-097X.2009.00903.x
5. Saunders NL, Summers MJ. Longitudinal deficits to attention, executive, and working memory in subtypes of mild cognitive impairment. Neuropsychology. 2011;25(2):237-48. doi: 10.1037/a0021134
6. Black SA, Rush RD. Cognitive and functional decline in adults aged 75 and older. J Am Geriatr Soc. 2002;50(12):1978-86. doi: 10.1046/j.1532-5415.2002.50609.x
7. Herman T, Mirelman A, Giladi N, Schweiger A, Hausdorff JM. Executive control deficits as a prodrome to falls in healthy older adults: a prospective study linking thinking, walking, and falling. J Gerontol A Biol Sci Med Sci. 2010;65(10):1086-92. doi: 10.1093/gerona/glq077

8. Verghese J, Robbins M, Holtzer R, Zimmerman M, Wang C, Xue X, et al. Gait dysfunction in mild cognitive impairment syndromes. J Am Geriatr Soc. 2008;56(7):1244-51. doi: 10.1111/j.1532-5415.2008.01758.x

9. Bahureksa L, Najafi B, Saleh A, Sabbagh M, Coon D, Mohler MJ, et al. The impact of mild cognitive impairment on gait and balance: a systematic review and meta-analysis of studies using instrumented assessment. Gerontology. 2017;63(1):67-83. doi: 10.1159/000445831

10. Beauchet O, Allali G, Launay C, Herrmann F, Annweiler C. Gait variability at fast-pace walking speed: a biomarker of mild cognitive impairment? J Nutr Health Aging. 2013;17(3):235-9. doi: 10.1007/s12603-012-0394-4

11. Boripuntakul S, Lord SR, Brodie M, Smith ST, Methapatara P, Wongpakaran N, et al. Spatial variability during gait initiation while dual tasking is increased in individuals with mild cognitive impairment. J Nutr Health Aging. 2014;18(3):307-12. doi: 10.1007/s12603-013-0390-3

12. Boripuntakul S, Lord SR, Methapatara P, Sungkarat S. Unplanned stopping strategy in individuals with mild cognitive impairment. Walailak J Sci Technol. 2017;14(1):35-42.

13. Boripuntakul S, Kamnardsiri T, Lord SR, Maiarin S, Worakul P, Sungkarat S. Gait variability during abrupt slow and fast speed transitions in older adults with mild cognitive impairment. PLoS One. 2022;17(10):e0276658. doi: 10.1371/journal.pone.0276658

14. Cinelli M, Patla A, Stuart B. Involvement of the head and trunk during gaze reorientation during standing and treadmill walking. Exp Brain Res. 2007;181(1):183-91. doi: 10.1007/s00221-007-0914-3

15. Cinelli M, Patla A, Stuart B. Age-related differences during a gaze reorientation task while standing or walking on a treadmill. Exp Brain Res. 2008;185(1):157-64. doi: 10.1007/s00221-007-1266-8

16. Leandri M, Cammisuli S, Cammarata S, Baratto L, Campbell J, Simonini M, et al. Balance features in Alzheimer's disease and amnestic mild cognitive impairment. J Alzheimer's Dis. 2009;16(1):113-20. doi: 10.3233/JAD-2009-0928
17. Brach JS, Perera S, VanSwearingen JM, Hile ES, Wert DM, Studenski SA. Challenging gait conditions predict 1-year decline in gait speed in older adults with apparently normal gait. Phys Ther. 2011;91(12):1857-64. doi: 10.2522/ptj.20100387
18. Shumway-Cook A, Patla AE, Stewart A, Ferrucci L, Ciol MA, Guralnik JM. Environmental demands associated with community mobility in older adults with and without mobility disabilities. Phys Ther. 2002;82(7):670-81.
19. Menz HB, Lord SR, Fitzpatrick RC. Acceleration patterns of the head and pelvis when walking on level and irregular surfaces. Gait Posture. 2003;18(1):35-46. doi: 10.1016/s0966-6362(02)00159-5
20. Lamoth C, Beek PJ, Meijer OG. Pelvis-thorax coordination in the transverse plane during gait. Gait Posture. 2002;16(2):101-14. doi: 10.1016/s0966-6362(01)00146-1
21. Lowry KA, Smiley- Oyen AL, Carrel AJ, Kerr JP. Walking stability using harmonic ratios in Parkinson's disease. Mov Disord. 2009;24(2):261-7. doi: 10.1002/mds.22352
22. Moe-Nilssen R. A new method for evaluating motor control in gait under real-life environmental conditions. part 2: gait analysis. Clin Biomech (Bristol, Avon). 1998;13(4-5):328-35. doi: 10.1016/s0268-0033(98)00090-4
23. Yack HJ, Berger RC. Dynamic stability in the elderly: identifying a possible measure. J Gerontol. 1993;48(5):M225-30. doi: 10.1093/geronj/48.5.m225
24. Menz HB, Lord SR, St George R, Fitzpatrick RC. Walking stability and sensorimotor function in older people with diabetic peripheral neuropathy. Arch Phys Med Rehabil. 2004;85(2):245-52. doi: 10.1016/j.apmr.2003.06.015

25. Kikkert LHJ, Vuillerme N, van Campen JP, Appels BA, Hortobágyi T, Lamoth CJC. Gait characteristics and their discriminative power in geriatric patients with and without cognitive impairment. J Neuroeng Rehabil. 2017;14(1):84. doi: 10.1186/s12984-017-0297-z

26. Pau M, Mulas I, Putzu V, Asoni G, Viale D, Mameli I, et al. Smoothness of gait in healthy and cognitively impaired individuals: a study on Italian elderly using wearable inertial sensor. Sensors (Basel). 2020;20(12):3577. doi: 10.3390/s20123577

27. Lamoth CJ, van Deudekom FJ, van Campen JP, Appels BA, de Vries OJ, Pijnappels M. Gait stability and variability measures show effects of impaired cognition and dual tasking in frail people. J Neuroeng Rehabil. 2011;8(1):1-9. doi: 10.1186/1743-0003-8-2

28. IJmker T, Lamoth CJ. Gait and cognition: the relationship between gait stability and variability with executive function in persons with and without dementia. Gait Posture. 2012;35(1):126-30. doi: 10.1016/j.gaitpost.2011.08.022

29. Marcos G, Santabárbara J, Lopez- Anton R, De- la- Cámara C, Gracia- García P, Lobo E, et al. Conversion to dementia in mild cognitive impairment diagnosed with DSM- 5 criteria and with Petersen's criteria. Acta Psychiatr Scand. 2016;133(5):378-85. doi: 10.1111/acps.12543

30. Kittipongpisal S. The accuracy study of the Thai word list learning test. J Ment Health Thai. 2015;23(2):91-101.

31. Boongird P. Mental state examination T10. Dementia Association of Thailand Newsletter. 2018(10).

32. Hughes CP, Berg L, Danziger WL, Coben LA, Martin RL. A new clinical scale for the staging of dementia. Br J Psychiatry. 1982;140:566-72. doi: 10.1192/bjp.140.6.566

33. Hemrungrojn S, Tangwongchai S, Charoenboon T, Panasawat M, Supasitthumrong T, Chaipresertsud P, et al. Use of the montreal cognitive assessment Thai version to discriminate amnestic mild cognitive impairment from Alzheimer's disease and healthy controls: machine learning results. Dement Geriatr Cogn Disord. 2021;50(2):183-94. doi: 10.1159/000517822

34. Wongpakaran N, Wongpakaran T, Van Reekum R. The use of GDS-15 in detecting MDD: a comparison between residents in a Thai long-term care home and geriatric outpatients. J Clin Med Res. 2013;5(2):101. doi: 10.4021/jocmr1239w

35. Walsh M, Connolly P, Jenkinson A, O'Brien T. Leg length discrepancy-an experimental study of compensatory changes in three dimensions using gait analysis. Gait Posture. 2000;12(2):156-61. doi: 10.1016/s0966-6362(00)00067-9

36. Azucena L. Digit span: effect of education and culture. Int J Psychol. 2006;41:333-41. doi: 10.1080/00207590500345724

37. Kortte KB, Horner MD, Windham WK. The Trail Making Test, part B: cognitive flexibility or ability to maintain set? Appl Neuropsychol. 2002;9(2):106-9. doi: 10.1207/S15324826AN0902_5

38. Walgaard S, Faber GS, van Lummel RC, van Dieën JH, Kingma I. The validity of assessing temporal events, sub-phases and trunk kinematics of the sit-to-walk movement in older adults using a single inertial sensor. J Biomech. 2016;49(9):1933-7. doi: 10.1016/j.jbiomech.2016.03.010.~~Huijben B, Van Schooten K, Van Dieën J, Pijnappels M. The effect of walking speed on quality of gait in older adults. Gait Posture. 2018;65:112-6. doi: 10.1016/j.gaitpost.2018.07.004~~

39. Van Lummel RC, Walgaard S, Hobert MA, Maetzler W, Van Dieën JH, Galindo-Garre F, et al. Intra-rater, inter-rater and test-retest reliability of an instrumented timed up and go

(iTUG) test in patients with Parkinson's disease. PLoS One. 2016;11(3):e0151881. doi: 10.1371/journal.pone.0151881.~~van Schooten KS, Pijnappels M, Rispens SM, Elders PJ, Lips P, Daffertshofer A, et al. Daily-life gait quality as predictor of falls in older people: a 1-year prospective cohort study. PLoS One. 2016;11(7):e0158623. doi: 10.1371/journal.pone.0158623~~

40. van Schooten KS, Pijnappels M, Rispens SM, Elders PJ, Lips P, Daffertshofer A, et al. Daily-life gait quality as predictor of falls in older people: a 1-year prospective cohort study. PLoS One. 2016;11(7):e0158623. doi: 10.1371/journal.pone.0158623~~Micó-Amigo ME, Kingma I, Ainsworth E, Walgaard S, Niessen M, van Lummel RC, et al. A novel accelerometry-based algorithm for the detection of step durations over short episodes of gait in healthy elderly. J Neuroeng Rehabil. 2016;13(1):1-12. doi: 10.1186/s12984-016-0145-6~~

41. van Hees V, Slootmaker S, Groot G, Mechelen W, Van Lummel R. Reproducibility of a triaxial seismic accelerometer (DynaPort). Med Sci Sports Exerc. 2009;41:810-7. doi: 10.1249/MSS.0b013e31818ff636

42. Mazzà C, Alcock L, Aminian K, Becker C, Bertuletti S, Bonci T, et al. Technical validation of real-world monitoring of gait: a multicentric observational study. BMJ Open. 2021;11(12):e050785. doi: 10.1136/bmjopen-2021-050785.

43. Micó-Amigo ME, Kingma I, Ainsworth E, Walgaard S, Niessen M, van Lummel RC, et al. A novel accelerometry-based algorithm for the detection of step durations over short episodes of gait in healthy elderly. J Neuroeng Rehabil. 2016;13(1):1-12. doi: 10.1186/s12984-016-0145-6

44. Gimmon Y, Barash A, Debi R, Snir Y, David YB, Grinshpon J, et al. Application of the clinical version of the narrow path walking test to identify elderly fallers. Arch Gerontol Geriatr. 2016;63:108-13. doi: 10.1016/j.archger.2015.10.010

45. Means KM. The obstacle course: a tool for the assessment of functional balance and mobility in the elderly. J Rehabil Res Dev. 1996;33:413-28.

46. Nakamura T, Kikumoto T, Hoshi F. Proactive postural control of the turning while walking in healthy adults. Phys Ther. 2015;101:e1072-e3. doi: 10.1016/j.physio.2015.03.1959

47. Schubert MC, Minor LB. Vestibulo-ocular physiology underlying vestibular hypofunction. Phys Ther. 2004;84:373-5.

48. Stackman RW, Clark AS, Taube JS. Hippocampal spatial representations require vestibular input. Hippocampus. 2002;12(3):291-303. doi: 10.1002/hipo.1112

49. Anderson ND. State of the science on mild cognitive impairment (MCI). CNS Spectr. 2019; 24:78-87. doi: 10.1017/S1092852918001347

50. Du AT, Schuff N, Amend D, Laakso MP, Hsu YY, Jagust WJ, et al. Magnetic resonance imaging of the entorhinal cortex and hippocampus in mild cognitive impairment and Alzheimer's disease. J Neurol Neurosurg Psychiatry. 2001;71(4):441-7. doi: 10.1136/jnnp.71.4.441

51. Singh H, Sanders O, McCombe Waller S, Bair WN, Beamer B, Creath RA, et al. Relationship between head-turn gait speed and lateral balance function in community-dwelling older adults. Arch Phys Med Rehabil. 2017;98(10):1955-61. doi: 10.1016/j.apmr.2017.05.006.

52. Whitney SL, Marchetti GF, Pritcher M, Furman JM. Gaze stabilization and gait performance in vestibular dysfunction. Gait Posture. 2009;29(2):194-8. doi: 10.1016/j.gaitpost.2008.08.002

53. Menz HB, Lord SR, Fitzpatrick RC. Acceleration patterns of the head and pelvis when walking are associated with risk of falling in community-dwelling older people. J Gerontol A Biol Sci Med Sci. 2003;58(5):M446-52. doi: 10.1093/gerona/58.5.m446

~~Gimmon Y, Barash A, Debi R, Snir Y, David YB, Grinshpon J, et al. Application of the clinical version of the narrow path walking test to identify elderly fallers. Arch Gerontol Geriatr. 2016;63:108-13. doi: 10.1016/j.archger.2015.10.010~~

~~42. Means KM. The obstacle course: a tool for the assessment of functional balance and mobility in the elderly. J Rehabil Res Dev. 1996;33:413-28.~~

~~43. Nakamura T, Kikumoto T, Hoshi F. Proactive postural control of the turning while walking in healthy adults. Phys Ther. 2015;101:e1072-3. doi: 10.1016/j.physio.2015.03.1959~~

~~44. Schubert MC, Minor LB. Vestibulo-ocular physiology underlying vestibular hypofunction. Phys Ther. 2004;84:373-5.~~

~~45. Marchetti GF, Whitney SL, Blatt PJ, Morris LO, Vance JM. Temporal and spatial characteristics of gait during performance of the Dynamic Gait Index in people with and people without balance or vestibular disorders. Phys Ther. 2008;88(5):640-51. doi: 10.2522/ptj.20070130~~

~~46. D'Silva L, Chalise P, Rippee M, Devos H. Challenging the vestibular system affects gait speed and cognitive workload in chronic mild traumatic brain injury and healthy adults. Front Neurol. 2022;13:819169. doi: 10.3389/fneur.2022.819169~~

~~47. Singh H, Sanders O, McCombe Waller S, Bair WN, Beamer B, Creath RA, et al. Relationship between head-turn gait speed and lateral balance function in community-dwelling older adults. Arch Phys Med Rehabil. 2017;98(10):1955-61. doi: 10.1016/j.apmr.2017.05.006~~

48. Stackman RW, Clark AS, Taube JS. Hippocampal spatial representations require vestibular input. Hippocampus. 2002;12(3):291-303. doi: 10.1002/hipo.1112

49. Anderson ND. State of the science on mild cognitive impairment (MCI). CNS Spectr. 2019; 24:78-87. doi: 10.1017/S1092852918001347

50. Du AT, Schuff N, Amend D, Laakso MP, Hsu YY, Jagust WJ, et al. Magnetic resonance imaging of the entorhinal cortex and hippocampus in mild cognitive impairment and Alzheimer's disease. J Neurol Neurosurg Psychiatry. 2001;71(4):441-7. doi: 10.1136/jnnp.71.4.441

51. Whitney SL, Marchetti GF, Pritcher M, Furman JM. Gaze stabilization and gait performance in vestibular dysfunction. Gait Posture. 2009;29(2):194-8. doi: 10.1016/j.gaitpost.2008.08.002

52. Whitney SL, Marchetti GF, Schade A, Wrisley DM. The sensitivity and specificity of the Timed "Up & Go" and the Dynamic Gait Index for self-reported falls in persons with vestibular disorders. J Vestib Res. 2004;14(5):397-409.

53. Menz HB, Lord SR, Fitzpatrick RC. Acceleration patterns of the head and pelvis when walking are associated with risk of falling in community-dwelling older people. J Gerontol A Biol Sci Med Sci. 2003;58(5):M446-M52. doi: 10.1093/gerona/58.5.m446

Supporting information

S1 File. Gait smoothness while walking under complex motor walking tasks in the MCI and control groups. Mean, standard deviation, maximum, and minimum values of index of harmonicity of AP, ML, and VT directions for each individual under each complex motor walking tasks: walking a narrow path, walking around an obstacle, horizontal head turns while walking,

and vertical head turns while walking. (PDF) **S1 File. Gait smoothness while walking over two types of complex motor walking tasks in the MCI and control groups.** Mean, standard deviation, maximum, and minimum values of index of harmonicity of AP, ML, and VT directions for each individual under complex motor walking tasks: I constrained walking tasks (i.e., walking a narrow path, walking around an obstacle) and II head turning while walking tasks (i.e., horizontal head turns, vertical head turns). (PDF)

Response to Reviewers' Comments

Journal Requirements:

When submitting your revision, we need you to address these additional requirements.

Comment # 1. Please ensure that your manuscript meets PLOS ONE's style requirements, including those for file naming. The PLOS ONE style templates can be found at https://journals.plos.org/plosone/s/file?id=wjVg/PLOSOne_formatting_sample_main_body.pdf and https://journals.plos.org/plosone/s/file?id=ba62/PLOSOne_formatting_sample_title_authors_affiliations.pdf

Response # 1. We have formatted our manuscript according to PLOS ONE's style requirements.

Comment # 2. Please describe in your methods section how capacity to provide consent was determined for the participants in this study. Please also state whether your ethics committee or IRB approved this consent procedure. If you did not assess capacity to consent please briefly outline why this was not necessary in this case.

Response # 2. We have included this information in the methods section as follows (page 7, line 150-155):

"In this study, we assessed participants' decision-making capacity by evaluating their comprehension of the research study, including its purpose, procedures, risks, and benefits. Additionally, we appraised their capability to make a rational decision regarding participation in the study. All participants provided written informed consent prior to enrolling in the study. The institution's ethical review committee for research in humans approved this consent procedure (approval number: AMSEC-65EX-025)."

Reviewers' comments:

Reviewer's Responses to Questions

Reviewer # 1:

This quasi-experimental study was well-conceived, and the manuscript is well-organized and written. I made a few comments on word choice and have several questions/need for additional information (see comments made directly on the .pdf).

Response: Thank you very much.

We have revised the words and sentences throughout the manuscript according to the reviewer's suggestion as follows:

- Page 3, line 50: We have revised "resulting" to "result".
- Page 6, line 118: We have revised "walking while the vertical head turns" to "walking while making vertical head turns".
- Page 7, line 142: We have revised "safety" to "safely".
- Page 9, line 192 and 196: We have revised "walking in concurrent with horizontal/vertical head turns" to "walking straight with horizontal/vertical head turns".
- Page 15, line 295: We have revised "in mediolateral direction" to "in the mediolateral direction".
- Page 16, line 311: We have revised "high" to "higher".
- Page 16, line 315: We have revised "a marginal" to "marginal".
- Page 17, line 335: We have revised "the narrow walking" to "the narrow path walking".

Comment # 1. Please provide references attesting to the accuracy (validity) and precision (reliability) of this device.

Response # 1. We have included the references about the validity and reliability of the triaxial accelerometer device (DynaPort MoveMonitor, McRoberts) in the methods section (page 8, line 168).

Comment # 2. Line 166: please spell this abbreviation out and put IH in parentheses.

Response # 2. We spelled out the full name and abbreviation of IH on page 4, line 88. After that, we use the "IH" throughout the manuscript to keep the format consistent.

Comment # 3. Please describe any calibration procedure that might be used for this device prior to data collection.

Response # 3. The triaxial accelerometer device (DynaPort MoveMonitor, McRoberts) used in the present study has been calibrated prior to being dispatched (1). We followed the standardized procedures from previous studies to ensure the accuracy of gait measurement (1-2).

We have included the information in the methods section as follows (page 8, line 168-174):

Following the standard protocol for accelerometer placement, the accelerometer was mounted at the middle of participants' fifth lumbar spine level and fastened with the supplied elastic belt [40] (Fig 1). In addition, The McRoberts logo on the device was placed outside; with this, the axes x, y, and z were automatically aligned with the direction of gravity. This placement allowed a dependable fixation of the accelerometer and supported the computation of trunk signal detection algorithm [41-42]"

References:

1. McRoberts [Internet]. Netherlands: McRoberts BV; 2023 [updated 2023; cited 2023 Dec 1]. Available from: https://www. https://www.mcroberts.nl/
2. van Hees V, Slootmaker S, Groot G, Mechelen W, Van Lummel R. Reproducibility of a triaxial seismic accelerometer (DynaPort). Med Sci Sports Exerc. 2009;41:810-7. doi: 10.1249/MSS.0b013e31818ff636
3. Mazzà C, Alcock L, Aminian K, Becker C, Bertuletti S, Bonci T, et al. Technical validation of real-world monitoring of gait: a multicentric observational study. BMJ Open. 2021;11(12):e050785. doi: 10.1136/bmjopen-2021-050785.

Comment # 4. Line 217: should be "IH is..."

Response # 4. We have rechecked and it should be "Po is….". We aimed to define the formula for IH calculation.

Comment # 5. Line 219: I assume that this measure is unitless. If so, please declare this or provide the units of the measure.

Response # 5. Yes, it is unitless. We have mentioned this in the text (page 10, line 219-220).

Comment # 6. Line 222: suggest: "antero-posterior (AP), medio-lateral (ML), and vertical (VT)

Response # 6. We spelled out the full name and abbreviation of anteroposterior, mediolateral, and vertical on page 8, line 167-168. After that, we use the "AP", "ML", and "VT" throughout the manuscript to keep the format consistent.

Comment # 7. Please define P and Po in the part of gait smoothness definition.

Response # 7. We have defined P and Po (page 10, line 217-219).

Comment # 8. In the statistical analysis, should a correction be made with multiple t-tests, e.g., Bonferonni?

Response # 8. As this is an exploratory analysis, no adjustment was made for multiple comparisons of outcomes. However, as the dependent variables (i.e., IH in the AP, ML, and VT directions) of each walking task are related, the statistician suggested that a multivariate analysis should be conducted instead of an independent t-test. The results are still the same, wherein the MCI group had a significantly lower IH in the ML direction compared to the control group under horizontal head turns while walking ($p = 0.04$).

We have revised the statistical analysis, and acknowledged in the study limitation that findings are preliminary.

Comment # 9. In the discussion part, prior to restating the study purpose please briefly state the rationale/need for the study.

Response # 9. We have added a brief explanation of the rationale in the discussion part as follows (page 15, line 285-290):

"Complex walking tasks that require a high demand of gait control may be used to identify early gait impairments in individuals with MCI. The IH, as a measure of gait

smoothness, serves as a robust metric for the rhythmic intersegmental coordination of trunk and pelvis movements within a stride, contributing to gait stability. Currently, there are limited studies that investigate gait smoothness in people with MCI during challenging walking tasks relevant to real-life walking situations."

Comment # 10. Line 317-319: be careful - correlation is not cause/effect. You should report the precise correlation value and let the reader decide magnitude because I have seen r values of 0.3 which were statistically significant and might be called strong, when surely this number may not be clinically relevant, i.e., a $r = 0.3$ is an $r^2 = 0.09$ which is relatively meaningless.

Response # 10. Thank you for pointing this out. We have revised this sentence as the reviewer's suggestion (page 16, line 319-321).

Reviewer # 2:

Summary: The authors investigated gait smoothness in adults with and without mild cognitive impairment (MCI) during complex tasks that are common in daily life. The authors quantified gait smoothness during walking as the Index of Harmonicity (IH) of a lumbar-mounted accelerometer. The IH of adults with and without MCI were compared across a narrow path task, an obstacle avoidance task, and a head turning task. The authors found moderate differences only in the mediolateral IH during the heading turning task, concluding that the IH during a head turning task may provide a valuable outcome to be used as early fall risk detection in people with MCI.

The results in this study have not been published elsewhere, to my knowledge. The manuscript is intelligible and with only minor typos, though focusing the writing on a specific target impact would improve the manuscript's clarity.

The authors provide strong mechanistic and empirical support for their premise: that experimental paradigms challenging motor and vestibular function would reveal subtle gait deficits in adults with (vs. without) MCI. However, the limited support for using the IH as a measure of fall risk and the limitations of the experimental methodology do not inspire confidence in the robustness of the primary outcomes or the knowledge gained from the study. Combined with limitations in the statistical analysis, these limitations provide poor support for the manuscript's conclusions. I do not think that the study's conclusions can be supported without significant additional data collection and revised analyses. Therefore, I do not recommend the manuscript for publication. I have provided specific comments below.

Comment # 1. The focus of the manuscript should be made more precise. The focus and implications seem to alternate between early fall risk detection, improving clinical outcomes, and characterizing stability deficits in adults with MCI.

Response # 1. We thank the reviewer for the thorough assessment and valuable comments on the manuscript. We apologize for the confusion caused by the writing that appears to alternate the study's focus back and forth. The focus of this study was to identify gait stability deficits in older adults with MCI. Specifically, we aimed to explore subtle MCI-related changes in gait under a high-demand motor walking task

by using gait smoothness as a measurement indicator. Findings in this study might offer early detection of gait deficits and facilitate timely gait intervention for older adults with MCI. We have revised the introduction, objective, and conclusion based on this comment and the comment #2 (page 3, line 51-52; page 5, line 98-102; page 18, line 355-358).

Comment # 2. I am concerned by conclusions drawn from a single significant result amid many comparisons. Because multiple comparisons would support the same hypothesis-that gait smoothness differentially changes in adults with versus without MCI- the authors should correct for multiple comparisons. Given the single significant result ($p = 0.04$), it is likely that no comparisons would be significant after correction.
Response # 2. We appreciate the important points that the reviewer raises. This study aimed to explore which complex walking task(s) could differentiate gait changes between individuals with MCI and cognitively intact controls. As this is an exploratory analysis, no adjustment was made for multiple comparisons of outcomes. Each complex walking task (i.e., walking a narrow path, walking around an obstacle, horizontal head turns while walking, and vertical head turns while walking) was established to be a high-demand motor task that requires a unique and independent underlying mechanism of gait control. Further, as the dependent variables (i.e., IH in the AP, ML, and VT directions) of each walking task are related, the statistician suggested that a multivariate analysis should be conducted instead of an independent t-test. The results are still the same, wherein the MCI group had a significantly lower IH in the ML direction compared to the control group under horizontal head turns while walking ($p = 0.04$).

We have revised the introduction, statistical analysis, acknowledged in the study limitation that findings are preliminary and should be interpreted with caution, and moderated the conclusion accordingly.

Comment # 3. The authors should justify the use of a lumbar-worn accelerometer and use of linear accelerations to compute the Index of Harmonicity (IH). I am concerned that a lumbar sensor is a poor estimator of trunk accelerations. It is certainly less sensitive than (e.g.,) a sensor mounted at the thoracic spine. A lumbar sensor will not detect bending mid-torso.

Further, I question the author's interpretation of a lumbar sensor as measuring trunk accelerations. A lumbar sensor is likely a better estimate of pelvis acceleration, but a poor measure of both pelvis and lumbar accelerations. This could readily be amended by revising the manuscript's language.

As an aside, the oft-cited paper credited with first using the index of harmonicity (IH) used the pelvis and thoracic angles [1], though there does not appear to be strong support for one sensor placement versus another. Subsequent papers using a single lumbar sensor do not seem to evaluate the sensitivity of sensor configuration on the index of harmonicity.

[1] Lamoth CJ, Beek PJ, Meijer OG. Pelvis-thorax coordination in the transverse plane during gait. Gait & posture. 2002 Oct 1;16(2):101-14.

Response # 3. Previous studies have indicated that accelerometers can be placed either in the lower or upper trunk to measure trunk acceleration signals (1-4). In this study, we chose to mount an accelerometer at the lumbar because a systematic review has shown that trunk acceleration measured by a single triaxial accelerometer over the lower back level is a valid and reliable method for gait metrics analysis (4). Moreover, the single lumbar accelerometer used in the present study (DynaPort Hybrid, McRoberts, Netherlands) has also been valid and reliable for gait assessment across various populations (5-9). One explanation is that the lower back level approximates the body's center of mass, which is the internal representation of the whole-body movement during walking (10). Thus, the selection of a single reference point on the lower trunk is based on the assumption that the task of maintaining stability when walking primarily requires controlling the motion of the center of mass. However, we agree with the reviewer that adding another sensor, such as at the upper trunk level, may provide more insight into the role and pattern of trunk movement control contributing to gait stability. We have included the above information in the study limitation as follows (Page 18, line 347-349):

“In addition to the triaxial lumbar accelerometer employed in the present study, adding another sensor at the thoracic level would provide a further understanding of how upper and lower intersegmental coordination movements contribute to gait stability.”

As the reviewer mentioned, a previous study by Lamoth et al. (11) did not strongly support one sensor placement over another. In their study, thoracic and pelvis axial rotations during walking were measured using standard method as light-emitting diodes (LEDs) and the Selspot™ Camera System (Selcom). Afterward, Lamoth et al. established the spectral analysis method for assessing the coordination of thoracic and pelvis movement within a stride using the IH, in which a power ratio of 1 indicates that the intersegmental movement is perfectly harmonic. These findings indicate that the IH can reflect rhythmic axial intersegmental coordination during walking, potentially contributing to overall gait stability.

Regarding the evidence of the sensitivity of sensor configuration on the IH involves using a single lumbar sensor. Unfortunately, there is currently no study assessing the sensitivity of the single body-fixed sensor for IH, as this gait metric is gaining attention and is still an ongoing area of research. Nevertheless, a previous study revealed that the single lumbar accelerometer was a valid, reliable, and sensitive instrument for measuring spatiotemporal gait parameters in older adults (12). In addition, a systematic review has reported that single-point lower back accelerometers were acceptable for assessing dynamic gait outcomes, including the IH (4).

References:

1. Doi T, Hirata S, Ono R, Tsutsumimoto K, Misu S, Ando H. The harmonic ratio of trunk acceleration predicts falling among older people: results of a 1-year prospective study. J Neuroeng Rehabil. 2013;28:10:7.
2. Terrier P, Reynard F. Effect of age on the variability and stability of gait: a cross-sectional treadmill study in healthy individuals between 20 and 69 years of age. Gait Posture. 2015;41:170-4.
3. Menz HB, Lord SR, Fitzpatrick RC. Acceleration patterns of the head and pelvis when walking are associated with risk of falling in community-dwelling older people. J Gerontol A Biol Sci Med Sci. 2003;58(5):M446-52.

4. Mobbs RJ, Perring J, Raj SM, Maharaj M, Yoong NKM, Sy LW, et al. Gait metrics analysis utilizing single-point inertial measurement units: a systematic review. Mhealth. 2022;8:9.
5. Houdijk H, Appelman FM, Van Velzen JM, van der Woude LH, van Bennekom CA. Validity of DynaPort GaitMonitor for assessment of spatiotemporal parameters in amputee gait. J Rehabil Res Dev. 2008;45:1335-42.
6. Bautmans I, Jansen B, Van Keymolen B, Mets T. Reliability and clinical correlates of 3D-accelerometry based gait analysis outcomes according to age and fall-risk. Gait Posture. 2011;33:366-72.
7. de Bruin ED, Hubli M, Hofer P, Wolf P, Murer K, Zijlstra W. Validity and reliability of accelerometer-based gait assessment in patients with diabetes on challenging surfaces. J Aging Res. 2012;2012:954378.
8. Hartmann A, Luzi S, Murer K, de Bie RA, de Bruin ED. Concurrent validity of a trunk tri-axial accelerometer system for gait analysis in older adults. Gait Posture. 2009;29:444-8.
9. Hartmann A, Murer K, de Bie RA, de Bruin ED. Reproducibility of spatio-temporal gait parameters under different conditions in older adults using a trunk tri-axial accelerometer system. Gait Posture. 2009;30:351-5.
10. Yang F, Pai YC. Can sacral marker approximate center of mass during gait and slip-fall recovery among community-dwelling older adults? J Biomech. 2014;47(16):3807-12.
11. Lamoth CJ, Beek PJ, Meijer OG. Pelvis-thorax coordination in the transverse plane during gait. Gait Posture. 2002;16(2):101-14.
12. Werner C, Heldmann P, Hummel S, Bauknecht L, Bauer JM, Hauer K. Concurrent validity, test-retest reliability, and sensitivity to change of a single body-fixed sensor for gait analysis during rollator-assisted walking in acute geriatric patients. Sensors (Basel). 2020;20(17):4866.

Comment # 4. The language should be more specific the manuscript. For example, gait stability is invoked as rationale for evaluating IH, but not strongly connected to IH of the pelvis/lumbar spine and is not discussed in the context of the results. Additional justification for why pelvis/lumbar smoothness represents gait stability would also improve the manuscript.

Further, the rationale for using the IH in the context of fall prevention is poor: the IH is not a reliable predictor of falls [1]. How, then, can the authors claim that the IH during

a head turning task can be used for early fall detection without relating these results to fall risk?

[1] Riva F, Toebes MJ, Pijnappels MA, Stagni R, Van Dieën JH. Estimating fall risk with inertial sensors using gait stability measures that do not require step detection. Gait & posture. 2013 Jun 1;38(2):170-4.

Response # 4. Thank you for the suggestion. We have incorporated the connection between lumbar/pelvis movement, the IH, and gait stability into the rationale, as suggested by the reviewer. Furthermore, we have discussed these aspects in the context of the results within the discussion section (page 4-5, line 87-94; page 16, line 305-307).

We agree with the reviewer that the use of IH in the context of fall detection is poor. Therefore, we removed sentences about IH and early fall risk detection throughout the manuscript.

Comment # 5. The short walking trials used in this study are likely insufficient to extract stable measures of the IH [1]. Over one hundred strides are needed for the IH to approach a stable value in non-disabled adults. How can the authors trust their outcomes with only 20 m worth of strides of data per subject-trial? At a minimum, a bootstrapping procedure is needed to evaluate how cycle-to-cycle variations in gait and random sampling impact their results.

[1] Riva F, Bisi MC, Stagni R. Gait variability and stability measures: Minimum number of strides and within-session reliability. Computers in biology and medicine. 2014 Jul 1;50:9-13.

Response # 5. Several works have found that trunk acceleration-based measures are reliable for quantifying spatiotemporal gait parameters among healthy older adults and individuals with pathologic conditions (1-2); however, apart from Riva et al. (3), very limited studies investigate the reliability of trunk accelerometers on gait smoothness measures, including the IH. Moreover, no study has assessed cycle-to-cycle variations in the IH among older adults, both with and without cognitive limitations, especially under complex walking tasks.

As the reviewer's concern regarding the stable measures of the IH, we further explored the consistency of IH between the first and second trials for each walking condition in

both the MCI and control groups, using test-retest reliability. The results showed good to excellent test-retest reliability of the IH for all walking conditions (as shown in Table 1).

Table 1 The intra-class correlation coefficient of the IH

Walking condition	Control group				MCI group			
	IH (Mean ± SD) 1st trial	IH (Mean ± SD) 2nd trial	ICC 3,1 (95% CI)	p-value	IH (Mean ± SD) 1st trial	IH (Mean ± SD) 2nd trial	ICC 3,1 (95% CI)	p-value
**Walking forward (baseline)**								
IH-AP	0.70 ± 0.08	0.67 ± 0.08	0.921	0.0001*	0.70 ± 0.12	0.67 ± 0.11	0.902	0.0001*
IH-ML	0.75 ± 0.09	0.75 ± 0.08	0.800	0.001*	0.75 ± 0.12	0.73 ± 0.12	0.908	0.0001*
IH-VT	0.86 ± 0.05	0.84 ± 0.07	0.913	0.0001*	0.85 ± 0.05	0.83 ± 0.05	0.898	0.0001*
**Walking a narrow path**								
IH-AP	0.76 ± 0.07	0.75 ± 0.08	0.855	0.0001*	0.74 ± 0.12	0.74 ± 0.11	0.928	0.0001*
IH-ML	0.77 ± 0.09	0.75 ± 0.10	0.820	0.0001*	0.74 ± 0.10	0.71 ± 0.12	0.879	0.0001*
IH-VT	0.22 ± 0.14	0.20 ± 0.14	0.930	0.0001*	0.24 ± 0.14	0.23 ± 0.14	0.960	0.0001*
**Walking around an obstacle**								
IH-AP	0.17 ± 0.10	0.20 ± 0.15	0.858	0.0001*	0.20 ± 0.15	0.20 ± 0.15	0.956	0.0001*
IH-ML	0.53 ± 0.15	0.52 ± 0.15	0.835	0.0001*	0.52 ± 0.17	0.49 ± 0.19	0.959	0.0001*
IH-VT	0.39 ± 0.20	0.34 ± 0.21	0.837	0.0001*	0.37 ± 0.19	0.35 ± 0.19	0.963	0.0001*
**Horizontal head turns while walking**								
IH-AP	0.38 ± 0.23	0.35 ± 0.24	0.961	0.0001*	0.38 ± 0.21	0.34 ± 0.19	0.761	0.003*
IH-ML	0.73 ± 0.12	0.74 ± 0.12	0.958	0.0001*	0.63 ± 0.16	0.64 ± 0.17	0.974	0.0001*
IH-VT	0.67 ± 0.18	0.70 ± 0.16	0.899	0.0001*	0.59 ± 0.19	0.62 ± 0.16	0.898	0.0001*
**Vertical head turns while walking**								
IH-AP	0.60 ± 0.19	0.62 ± 0.20	0.968	0.0001*	0.60 ± 0.19	0.60 ± 0.18	0.926	0.0001*
IH-ML	0.69 ± 0.16	0.71 ± 0.16	0.947	0.0001*	0.66 ± 0.15	0.66 ± 0.15	0.953	0.0001*
IH-VT	0.72 ± 0.18	0.73 ± 0.14	0.919	0.0001*	0.63 ± 0.16	0.62 ± 0.16	0.915	0.0001*

*p-value < 0.05

ICC = intra-class correlation coefficient

IH = Index of harmonicity; IH-AP = Index of harmonicity in the anteroposterior direction; IH-ML = Index of harmonicity in the mediolateral direction; IH-VT = Index of harmonicity in the vertical direction

The results from Table 1 indicate an acceptable reliability of the IH measures using trunk accelerometers obtained from short walking trials. Currently, several studies determine gait smoothness, including the IH and Harmonic Ratio (HR), in older adults

and individuals with pathologic conditions within a short distance range from 8 to 30 meters (4-7). However, we agree with the reviewer that adding the number of strides by increasing the walking distance would allow for stable gait outcomes. We acknowledged that it is a limitation of the study and stated in the manuscript (page 18, line 349-350) as follows:

“Moreover, a long walking distance that covers more strides would allow for a steady gait outcome analysis.”

References:

1. Kobsar D, Charlton JM, Tse CTF, Esculier JF, Graffos A, Krowchuk NM, et al. Validity and reliability of wearable inertial sensors in healthy adult walking: a systematic review and meta-analysis. J. Neuroeng Rehabil. 2020;17:62.
2. Werner C, Heldmann P, Hummel S, Bauknecht L, Bauer JM, Hauer K. Concurrent validity, test-retest reliability, and sensitivity to change of a single body-fixed sensor for gait analysis during rollator-assisted walking in acute geriatric patients. Sensors (Basel). 2020;20(17):4866.
3. Riva F, Bisi MC, Stagni R. Gait variability and stability measures: minimum number of strides and within-session reliability. Comput Biol Med. 2014;50:9-13.
4. Simonetti E, Pillet H, Vannozzi G, Loiret I, Villa C, Bascou J, et al. Investigating symmetry in amputee gait through the improved harmonic ratio: influence of the stride segmentation method. Comput Methods Biomech Biomed Engin. 2019;22(sup1):S221-S3.
5. Micó-Amigo ME, Kingma I, Heinzel S, Nussbaum S, Heger T, van Lummel RC, et al. Dual vs. single tasking during circular walking: what better reflects progression in Parkinson's disease? Front Neurol. 2019;10:372.
6. Pau M, Mulas I, Putzu V, Asoni G, Viale D, Mameli I, et al. Smoothness of gait in healthy and cognitively impaired individuals: a study on Italian elderly using wearable inertial sensor. Sensors (Basel). 2020;20(12):3577.
7. Javadpour S, Sinaei E, Salehi R, Zahednejad S, Motealleh A. Comparing the effects of single-task versus dual-task balance training on gait smoothness and functional balance in community-dwelling older adults: a randomized controlled trial. J Aging Phys Act. 2022;30(2):308-15.

Comment # 6. The authors should specify if the trial order was randomized.

Response # 6. Thank you. The trial order was randomized. We have added this statement in the method section (page 10, line 206-207).

Comment # 7. The authors should consider how instructions for the head turning task may impact IH. Per the instructions, the rate of head turning would differ based on each participant's walking speed ("head turn every 1 meter" [sic]). As gaze stabilization performance is associated with walking performance in adults with vestibular dysfunction [1] and head turning alters gait stability [2], it is plausible that varying the rate of head rotation would differentially alter gait stability and/or smoothness in people with different walking speeds.

[1] Whitney SL, Marchetti GF, Pritcher M, Furman JM. Gaze stabilization and gait performance in vestibular dysfunction. Gait Posture. 2009 Feb;29(2):194-8. doi: 10.1016/j.gaitpost.2008.08.002. Epub 2008 Sep 23. PMID: 18815040; PMCID: PMC4879829.

[2] Fitzgerald C, Thomson D, Zebib A. et al. A comparison of gait stability between younger and older adults while head turning. Exp Brain Res. 238, 1871-1883 (2020). https://doi.org/10.1007/s00221-020-05846-3

Response # 7. Thank you for your point of view. We agree with the reviewer that the rate of head turning while walking relates to walking speed, which may result in gait stability. In the present study, we consistently gave a standard instruction for participants to turn their heads as fast and as far as possible in response to the command every 1 meter. With this, there was no discernible difference in the head turning rate between individuals or walking trials. As we found, all participants could follow the instructions and complete the walking tasks without obviously changing gait speed from baseline. Moreover, the results showed that gait speed in head turning conditions was similar between the MCI and control groups, suggesting that the difference in the IH between the two groups was not influenced by gait speed (Table 2 in the manuscript).

Comment # 8. I am surprised to see such small differences in Trails B-A scores between groups. In these tasks, I might expect set-shifting ability to correlate with changes in IH. The authors may see larger effects of head turning or obstacle

negotiation if groups differed in clinical scores associated with set-shifting or motor-cognitive integration.

Response # 8. We agree with the reviewer that larger effects of head turning or obstacle negotiation might be observed if Trail B-A scores were differences between the two groups. The head turning or obstacle negotiation while walking serves as a motor dual-tasking as participants have to allocate their attention between walking and a head turning or obstacle negotiation task. Non-significant differences in TMT B-A scores in the present study are likely due to a large variation of the data.

Comment # 9. In general, the Discussion is not strongly focused on interpreting and contextualizing the study's findings. For example, paragraph 2 could be written almost entirely without conducting the study. Condensing the Discussion and improving focus on contextualizing results would improve the manuscript.

Response # 9. We appreciate your constructive feedback. We have re-written the discussion to focus on interpretating and contextualizing the study findings. Thank you once again for your valuable suggestion.

Reviewer # 3:

The paper provides valuable insights into the assessment of gait changes in older adults with Mild Cognitive Impairment (MCI) under complex motor tasks. Here is a review of the introduction and methodology sections:

Comment # 1.

Introduction Review:

The introduction sets the stage effectively by discussing the importance of executive function, attention, and their connection to safe mobility. The rationale for the study, focusing on the potential for gait abnormalities in individuals with MCI, is well-explained. This establishes the significance of the research. The introduction highlights the existing research and points out discrepancies, which helps justify the need for the study. The introduction clearly defines terms like MCI, making it reader-friendly for those not well-versed in the subject matter. The study's objectives and hypotheses are clearly stated, providing a solid foundation for the research.

Response # 1. Thank you very much.

Comment # 2.

Methodology Review:

The methodology section adequately describes the participant selection process and sample size calculation, contributing to the paper's transparency. Inclusion and exclusion criteria are well-defined, ensuring that the study focuses on the intended population. Ethical considerations and approval from the institution's review committee are appropriately mentioned, underlining the ethical aspects of the research. The assessment of gait smoothness under complex motor tasks is well-explained, including the use of a triaxial accelerometer device, making it clear to the reader how data was collected. The statistical analysis section is detailed and provides a clear understanding of how the data was analyzed. The results are presented in a clear and organized manner, with tables that effectively summarize the findings.

Response # 2. Thank you very much.

Comment # 3. General Review:

The paper is generally well-structured and follows a logical flow, with each section building upon the previous one. The paper maintains a strong focus on the research question throughout the introduction and methodology sections. The language is technical but clear, making the content accessible to a broader audience. The limitations are acknowledged, and the need for further research is suggested, showing the author's awareness of the study's boundaries.

In summary, the introduction effectively introduces the research problem and its significance, while the methodology provides a clear framework for the study, including participant selection and data collection methods. The paper appears to be well-structured and technically sound, making it a valuable contribution to the field of research on gait abnormalities in older adults with MCI.

Response # 3. Thank you very much.
