# Supplementary material for: Gait smoothness during high-demand motor walking tasks in older adults with mild cognitive impairment
Source: PLoS One. 2024 Jan 19;19(1):e0296710. doi: 10.1371/journal.pone.0296710 (PMC10798528; doi:10.1371/journal.pone.0296710)
Supplement: Attachment — Submitted filename: Response to Reviewers.pdf [file pone.0296710.s005.pdf]

Reviewers' comments:

Reviewer's Responses to Questions

Reviewer # 1:

Comment # 1. Should consider stating "may lead..." since walking smoothness does not necessarily conflate with stability, does it? Furthermore, you did not report that participants lost balance during their walks suggesting that they were not unstable.

Response # 1: Thank you for the suggestion. We have revised the words according to the reviewer's suggestion (Page 16, line 312).

Comment # 2. Your findings suggest that older healthy groups should be compared to young healthy groups with regard to IH.

Response # 2: Thank you for pointing this out. We have revised the sentence as the reviewer's suggestion as follows (Page 17, line 337-340):

"Studies examining gait smoothness concerning IH during narrow path walking among individuals with cognitive limitations compared to healthy older and young adults are limited, making it challenging to compare the findings."
